# What do brain endocasts tell us? A comparative analysis of the accuracy of sulcal identification by experts and perspectives in palaeoanthropology

**DOI:** 10.1111/joa.13966

**Published:** 2023-11-07

**Authors:** Nicole Labra, Aurélien Mounier, Yann Leprince, Denis Rivière, Mélanie Didier, Eric Bardinet, Mathieu D. Santin, Jean François Mangin, Andréa Filippo, Lou Albessard‐Ball, Amélie Beaudet, Douglas Broadfield, Emiliano Bruner, Kristian J. Carlson, Zachary Cofran, Dean Falk, Emmanuel Gilissen, Aida Gómez‐Robles, Simon Neubauer, Alannah Pearson, Carolin Röding, Yameng Zhang, Antoine Balzeau

**Affiliations:** ^1^ Département Homme et Environnement UMR 7194, CNRS, PaleoFED Team, Muséum national d’Histoire naturelle Paris France; ^2^ Turkana Basin Institute Nairobi Kenya; ^3^ Université Paris‐Saclay, CEA, CNRS UMR 9027, Baobab, NeuroSpin Gif‐sur‐Yvette France; ^4^ ICM—Institut du Cerveau, Hôpital Pitié‐Salpêtrière, Centre de NeuroImagerie de Recherche—CENIR Paris France; ^5^ Department of Archaeology PalaeoHub, University of York York UK; ^6^ Laboratoire de Paléontologie, Évolution, Paléoécosystèmes et Paléoprimatologie (PALEVOPRIM), UMR 7262 CNRS Université de Poitiers Poitiers France; ^7^ Department of Cell Biology University of Miami Miami Florida USA; ^8^ Paleobiología, Centro Nacional de Investigación sobre la Evolución Humana Burgos Spain; ^9^ Evolutionary Studies Institute University of the Witwatersrand, Palaeosciences Centre Johannesburg South Africa; ^10^ Department of Integrative Anatomical Sciences, Keck School of Medicine University of Southern California California Los Angeles USA; ^11^ Anthropology Department Vassar College Poughkeepsie New York USA; ^12^ Department of Anthropology Florida State University Tallahassee Florida USA; ^13^ Department of African Zoology Royal Museum for Central Africa Tervuren Belgium; ^14^ Department of Anthropology University College London London UK; ^15^ Institute of Anatomy and Cell Biology Johannes Kepler University Linz Linz Austria; ^16^ School of Archaeology and Anthropology Australian National University Canberra Australian Capital Territory Australia; ^17^ Paleoanthropology, Institute for Archaeological Sciences and Senckenberg Centre for Human Evolution and Paleoenvironment Eberhard Karls University of Tübingen Tübingen Germany; ^18^ Institute of Cultural Heritage Shandong University Qingdao Shandong China

**Keywords:** brain endocast, brain evolution, palaeoneurology, sulcal identification

## Abstract

Palaeoneurology is a complex field as the object of study, the brain, does not fossilize. Studies rely therefore on the (brain) endocranial cast (often named endocast), the only available and reliable proxy for brain shape, size and details of surface. However, researchers debate whether or not specific marks found on endocasts correspond reliably to particular sulci and/or gyri of the brain that were imprinted in the braincase. The aim of this study is to measure the accuracy of sulcal identification through an experiment that reproduces the conditions that palaeoneurologists face when working with hominin endocasts. We asked 14 experts to manually identify well‐known foldings in a proxy endocast that was obtained from an MRI of an actual in vivo *Homo sapiens* head. We observe clear differences in the results when comparing the non‐corrected labels (the original labels proposed by each expert) with the corrected labels. This result illustrates that trying to reconstruct a sulcus following the very general known shape/position in the literature or from a mean specimen may induce a bias when looking at an endocast and trying to follow the marks observed there. We also observe that the identification of sulci appears to be better in the lower part of the endocast compared to the upper part. The results concerning specific anatomical traits have implications for highly debated topics in palaeoanthropology. Endocranial description of fossil specimens should in the future consider the variation in position and shape of sulci in addition to using models of mean brain shape. Moreover, it is clear from this study that researchers can perceive sulcal imprints with reasonably high accuracy, but their correct identification and labelling remains a challenge, particularly when dealing with extinct species for which we lack direct knowledge of the brain.

## Graphical abstract

Palaeoneurology is a complex field as the object of study, the brain, does not fossilize. We asked 14 experts to manually identify foldings in a proxy endocast obtained from an MRI of an actual in vivo *Homo sapiens*. Researchers can perceive sulcal imprints with reasonably high accuracy, but their correct identification and labelling remains a challenge, particularly when dealing with extinct species for which we lack direct knowledge of the brain.

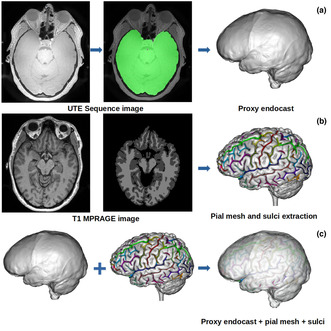

## 1 INTRODUCTION

Palaeoneurology is a controversial and complex field in palaeoanthropology for several reasons. The principal one is that the object of study, the brain, does not fossilize. Thus, to study the evolution of the human brain, palaeoanthropologists must analyse the (brain) endocranial cast (i.e. generally named endocast in palaeontology) which is an internal moulding of the skull that reflects the imprints that the brain left on the intracranial walls. The endocast is the only available and reliable proxy for brain shape and size. It may also reproduce some of the grooves (sulci) that are present on the surface of the cerebral cortex and often border convolutions and/or functional regions (Connolly, 1950; Holloway et al., 2004; Le Gros Clark et al., 1936). In any case, endocasts provide the most direct and precise information available for brains of extinct species (e.g. Bruner, 2019; Holloway et al., 2004; Jerison, 1973). Traditionally, the replica of the internal cranial cavity was obtained by filling the empty braincase with latex and using the latter as a mould to generate a plaster endocast. Nowadays, ‘virtual’ or ‘digital’ endocasts can be generated using manual or automatic segmentation methods with CT or microCT data, thus providing new opportunities for the study of brain evolution. More specimens are therefore potentially available, including the most complete ones which provide the most information, which could prove more difficult to mould for prehistoric specimens. The analytical perspectives are much broader and contributions of imaging methodologies for the reproducibility and repetitiveness of palaeoneurological works are obvious.

However, researchers debate whether or not specific marks found on endocasts correspond reliably to particular sulci and/or gyri of the brain that were imprinted in the braincase (e.g. Balzeau & Mangin, 2021; Dumoncel et al., 2021; Falk, 2014). The external surface of endocasts display different sets of marks. Some are bulges that correspond either to the course and indentations of the cranial sutures or to the imprints of the middle meningeal system, venous sinuses and other parts of the blood drainage system that are in close contact with the internal surface of the braincase. Finally, shallow grooves over the surface are generally considered as indications of underlying brain sulci. Some variation is visible in the number and degree of expression of those grooves among primate species and during development on endocasts. Smaller brained primate species and younger individuals tend to show more details than larger brain species and adults respectively (e.g. Balzeau et al., 2005; Falk, 2014). In this general context, the fundamental question remains: Are such marks really related to sulcal imprints or to something else? By extension, we cannot be sure to what extent it is possible to reconstruct sulcal morphology from endocasts. Previous studies have tried to address this problem by using magnetic resonance imaging (MRI) (Dumoncel et al., 2021). For example, Dumoncel et al. (2021) found a close correspondence between morphological features of the human brain and the corresponding endocast, with the exception of the superior region of the frontal and parietal lobes. In this study, the comparison between the brain and the endocast was made from different imaging acquisitions that relied on MRI and CT data. Those methodologies have different geometric constraints and different resolutions that cannot be overcome by the alignment of the two datasets. Further, the detection of the sulcal imprints was automatic, identification of other features were manual, the brain hull had to be deformed to match the shape of the endocast and the study included a restricted sample of five individuals. While similar approaches are commonly used in neuroscience research, they are much less commonly applied to the study of hominin endocasts. The interesting results of Dumoncel et al. open new perspectives. In this context, several issues remain to be addressed such as the accuracy of sulcal identifications reported in the palaeoneurology literature from the study of endocranial casts. This perspective concerns both not only the ability to recognize structures that are actually anatomically linked between the brain and the endocranium, but also the ability of observers to recognize and identify them in a valid way.

The aim of this study is, thus, to measure the accuracy of sulcal identification through an experiment that closely reproduces the conditions that palaeoneurologists face when working with hominin endocasts. The approach here is different from previous works (de Jager et al., 2022; Dumoncel et al., 2021), but complementary. By combining classic palaeoneurological approaches with new techniques available in the neuroimaging field, our work aims at answering the following questions: Is it possible to obtain an endocast or a similar proxy that reproduces external details of the human/hominid brain from an MRI scan? How accurately and consistently do contemporary researchers identify imprints on brain endocasts? Is it possible to accurately identify the gyri and sulci of a human/hominin brain from the marks visible on an endocast? And finally, are there regions of the endocast/brain in which these features cannot be reconstructed and/or can be better recognized?

We asked 14 researchers (LAB, ABe, DB, EB, KC, ZC, DF, EG, AG, SN, AP, CR, YZ, Aba) with experience in the study of the brain, including working with brain endocasts and knowledge about the evolution of the human brain, to manually identify well‐known foldings in a proxy endocast that was obtained from an MRI of an actual in vivo *Homo sapiens* brain. We tried to make the conditions of the experiment as similar as possible to a typical fossil endocast assessment by providing researchers with one single endocast that they would need to assess without comparison with other individuals from the same population. While this approach does not allow for a quantification of the variation of accuracy across different individuals, it reproduces more closely the conditions of sulcal assessments in fossil hominins. Moreover, by using MRI data obtained in the same conditions and at the same time to reconstruct the brain and the endocast we minimize methodological factors that can introduce bias in the comparison of models obtained with different methodologies and in different conditions.

Our main objective was to quantify how accurately and consistently researchers identify the gyri (or convolutions) and sulci that are present on the human brain on a corresponding endocast. In this context we aimed first to establish whether folding patterns can be reliably reconstructed from human endocasts, and second, to identify which sulci and gyri may be reliably and accurately identified on endocasts and which may not. We also investigated the limitations of applying the nomenclature for brain morphology derived from an atlas approach to the analysis of particular specimens. This aspect has some implications not only in the field of neurosciences but is also central in palaeoneurology. Our hope is that the information gathered in this work will help the palaeoanthropological community to critically evaluate palaeoneurological inferences published in the past and to provide a new framework and best‐practice guidance for future research.

## 2 MATERIALS AND METHODS

Fourteen researchers from the fields related to the study of human brain evolution accepted an invitation to contribute to this work. Most of them have expertise in the study of human/hominin endocasts (LAB, ABe, EB, KC, DF, SN, CR, YZ, Aba), whereas others have expertise in the study of the primate brain (DB, ZC, EG, AG, AP). Several have a broad experience in the field of palaeoneurology.

**FIGURE 1:**
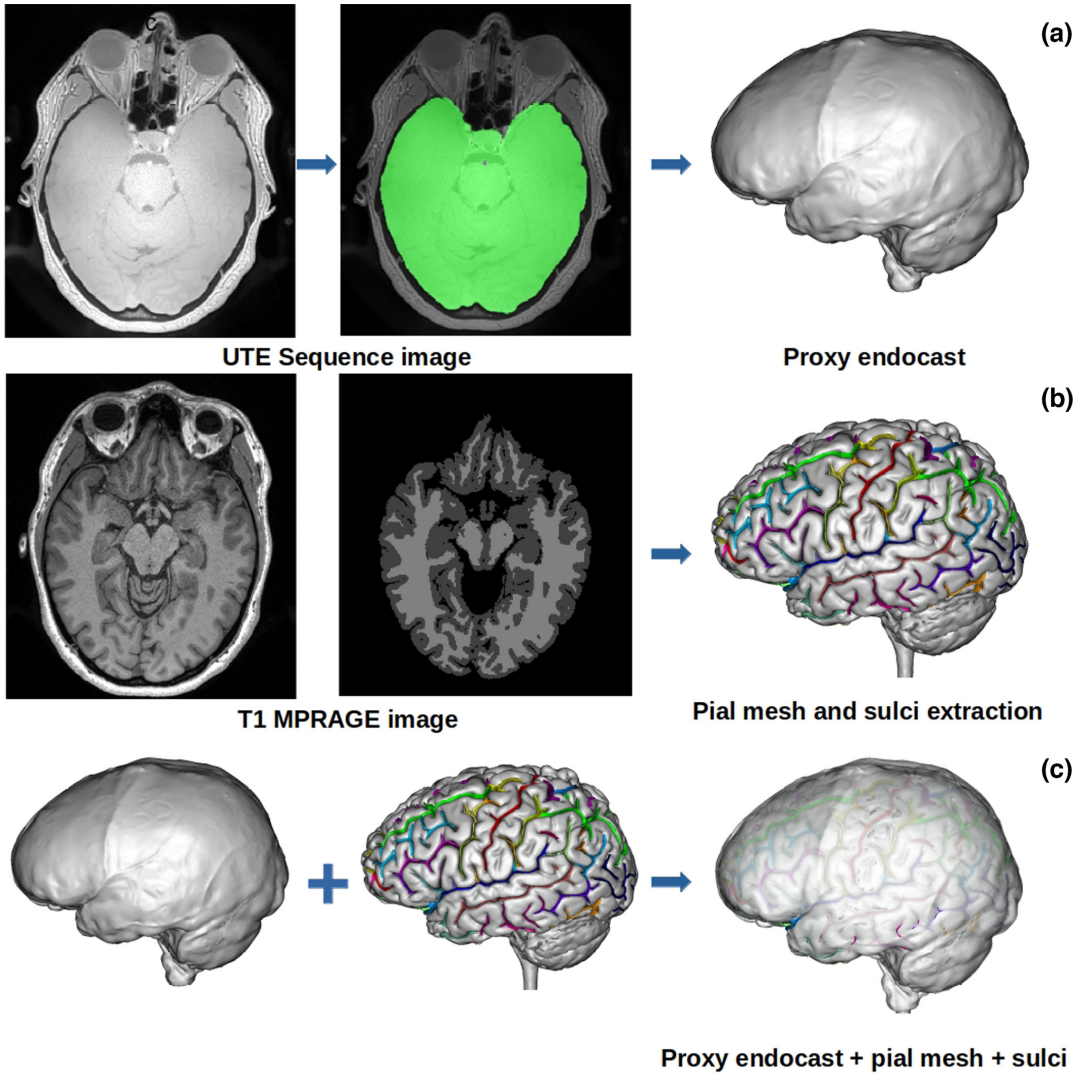
Brain structures and meshes used for manual labelling of sulci. (a) Proxy endocast obtained from an MRI UTE image. (b) Structures (pial mesh and automatic sulci extraction) obtained from the brain segmentation using a T_1_‐MPRAGE image. (c) Superimposition of the proxy endocast, the pial mesh and the automatically extracted sulci used for the evaluation of manual sulcal identification.

**FIGURE 2:**
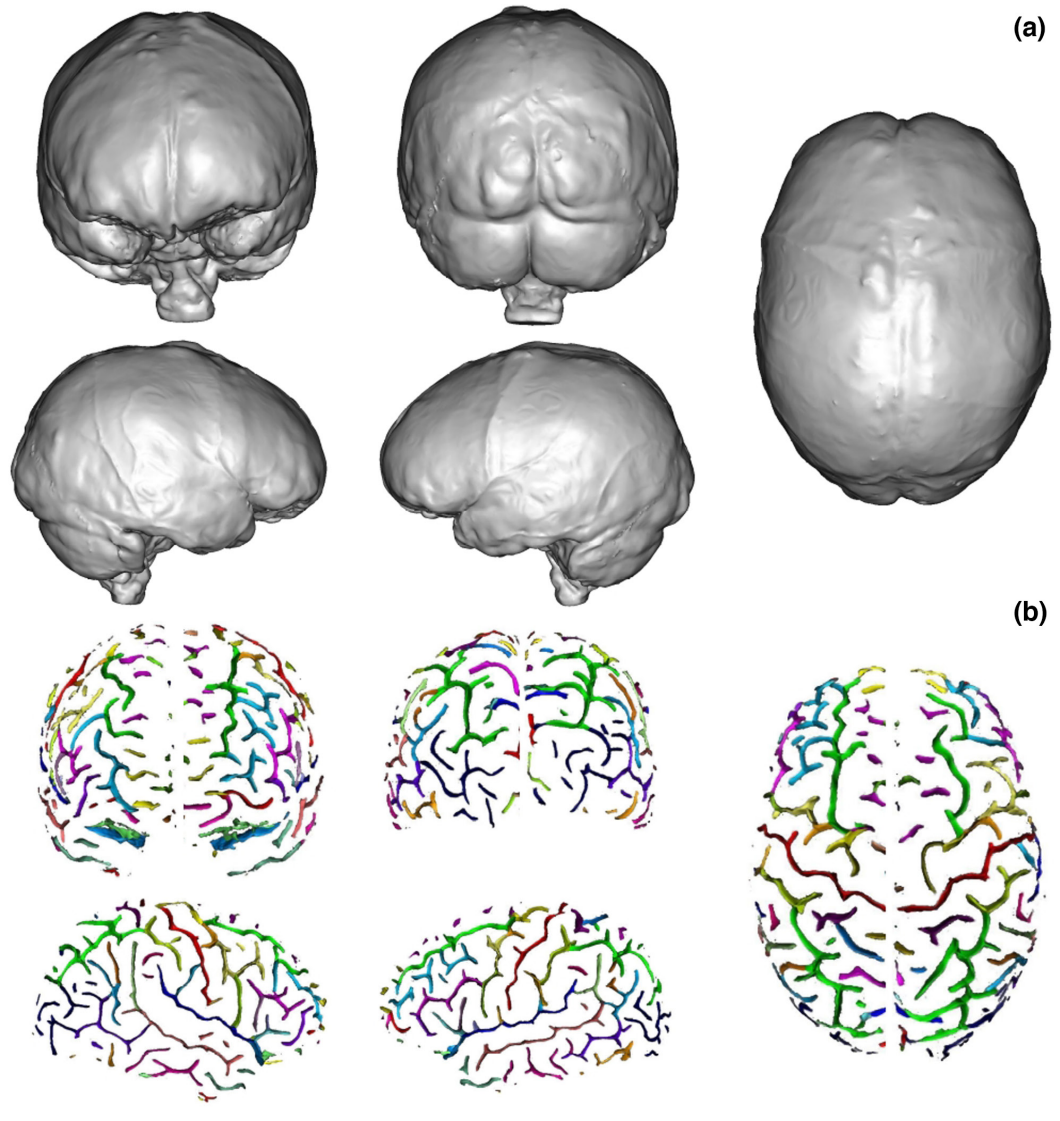
Brain views used in this study. (a) The five images of the endocast and (b) the same five images showing the sulci obtained from the actual brain through the automatic sulci extraction process (see Table 1 and Figure 3 for the definition of the sulci and colour correspondence). From left to right, from superior to inferior: anterior view, posterior view, right central, right hemisphere view and left hemisphere view.

**FIGURE 3:**
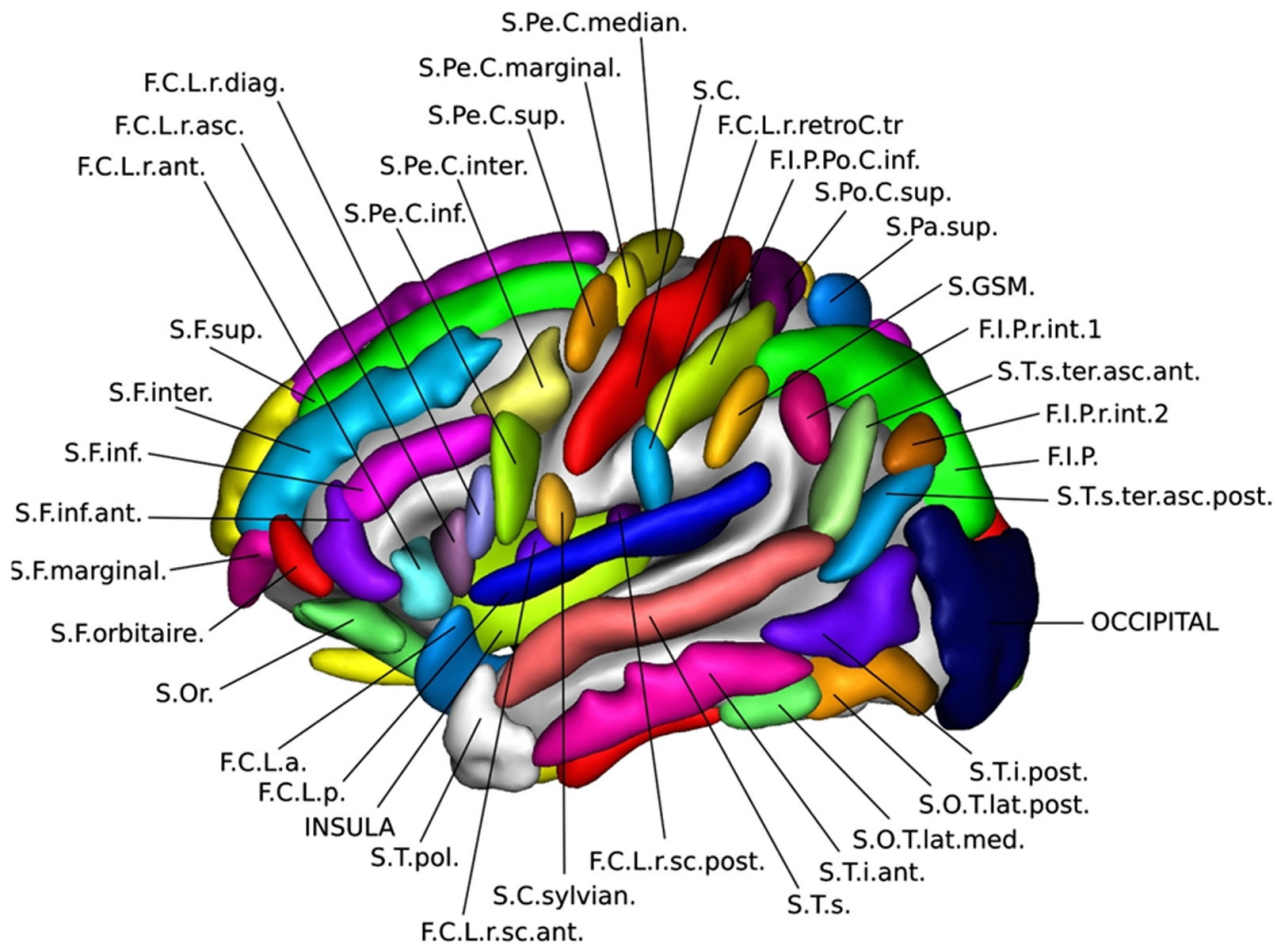
Nomenclature for sulci labelling used by Morphologist toolbox of BrainVISA (Perrot et al., 2011).

MRI acquisitions took place in the Center for Neuroimaging Research, Brain Institute, Pitié‐Salpêtrière Hospital, Paris, France. The ‘Comité de Protection des Personnes Sud‐Méditerranée II’ approved the research protocol for the imaging centre that was used in this study (comity reference 221 B38, identification number 2021‐A02404‐37). A proxy endocast was generated from a 34‐year‐old female volunteer using the image obtained with an ultrashort time‐to‐echo (UTE) MRI sequence (Robson et al., 2003), as shown in Figure 1a. The model is very similar to those generally obtained from CT data in terms of visible external morphology. Contributors were provided with a 3D model of this endocast, standard 2D projections for labelling (Figure 2a) and a labelled reference (Figure 3) for comparison. In the same imaging session, a T_1_‐MPRAGE image was acquired and used to segment the brain, to generate the pial surface mesh and to extract and automatically label the sulci (Figure 1b). The combination of the automatically labelled sulci superimposed with the pial mesh and with the proxy endocast was used as a reference (Figure 1c) in the evaluation and analysis of the manually labelled sulci made by the researchers. Both the generation of the proxy endocast and brain segmentation were carried out using the Morphologist toolbox from BrainVISA (Cointepas et al., 2001; Fischer et al., 2012). The automatically assigned sulcal labels used as reference in this study are the same as used by Morphologist (Perrot et al., 2011). Several nomenclatures or atlases exist for brain sulci in humans (e.g. Ono et al., 1990), however our purpose here is not directly to address this aspect and by using the referential included in the software we minimize the methodological issues all along the analytical process.

Contributors were asked to draw the grooves/sulci they could identify on the 3D virtual endocast on five images corresponding to five different planes/views (Figure 2a) of the proxy endocast with the help of an online drawing tool called Sketchpad.^1^ We chose this online tool due to its ease of use, which allowed all participants to make their drawings as requested. The actual sulcal pattern configuration for corresponding views from the actual brain were also obtained for comparison, but not provided to contributors (Figure 2b). We have not included an inferior view of the endocast for different main complementary reasons. The surface of the endocast in this view does not show any of the main sulci of the brain, while the cerebellum hides details of the posterior part of the temporal lobes and all the inferior extension of the occipital lobes. Moreover, those areas are exceptionally preserved in the fossil hominin record. For these reasons, we have estimated that the expert would not have been able to detail much information in inferior view.

The images of the five views of the endocast in .sketchpad format and a mesh (.ply format) for a 3D full view were sent to the researchers. In order to obtain comparable identifications, enable drawing and labelling of the sulci and facilitate the process of comparison and data analysis, a short tutorial on the use of Sketchpad, together with a list of 42 sulci that could potentially be identified in the proxy endocast and their nomenclature, were also included. Thereby, identification, drawing and labelling of the sulci were enabled in as consistent a manner as possible, which facilitated the process of comparison and data analysis. The researchers were asked to manually label the grooves or sulci they could identify in this proxy endocast on the five views in order to facilitate the comparative analysis of the data between all the researchers. However, they also had the possibility of manipulating and visualizing the original 3D model in order to secure their observation and labelling on the different 2D views.

Each sulcus identified in each of the five views by each participant was later isolated and stored (in groups by view) as independent images that preserved the position and size of the manually drawn sulci. The same sulcus isolation process was also carried out with the reference automatically extracted sulci obtained by Morphologist. The sulcus isolation allowed a one‐to‐one image comparison between the manually identified sulci and the automatically extracted sulci, which we use to represent the real sulcal configuration of this individual. We carried out this comparison by using two different similarity measures: the Dice index (Sørensen, 1948), which quantifies the overlapping between sulci pixel by pixel and the mean minimum Euclidean distance, a point‐by‐point measure of the similarity in shape and closeness between a reference sulcus and a manually labelled sulcus.

As the sulci were manually identified and drawn by the researchers in five different views of the endocast, the comparisons and analyses of the manually labelled sulci with the reference sulci were performed with the same tools and process, but independently in each view. It is therefore necessary to have the information from all the views in order to cross‐check all the available information before reaching a conclusion regarding the feasibility of identifying a particular groove or sulcus from the endocast comparison.

The visual identification of the grooves on the endocasts and their labelling as particular sulci are two different and complex processes that might create conflict and bias in the sulcal comparisons if they are considered together. For example, a groove can be successfully identified as a sulcal imprint on the endocast by multiple researchers, but it can be incorrectly labelled by some, so the sulcal identification can be right even if the labelling is wrong. To avoid this problem, the evaluation and analysis were carried out successively following two different perspectives. First, the labels and sulci were considered as they were identified by the researchers in order to evaluate the labelling and relative position of the sulci identified manually. Second, the wrong labels associated with well‐identified grooves were corrected in order to evaluate the capacity to identify the presence of sulci from the anatomical changes in the surface of the proxy endocast.

Labelled images provided by each researcher were anonymized before analysis. We will refer to them as Researcher X, abbreviated as RX, where X corresponds to a number between 1 and 14 assigned randomly to each one of them.

### 2.1 Brain segmentation and proxy endocast creation

In the context of the PaleoBrain project,^2^ a high‐resolution 3D T_1_‐weighted structural image (MPRAGE) and a UTE sequence image were acquired from one healthy female volunteer (right‐handed, 34‐year‐old) by the Paris Brain Institute (ICM), in January 2021. All the images were acquired using a 3 T Prisma Fit (Siemens, Germany) and a 64‐channel head coil during the same session. For the MPRAGE T_1_ image, the protocol included a high‐resolution 3D T_1_‐weighted acquisition using an MPRAGE sequence (TE = 2.2 ms, TR = 2400 ms, TI = 1000 ms, FA = 8, matrix = 256 × 320 × 320, voxel size = 0.7 × 0.7 × 0.7 mm). For the UTE sequence image, the protocol included a high‐resolution UTE sequence acquisition (TE = 70 μs, TR = 5 ms, FA = 3, matrix = 416 × 416× 416, voxel size = 0.59 × 0.59 × 0.59 mm). T_1_‐MPRAGE and the UTE sequence were used in the brain segmentation and the endocast creation process respectively.

Brain segmentation was performed with the Morphologist toolbox of BrainVISA using the T_1_‐MPRAGE image. The entire pipeline (shown in Figure S1) was executed in order to segment the brain into different structures: pial surface mesh and sulci (Figure S1).

The UTE pulse sequence is an MRI technique used for imaging tissues or tissue components with short transverse relaxation time, so‐called T_2_ (Robson et al., 2003). Thus, the cortical bone, tendons, ligaments and brain among other tissues will show high signals. The different brain tissues are not easily distinguishable in the image obtained from the UTE sequence (Figure 1a, using the protocol described earlier) when comparing with the traditional T_1_‐MPRAGE (Figure 1b). We take advantage of this homogeneity in the signal of the whole brain tissue within the dura mater to mask it and create a mesh from it.

In order to obtain the mask of all the brain tissue within the dura mater, the same Morphologist pipeline used for the brain segmentation (and shown in the Figure 3) was applied. However, this time the UTE sequence image was used and only the first four steps were necessary to obtain the endocranial mask. Some parameters were adjusted until obtaining a mask that fully covered all the structures within the dura mater, instead of obtaining a mask of the structures within the pial surface, as the algorithm usually does. Once the mask was obtained, we used the BrainVISA environment to compute the mesh using the AimsMeshBrain command of the AIMS tools (Analysis of Images and Signal for Neuroimaging tools). As the dura mater is the membrane that surrounds the brain and separates it from the cranial bone, the mesh obtained from it comprises all the cranial cavity and is highly similar to the actual endocasts obtained using CT, as illustrated in the Figure 2a. One possible difference between our proxy endocast from MRI and endocasts obtained using CT is at the level of resolution. In this case, the size of the voxels of the MRI data is around 0.6 mm. Medical CT might provide slightly smaller voxels, but resulting 3D models of endocasts are generally of the same range of resolution. MicroCT is also now widely used to obtain 3D endocast with a voxel size than can be around 0.15 mm, however this method cannot be applied to living volunteers. This difference is important and has some influence on the ability to identify imprints on virtual models. Nevertheless, the 3D model of the endocast obtained here with the UTE sequence has a resolution comparable with what can be obtained from medical CT scans, and is therefore similar to that of most virtual endocasts of fossil hominins studied in the past 40 years.

One important aspect in the way we have designed the methodology for this study is that the data that we use to reconstruct the brain and the endocast were acquired with the same machine, in the same position, during the same imaging session. Moreover, the different sequences for the IRM data were segmented with the same software. Our general objective was to minimize variations and errors related to the experimentation and reconstruction of the data in order to optimize the degree of precision of the resulting anatomical information. The 3D models of the endocast and of the brain are available on simple request to the corresponding author.

### 2.2 Sulci to identify

Given the complexity of inter‐subject brain anatomical variability and the large number of available nomenclatures associated with the organization of brain sulci, we provided participants with a common standardized nomenclature for specific sulci that could potentially be identified in the proxy endocast. Those sulci were selected by visual comparison of the proxy endocast with the actual sulci extraction and pial meshes for both hemispheres obtained by the segmentation of the MRI image using Morphologist, as described earlier. The nomenclature used for each sulcus corresponds to the atlas used by Morphologist for the automatic labelling of sulci in the segmentation process (Perrot et al., 2011) and it is shown in Figure 3. A summary of the sulci proposed for this study as well as the corresponding nomenclature is presented in Table 1.

**TABLE 1:** List of the sulci that can be potentially identified and their corresponding nomenclature.

Main sulci					
No.	Abbreviation	Sulci name	No.	Abbreviation	Sulci name
1	S.C	*Central sulcus*	2	S.C.sylvian	*Central sylvian sulcus*
3	F.C.L (a and p)	*Lateral fissure (anterior and posterior parts)*	4	F.C.L.r.ant.	*Anterior ramus of the lateral fissure*
5	F.C.L.r.asc	*Ascending ramus of the lateral fissure*	6	F.C.L.r.diag.	*Diagonal ramus of the lateral fissure*
7	F.C.L.r.retroC.tr	*Retro central transverse ramus of the lateral fissure*	8	S.Pe.C.median	*Median pre‐central sulcus*
9	S.Pe.C.marginal	*Marginal pre‐central sulcus*	10	S.Pe.C.sup	*Superior pre‐central sulcus*
11	S.Pe.C.inter	*Intermediate pre‐central sulcus*	12	S.Pe.C.inf	*Inferior pre‐central sulcus*
13	S.F.median	*Median frontal sulcus*	14	S.F.sup	*Superior frontal sulcus*
15	S.F.inter	*Intermediate frontal sulcus*	16	S.F.inf	*Inferior frontal sulcus*
17	S.F.inf.ant	*Anterior inferior frontal sulcus*	18	S.F.marginal	*Marginal frontal sulcus*
19	S.F.orbitaire	*Orbital frontal sulcus*	20	S.Or	*Orbital sulcus*
21	S.Po.C.sup	*Superior post‐central sulcus*	22	S.GSM	*Sulcus of the supramarginal gyrus*
23	F.I.P.	*Intraparietal sulcus*	24	F.I.P.Po.C.inf	*Inferior post‐central intraparietal sulcus*
25	F.I.P.r.int.1	*Primary intermadiate ramus of the intraparietal sulcus*	26	F.I.P.r.int.2	*Secondary intermadiate ramus of the intraparietal sulcus*
27	S.O.p	*Occipito polar sulcus*	28	S.T.pol	*Polar temporal sulcus*
29	S.T.s.	*Superior temporal sulcus*	30	S.T.s.ter.asc.ant	*Anterior terminal ascending branch of the superior temporal sulcus*
31	S.T.s.ter.asc.post	*Posterior terminal ascending branch of the superior temporal sulcus*	**32**	S.T.i.ant	*Anterior inferior temporal sulcus*
33	S.T.i.post	*Posterior inferior temporal sulcus*			
*Other sulci that might be found*					
34	F.C.M.post	*Calloso‐marginal posterior fissure*	35	S.p.C	*Paracentral sulcus*
36	S.Pa.int	*Internal parietal sulcus*	37	F.P.O	*Parieto‐occipital fissure*
38	S.Cu	*Cuneal sulcus*	39	S.Pa.sup	*Superior parietal sulcus*
40	S.Pa.t	*Transverse parietal sulcus*	41	S.O.T.lat.post	*Posterior occipito‐temporal lateral sulcus*
42	Occipital	*Occipital*			

The different sulci of the occipital lobes were not included in the list sent to the researchers due to their high complexity and inter‐subject variability. However, some researchers included some of them in their labelling and drawings. There were variations in the features that were labelled and in the terms used (e.g. Figure S3). Therefore, they were considered in the final analysis.

Some of the proposed sulci were merged in order to facilitate the analysis due to the high inter‐subject variability of the folding patterns in living humans. The sulci merged for the analysis are:

1. Central sulcus (S.C) and central Sylvian sulcus (S.C.sylvian) ⇒ Considered together as central sulcus (S.C).
2. Median pre‐central sulcus (S.Pe.C.median), marginal pre‐central sulcus (S.Pe.C.marginal), superior pre‐central sulcus (S.Pe.C.sup), intermediate pre‐central sulcus (S.Pe.C.inter) and inferior pre‐central sulcus (S.Pe.C.inf) ⇒ Considered as pre‐central sulcus (S.Pe.C).
3. Retro central transverse ramus of the lateral fissure (F.C.L.r.retroC.tr) and inferior post‐central intraparietal sulcus (F.I.P.Po.C.inf) ⇒ Considered as F.I.P.Po.C.inf.

### 2.3 Analysis of the data

#### 2.3.1 Dice score

**FIGURE 4:**
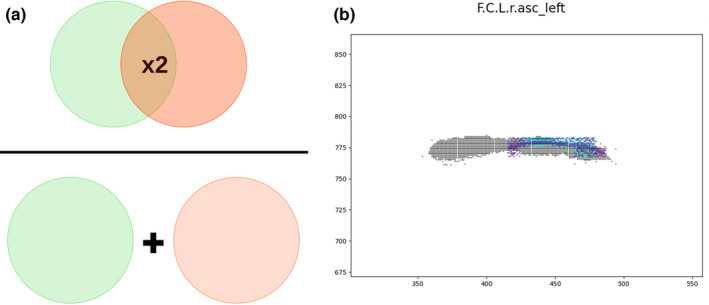
Dice index. (a) Schematic representation of the Dice index (Sørensen, 1948). (b) Example of the intersection of pixels using the Dice index when comparing the actual sulcus (F.C.L.r.asc, in black) and two manually designed sulci by researchers (in colours light blue and violet).

The Dice score, also known as Sørensen–Dice index or Sørensen coefficient, is a statistic used to quantify how similar the objects of two samples are. In image processing, this index can be used to compare the pixel‐wise agreement between a segmentation and its corresponding ground truth. As described in Equation 1, and illustrated in Figure 4a, this index corresponds to 2 times the size of the overlap in pixels of two segmented images (a and b) divided by the total number of pixels in both of them. The value for the Dice score fluctuates between 0 and 1, where 0 indicates no spatial overlap between two sets of binary segmented images and 1 indicates complete overlap. In our case, we will use it in a similar manner to compare pixel by pixel the similarity between the manually designed sulci with their ground truth (see Figure 4b).

(1) $$\text{Dice Score}=\frac{2*\left|A\cap B\right|}{\left|A\right|+\left|B\right|}$$

As this index compares the overlap between the manually drawn sulci on the endocast and the ground truth visible on the brain, the results obtained will be non‐zero only if there is any overlap between them. In other words, this index will work well where the drawn sulcus matches entirely, or at least in a large proportion, the ground truth (i.e. here the information directly extracted on the brain). However, this index will not be informative in cases where the drawn sulci do not overlap with the reference sulci, even if a manually drawn sulcus is very close in location and shape to the reference sulcus. This problem is even more relevant considering that the drawn sulcal lines differ in width between one researcher and another, being more difficult for a thin line to match the reference sulci in a big extent than for a thick line.

**FIGURE 5:**
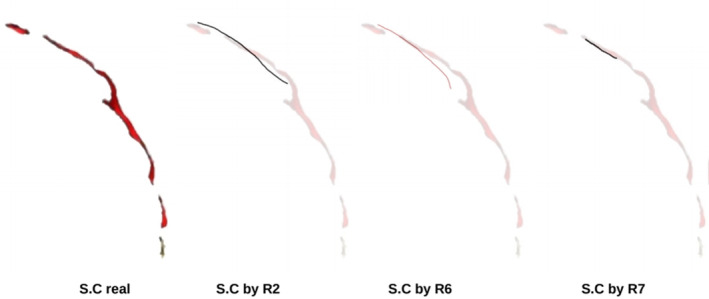
Comparison between the central sulcus ground truth observed on the brain (S.C real) and the manual drawings of it made by three researchers. The manual designs vary in shape and length and, even when there is an overlap between the drawings and the real sulcus, none of them approaches its real length.

As an example, Figure 5 shows the central sulcus as visible on the brain and the manual drawings of it made by three researchers on the endocast. The manual drawings vary in shape and length even when there is an overlap between the manually drawn sulcus and the real sulcus. In some cases, part of the drawing follows the shape pattern of the real sulcus, but though they are very close, they overlap little. In those cases, the Dice index is very low and does not accurately quantify the observed similarity. For these reasons, we have incorporated a second similarity index that more appropriately considers the shape and the proximity of the drawings to the reality.

#### 2.3.2 Mean minimum Euclidean distance

The idea of this similarity measure originates from the similarity distances used for the comparison of streamlines or fibres when creating white matter bundle atlases from tractography (Corouge et al., 2004; Guevara et al., 2011; Maddah et al., 2008; Mai et al., 2012). Usually, a pairwise distance metric based on Euclidean distance (see Equation 2) is used for a point‐to‐point comparison between streamlines. These measures capture the similarity in shape and the spatial proximity between the streamlines. In our case, instead of a point‐by‐point comparison between 3D coordinates, we carried out a 2D comparison pixel by pixel, considering only the coordinates of the pixels that belong to a sulcus (pixels with value different from 0). For this purpose, we applied the Euclidean norm as the square root of the sum of squares of the differences between corresponding indices of the 2D entries (pixels) (see Equation 2).

**FIGURE 6:**
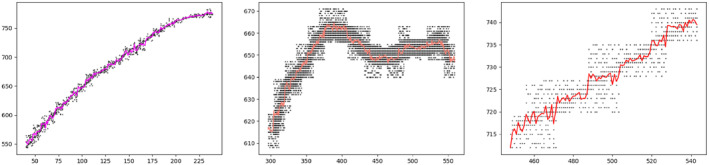
Comparison between the centroid obtained for different sulci (left fuchsia: central sulcus, middle orange: intermediate frontal sulcus and right red: anterior inferior frontal sulcus) and their original representation by pixels (in black).

Not only to simplify the computation of the distance between the coordinates of the pixels making up the sulci and the visualization, but also to make a fair comparison between the manually drawn sulcus that differ in width from one researcher to the other, we compute a ‘centroid’ that represents each sulcus manually drawn. This was possible due to the simplicity of the drawings, where the sulci pixels can be easily represented as a single central streamline of pixels (Figure 6). Each centroid was obtained using AdaBoostRegressor from Scikit‐learn (Pedregosa et al., 2012). As the ground truth sulci are more complex in shape, we did not compute centroids and we used all the pixel's indices for comparison.

Let us consider two sulci, A (manually drawn) and B (extracted from the brain), with a as one of the coordinates of a pixel that belongs to the centroid A′ representing the sulcus A and *b* one of the coordinates of a pixel that belongs to the sulcus B. Then, the Euclidean distance between *a* and *b* is:

(2) $$\mathrm{dE}\left(a,b\right)=\sqrt{{\left(a_{x}-b_{x}\right)}^{2}+{\left(a_{y}-b_{y}\right)}^{2}}$$

**FIGURE 7:**
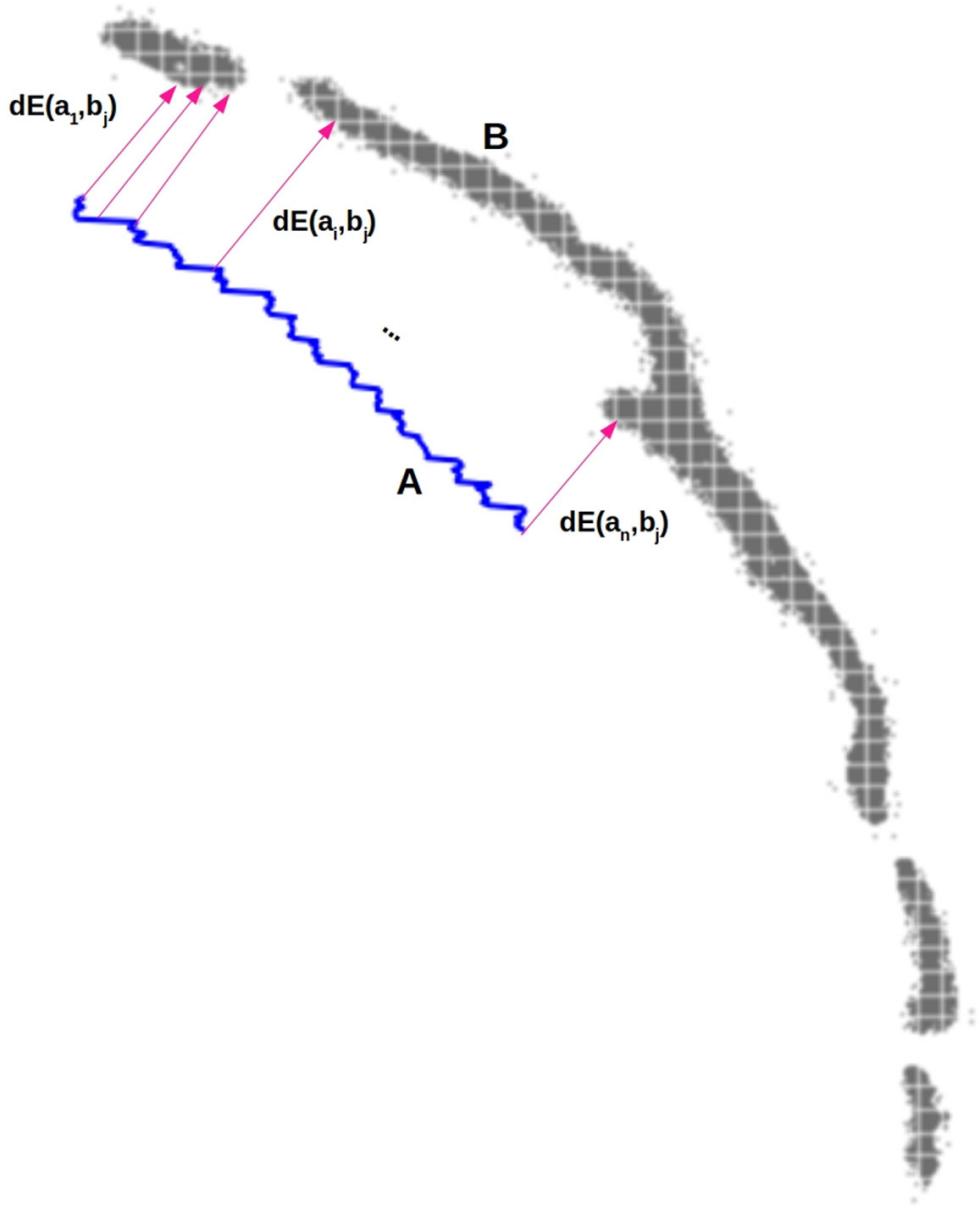
Computation of the MMED. For each a pixel in the centroid A, the closest b pixel in B is obtained by getting the minimum dE between the pixel a with all the b pixels in B. Once every a pixel in the centroid A has his closest b pixel, the average between all the minimums is obtained and that corresponds to the MMED.

For each pixel in the centroid *A*′, the Euclidean distance to every pixel making up *B* is computed. Then, the minimum distance is obtained, indicating what is the closest pixel in *B* for each pixel in *A*′. Finally, for each *A*′ centroid, the Mean Minimum Euclidean distance (MMED) to sulcus B will correspond to the average of all the minimum distances, as it is defined by Equation (3) and illustrated in Figure 7.

(3) $$\text{MMED}=\text{mean}\;\left(\min\left(\mathrm{dE}\left(A^{\prime },B\right)\right)\right)$$

(4) $$\mathrm{dE}\left(A^{\prime },B\right)=\mathrm{dE}\left(a_{i},b_{j}\right)$$

where *i* = 1,2,… *N* and *N* = number of points in the centroid of *A* (the sulcus drawn on the endocast); *j* = 1,2,… *M* and *M* = number of pixels in *B* (the sulcus defined on the brain); *a* = coordinates of a point in the centroid of sulcus *A*; *b* = coordinates of a pixel in the sulcus B.

#### 2.3.3 Similarity index for shape and spatial comparison (SISS)

Note that the MMED evaluates the similarity between both sulci identified respectively on the endocast and characterized from the evidence available on the brain only in the area of the sulcus drawn on the endocast. It does not give information about the relationship between the size of the sulci manually designed with respect to the entire length and shape of the sulci seen on the brain, as the Dice index does. Because of this, we think that both measures complement each other. Hence, we introduced the SISS, which combines the Dice score and the MMED.

In order to combine both indices, the MMED value had to be normalized. For this purpose, we established a threshold MMED value of 100. After analysing the data, we have realized that values over 100 correspond to misclassified sulcus that are spatially far away and differ in shape with respect to the ground truth sulcus. Thus, an MMED above this value implies that there is no similarity or proximity between the centroid and the sulci with which it is being compared. In the Dice score, a value close to 0 indicates no similarity, while a value close to 1 indicates high similarity. With the MMED, it is the opposite, the smaller the value, the greater the similarity, while a value close to 1 indicates the opposite. Because of this, the additive inverse of 1 is considered for the MMED normalized in order to have both indices working in the same manner and to be able to average them.

Finally, the MMED normalized (MMEDn) is defined as it is described in the Equation (4).

(4) $$\text{MMEDn}=\left\{\begin{array}{cc} 1-\left(\frac{\text{MMED}}{100}\right) & \text{if MMED}<100\\ 100 & \text{if MMED}>=100 \end{array}\right.$$

As a result, both indices operate in the same manner and can be averaged in the form of the SISS (see Equation 5). The SISS score varies between 0 and 1. The closer to 0, the less similarity and closeness between the ground truth sulci compared to the sulci drawn and the closer to 1 indicates greater similarity and closeness between them.

(5) $$\text{SISS}=\frac{\left(\text{Dice score}+\text{MMEDn}\right)}{2}$$

## 3 RESULTS

**FIGURE 8:**
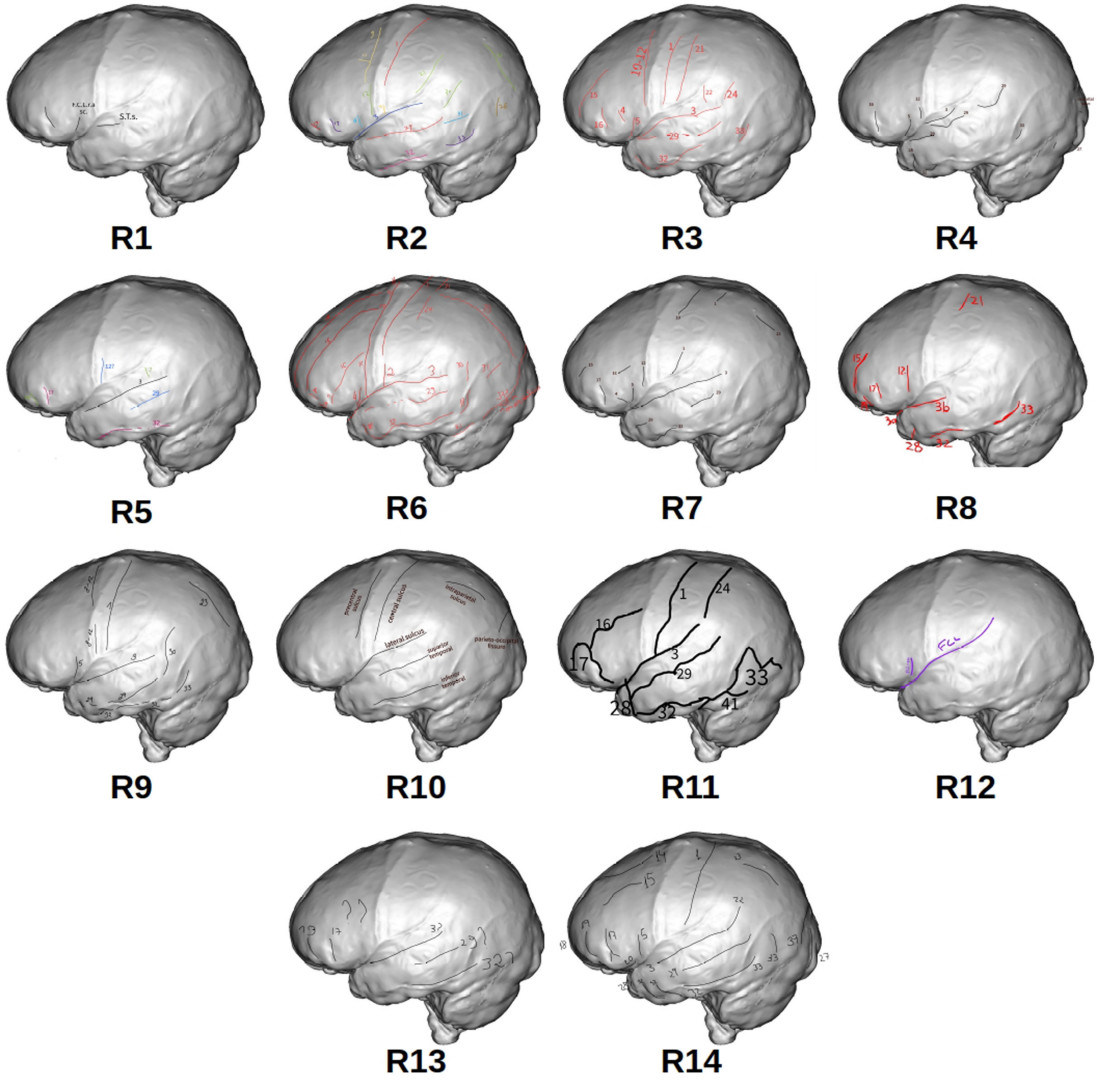
View of the left hemisphere drawings produced by each researcher participating in this study.

A summary of the resulting drawings and labelling produced by each researcher participating in the study can be observed in Figure 8. Only the left hemisphere view is displayed, but the results are similar for the remaining four views (Figures S2–S5). A preliminary qualitative assessment of these drawings shows the broad diversity of results in terms of how many sulci were identified by each researcher, their exact location and shape and how they were labelled. Each of the sulcal drawings made by each researcher in each view were isolated and saved as an independent image for subsequent analysis. All the sulci present in Tables 2 and 3 correspond to those that can be found in the image showing the sulci directly extracted from the brain for each view. Below we present the results separately for the two types of analyses, with and without corrected labels, that were carried out.

**TABLE 2:** Mean of the SISS index score for each sulcus considering all the views and average values for each sulcus on the left and right hemispheres.

Sulci	Anterior left	Anterior right	Superior left	Superior right	Posterior left	Posterior right	Lateral left	Lateral right	Average left side	Average right side
S.C	0.47	0.44	0.36	0.34	–	–	0.32	0.48	0.38	0.42
F.C.L	0.40	0.48	0.40	0.54	0.00	0.00	0.45	0.41	0.31	0.35
F.C.L.r.ant.	0.49	0.35	–	–	–	–	0.46	0.43	0.48	0.42
F.C.L.r.asc	0.54	–	–	–	–	–	0.55	0.57	0.54	0.57
F.C.L.r.diag	–	–	–	–	–	–	–	0.38	–	0.38
S.Pe.C	0.22	0.50	0.35	0.42	–	–	0.31	0.43	0.30	0.45
S.F.sup	0.05	0.08	0.42	0.45	–	–	0.32	0.38	0.26	0.30
S.F.inter	0.51	0.47	0.34	0.40	–	–	0.47	0.44	0.44	0.43
S.F.inf	0.42	0.45	0.45	0.48	–	–	0.40	0.35	0.42	0.42
S.F.inf.ant	0.52	0.51	0.57	0.28	–	–	0.54	0.46	0.54	0.43
S.F.marginal	0.45	0.33	0.37	–	–	–	0.36	0.00	0.40	0.17
S.F.orbitaire	0.33	0.31	–	–	–	–	0.43	0.26	0.38	0.28
S.Or	0.46	0.46	–	–	–	–	0.46	0.48	0.46	0.47
S.Po.C.sup	–	–	0.20	0.23	–	–	0.07	0.36	0.13	0.30
F.I.P	–	–	0.48	0.49	0.37	0.40	0.44	0.48	0.43	0.45
F.I.P.Po.C.inf	–	–	0.47	0.34	0.23	–	0.21	–	0.30	0.34
F.I.P.r.int.1	–	–	–	–	0.00	–	–	–	0.00	–
F.I.P.r.int.2	–	–	–	–	–	–	0.23	–	0.23	–
S.T.s	0.50	0.42	–	–	0.07	0.24	0.40	0.46	0.32	0.37
S.T.s.ter.asc.ant	–	–	–	–	0.17	0.07	0.30	0.37	0.23	0.22
S.T.s.ter.asc.post	–	–	–	–	0.23	0.31	0.26	0.22	0.25	0.27
S.T.pol	0.56	0.46	–	–	–	–	0.51	0.43	0.53	0.45
S.T.i.ant	0.28	0.49	–	–	0.43	0.30	0.37	0.39	0.36	0.39
S.T.i.post	–	–	–	–	0.47	0.46	0.44	0.48	0.46	0.47
S.Pa.int	–	–	–	–	–	–	–	–	–	–
S.Pa.sup	–	–	–	–	–	–	–	–	–	–
F.P.O	–	–	0.35	0.31	0.18	0.19	–	0.04	0.27	0.18
S.O.T.lat.post	–	–	0.46	0.41	0.40	0.32	0.32	–	0.40	0.37
Occipital	–	–	–	–	0.47	0.48	0.42	0.49	0.44	0.48

**TABLE 3:** Mean of the SISS index score for each sulci considering all the views and after correcting incorrect labels.

Sulci	Anterior left	Anterior right	Superior left	Superior right	Posterior left	Posterior right	Lateral left	Lateral right	Average left	Average right
S.C	0.54	0.57	0.48	0.42	–	–	0.46	0.50	0.49	0.50
F.C.L	0.51	0.49	0.42	0.55	–	–	0.50	0.48	0.48	0.50
F.C.L.r.ant.	0.49	–	–	–	–	–	0.48	0.53	0.49	0.53
F.C.L.r.asc	0.56	0.53	0.64	0.46	–	–	0.54	0.63	0.58	0.54
F.C.L.r.diag	0.51	–	–	–	–	–	–	0.41	0.51	0.41
S.Pe.C	0.46	0.50	0.53	0.48	–	–	0.49	0.49	0.49	0.49
S.F.sup	–	0.49	0.45	0.46	–	–	0.52	0.41	0.49	0.45
S.F.inter	0.51	0.52	0.49	0.48	–	–	0.47	0.50	0.49	0.50
S.F.inf	0.49	0.53	0.45	0.52	–	–	0.50	0.45	0.48	0.50
S.F.inf.ant	0.57	0.53	0.58	0.46	–	–	0.56	0.56	0.57	0.52
S.F.marginal	0.58	0.64	–	–	–	–	0.52	–	0.55	0.64
S.F.orbitaire	–	–	–	–	–	–	0.52	0.56	0.52	0.56
S.Or	0.50	0.51	–	–	–	–	0.46	0.48	0.48	0.49
S.Po.C.sup	–	–	0.48	–	–	–	–	0.63	0.48	0.63
F.I.P	–	–	0.49	0.49	0.49	0.48	0.44	0.49	0.47	0.49
F.I.P.Po.C.inf	–	–	0.47	–	–	–	0.45	–	0.46	–
F.I.P.r.int.1	–	–	–	–	–	–	–	–	–	–
F.I.P.r.int.2	–	–	–	–	–	–	–	–	–	–
S.T.s	0.52	0.51	–	–	–	0.44	0.50	0.47	0.51	0.47
S.T.s.ter.asc.ant	–	–	–	–	–	–	0.40	0.47	0.40	0.47
S.T.s.ter.asc.post	–	–	–	–	0.49	0.44	0.51	0.46	0.50	0.45
S.T.pol	0.54	0.55	–	–	–	–	0.52	0.54	0.53	0.55
S.T.i.ant	0.67	–	–	–	0.52	0.44	0.45	0.46	0.55	0.45
S.T.i.post	–	–	–	–	0.51	0.50	0.50	0.49	0.51	0.50
S.Pa.int	–	–	–	–	–	–	–	–	–	–
S.Pa.sup	–	–	–	–	–	–	–	–	–	–
F.P.O	–	–	–	–	0.52	0.47	–	–	0.52	0.47
S.O.T.lat.post	–	–	–	–	0.50	0.48	0.46	0.54	0.48	0.51
Occipital	–	–	0.53	0.51	0.50	0.49	0.52	0.49	0.52	0.50

### 3.1 Analysis with original manual labelling

**FIGURE 9:**
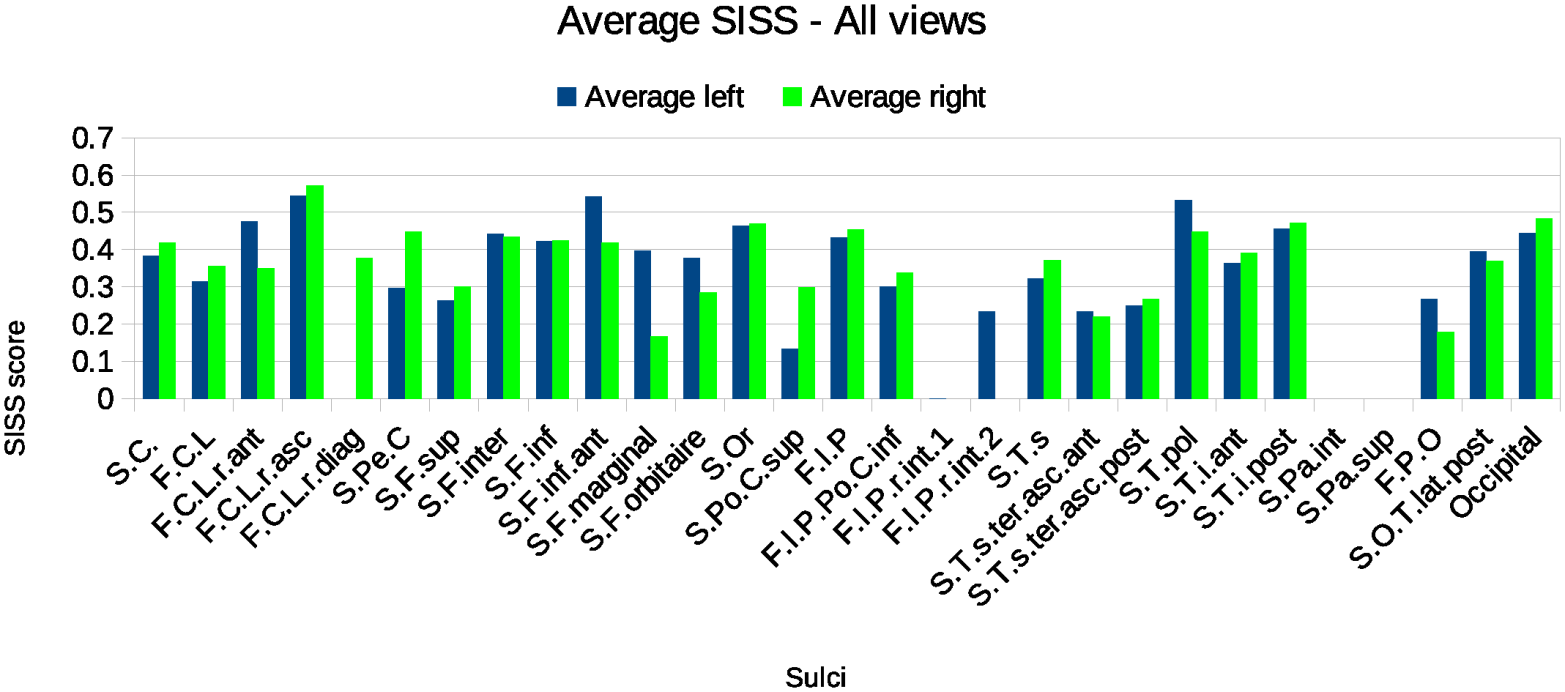
Average SISS score for every sulcus considering all the views, for the left and right hemispheres, with original manual labelling.

Table 2 details the mean SISS index scores for each sulcus considering all the views and all researchers, while Figure 9 shows the average SISS scores for each sulcus considering all the views separated for the left and right hemispheres. The underlying individual SISS scores for each of the sulcus that were identified by the researchers in each view are presented in Supplementary Tables S1. Sulci that were not present in the ground truth are not listed in the tables, despite being identified by some researchers. Moreover, Figure S6 illustrates the average SISS score for each sulcus in each view, as well as the number of researchers who identified the sulci in the same view.

With the original manual labelling, we observe 4.8% of the drawn sulci with MMED value that goes above 100. However, all those cases correspond to sulci that are far and with a different shape to the actual one, i.e. they are mislabelled sulci. As the MMED has a big influence in the SISS score, those outlier (and erroneous) values were having a big impact in the SISS. For this reason, the threshold of 100 was established, in order to have the final values between coherent ranges.

A first global and expected observation is that the orientation of the view has a great influence on the level of determination of sulci due to a combination of factors: the visibility of specific sulci and the available context information from other sulci in the different parts of the endocast vary greatly between the views. Independent of the view, there is a great variation in the level of determination of the sulci (Table 2 and Figure 9).

### 3.2 Analysis with the corrected labelling

**FIGURE 10:**
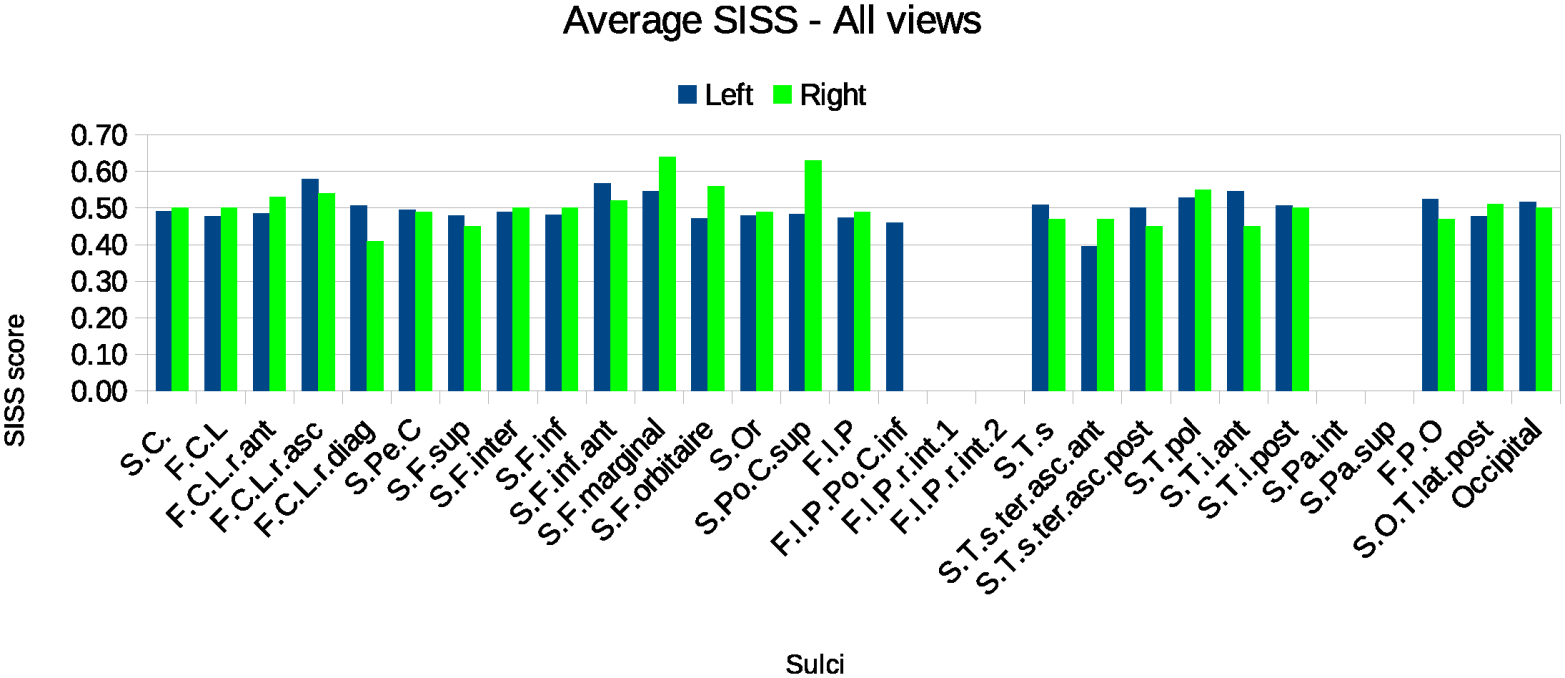
Average SISS score for every sulcus considering all the views, for the left and right hemispheres, with the corrected labelling.

Table 3 details the mean of the SISS index scores for each sulcus considering all the views, while Figure 10 shows the average SISS scores for every sulcus considering all the views separated for the left and right hemispheres. Tables S9–S16 present the underlying individual SISS scores for each of the sulci that were drawn by the researchers in each view. Moreover, Figure S7 illustrates the average SISS scores for every sulcus in each view as well as the number of researchers that identified the sulci in each view.

Here, all mislabelling in the raw data was corrected. It results first in that all MMED values for all the sulci are below 100, clearly demonstrating that values over 100 were an outlier due to wrong identification of sulci. Moreover, in some cases, the drawings could match two different sulci. In those cases, both sulci were considered, as the goal of these results is to analyse the relationship between the drawing and the actual underlying sulcal configuration, not the labelling. Those labels and drawings that did not represent any real sulci when comparing with the ground truth visible on the brain were not considered in this analysis. By doing so, results illustrate higher SISS scores for all the analysed sulci and a reduced variation between values (globally between 0.4 and 0.6).

**FIGURE 11:**
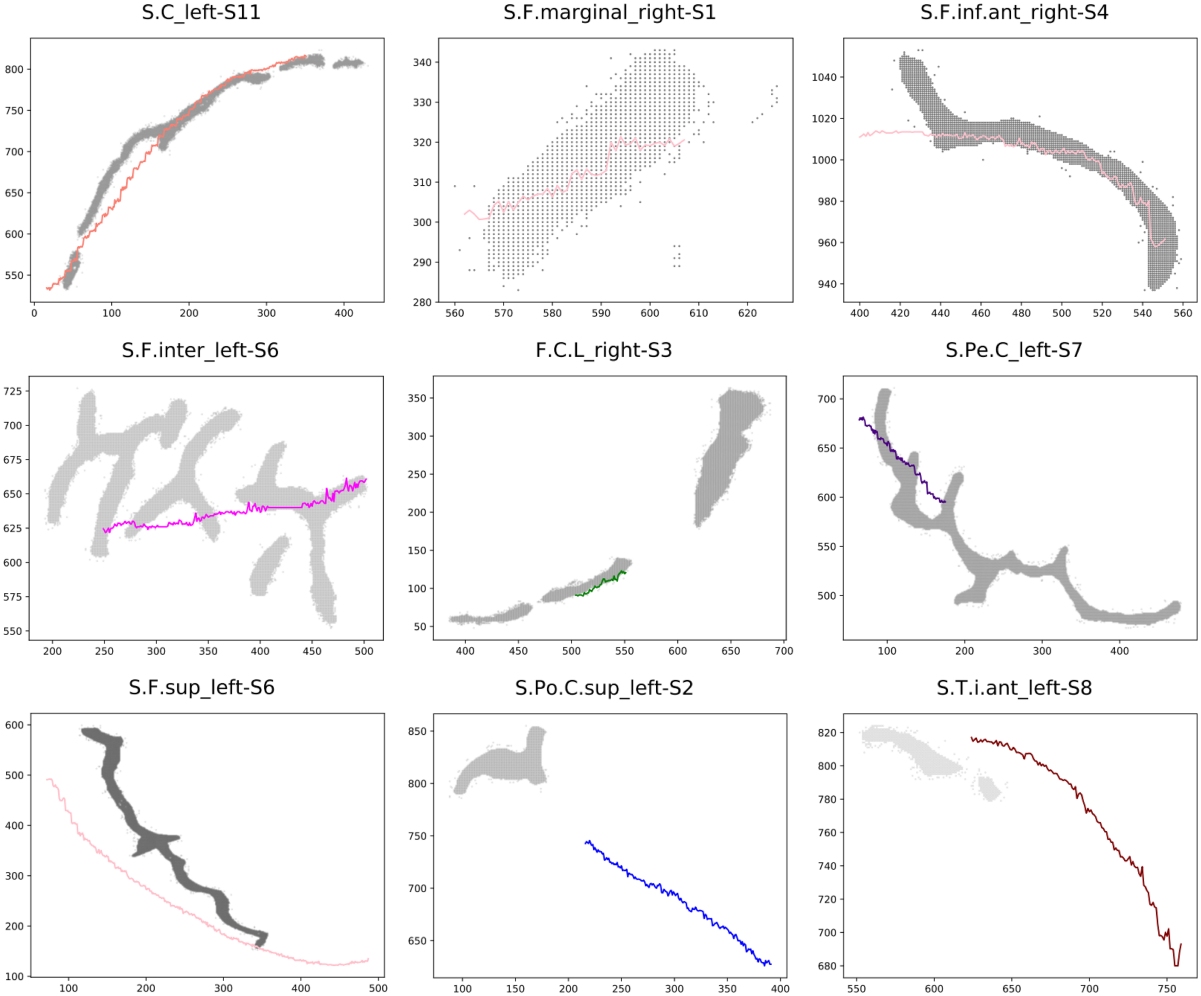
SISS scores for different sulci with comparison of the centroid of the sulcus drawn on the endocast (coloured lines) and the sulcus as it is visible on the brain (grey pixels). (a) Sulci with the highest SISS scores (above 0.6). From left to right: S.C left (SISS = 0.65), S.F.marginal left (SISS = 0.71), te S.F.inf.ant (SISS = 0.72). (b) Sulci with medium values of SISS score (between 0.4 and 0.5). From left to right: S.F.inter left (SISS = 0.51), F.C.L. right (SISS = 0.51), S.Pe.C left (SISS = 0.44). (c) Sulci with the lowest SISS scores (below 0.3). From left to right: S.F.sup left (SISS = 0.28), S.Po.C.sup left (SISS = 0.00), S.T.i.ant (SISS = 0.17).

Some sulci with the largest SISS score obtained (above 0.6) are shown (Figure 11). The shapes of the drawn sulci are very similar to the real sulci and overlap to a large extent or are very close. None of the sulci in both analyses (with and without label correction) have a value over 0.75, as this would require an almost perfect drawing in the entire extent of a sulcus. Examples of sulci with medium SISS score levels (between 0.4 and 0.5) and of sulci with a very low score, below 0.3, are also shown (Figure 11).

Generally speaking, sulcal drawings with SISS scores above 0.5 tend to overlap in position and match the shape (not throughout the full extension of the sulcus, but to an extent) of the actual sulci as visible on the brain. Sulci below but very close to that value (between 0.4 and 0.5) may not directly overlap or match the shape of the reference sulci, but they tend to be located close or in the same spatial location. Sulci with SISS values below 0.40 correspond mostly to the ones with a wrong label and that are far from the reference sulci.

**FIGURE 12:**
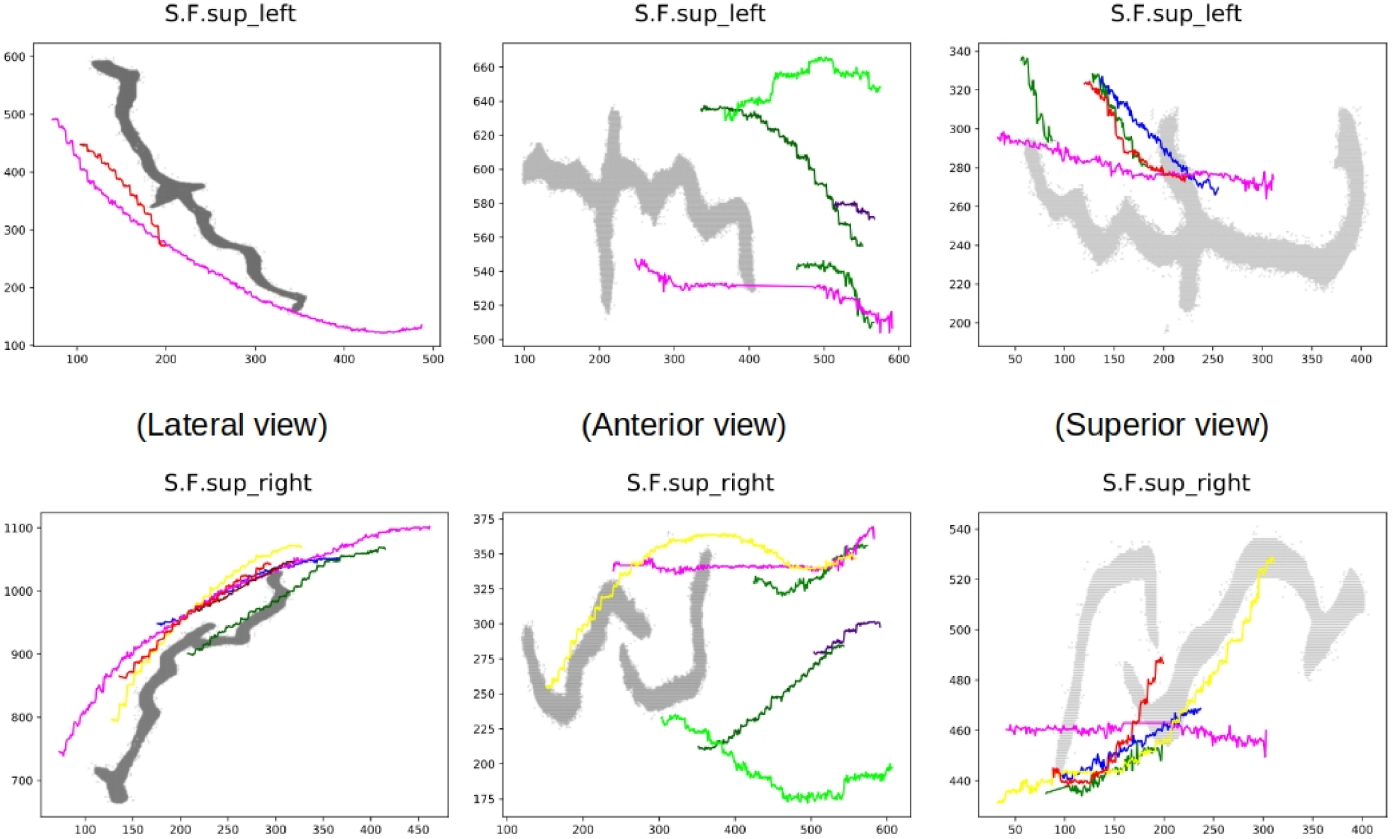
Superior frontal sulcus (S.F.sup). Comparison between the ground truth (grey) and the drawn sulci by the researchers in lateral, anterior and superior views (pink, red, yellow, dark green, green, light green, brown and blue).

Grooves located in the upper part of the endocast, mostly in the frontal and parietal regions and in particular, the superior frontal sulcus (S.F.sup) tends to be drawn in a rectilinear manner by many researchers (Figures 9, 10, 11). The superior frontal sulcus has been identified by a considerable number of researchers (around 40%) in both hemispheres in the different views. However, the average SISS obtained is very low for both hemispheres (below 0.3). As shown by Figure 12, in some views the drawings are close to the spatial position of the real sulcus (in grey). However, the shapes of the drawings are very far from the course of the sulci visible on the brain. After the correction of the labels, the number of researchers whose drawings were closer to the real sulci in at least one of the views was reduced drastically to two and with an average SISS score below 0.5, which indicates that they are closer in spatial position but very different in shape.

The case of other sulci in the upper part of the endocast is similar, but to a lesser extent: The superior post‐central sulcus (S.Po.C.sup) (high SISS score in one hemisphere but identified only by one researcher), the superior post‐central intraparietal superior sulcus (F.I.P.Po.C.inf) (similar to S.Po.C.sup) and the parieto‐occipital fissure (F.P.O), whose case is further explained below. The internal parietal sulcus (S.Pa.int) and the superior parietal sulcus (S.Pa.sup) were not identified by any researcher. The only sulcus of this area that was identified by a high number of researchers but that, even after label correction, obtained a SISS score below 0.5 was the F.I.P.

In relation to the difficulties linked to the correct identification of sulci in the upper part of the brain, we have two special but interesting cases: the well‐known central sulcus (S.C) and the different branches of the pre‐central sulcus (S.Pe.C).

**FIGURE 13:**
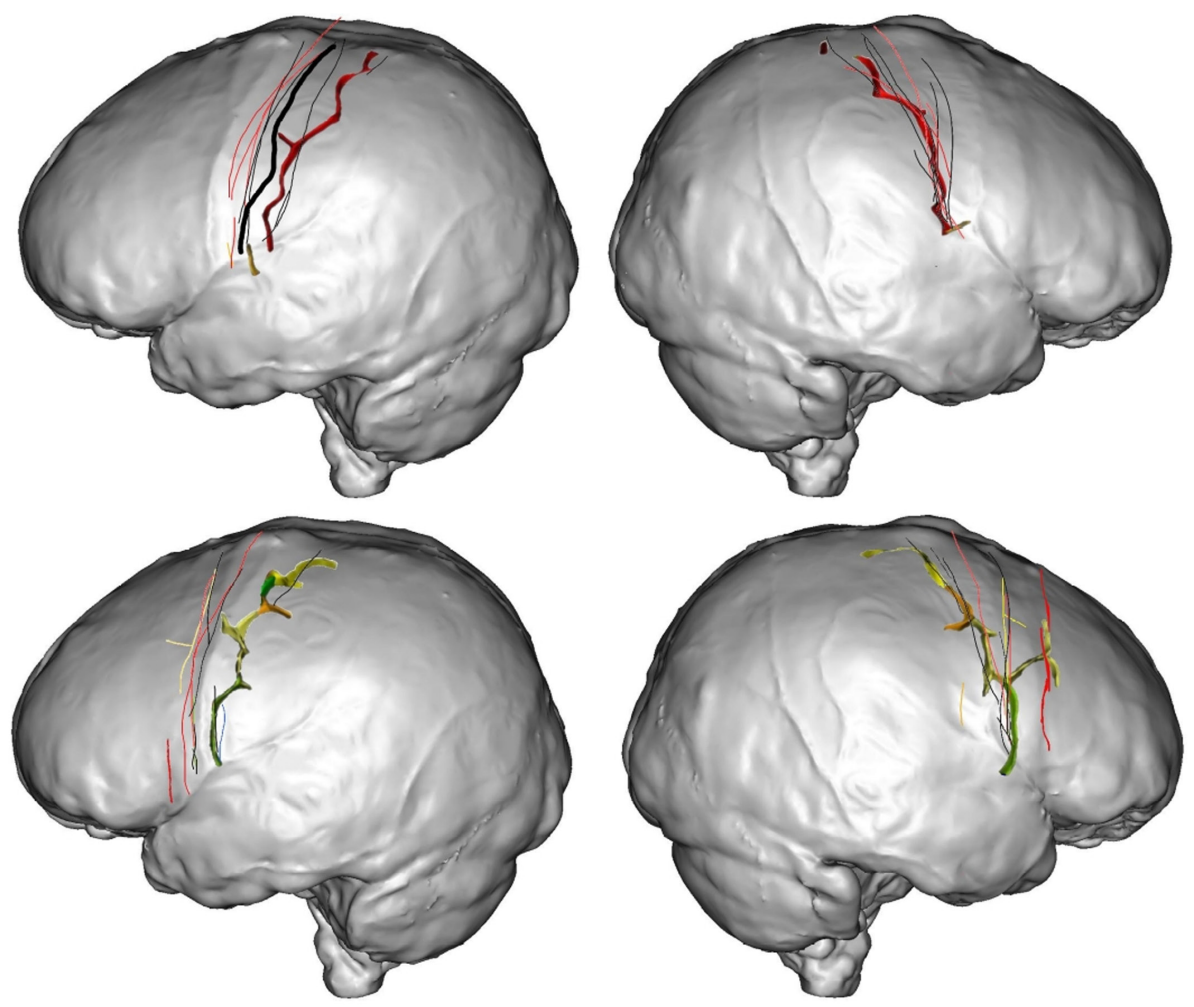
Central (top) and pre‐central (bottom) sulcus identification in both hemispheres. The drawings made by the researchers on the endocasts (straight lines) are superimposed with the ground truth, i.e. the real course of the sulci on the brain (central sulcus in red, different parts of the pre‐central sulcus in green, orange and yellow from bottom to top) in order to compare them.

The top part of Figure 13 shows a comparison between the real central sulcus (in red) and the drawings for the left and right hemisphere made by the researchers whom identified it in lateral view (eight researchers). While the SISS score is not extremely low, it is not particularly good for such a prominent and well‐known sulcus (below 0.5 in both hemispheres on average). The reason for this relatively low score is apparent when looking at the images: for the left hemisphere, only two researchers were relatively close to the origin of the sulcus, but the drawing deviates from the course of the reference sulcus in its superior region, near the branches of the pre‐central sulcus. The rest of the manually labelled central sulci are relatively far from the reference position, shifted towards the pre‐central region. Manual identification of the central sulcus in the right hemisphere is more accurate, but there is still substantial deviation and a tendency to draw the superior part of the sulcus in an area that is anterior to its actual position.

When analysing the branches of the pre‐central sulcus (S.Pe.C) the situation is a little different. Those sulci also had a high rate of identification (9 out of 10 researchers identified them in lateral and superior views) with an intermediate‐low SISS score (below 0.5). The lower panel of Figure 13 shows a bias in the positioning of these sulci towards the anterior region of the endocast in the left hemisphere, as it happened with the central sulcus (S.C). On the right side, the situation is quite similar. Although there is a bias towards the anterior region, at least some researchers have located the lower branches in a position that is close to reality.

**FIGURE 14:**
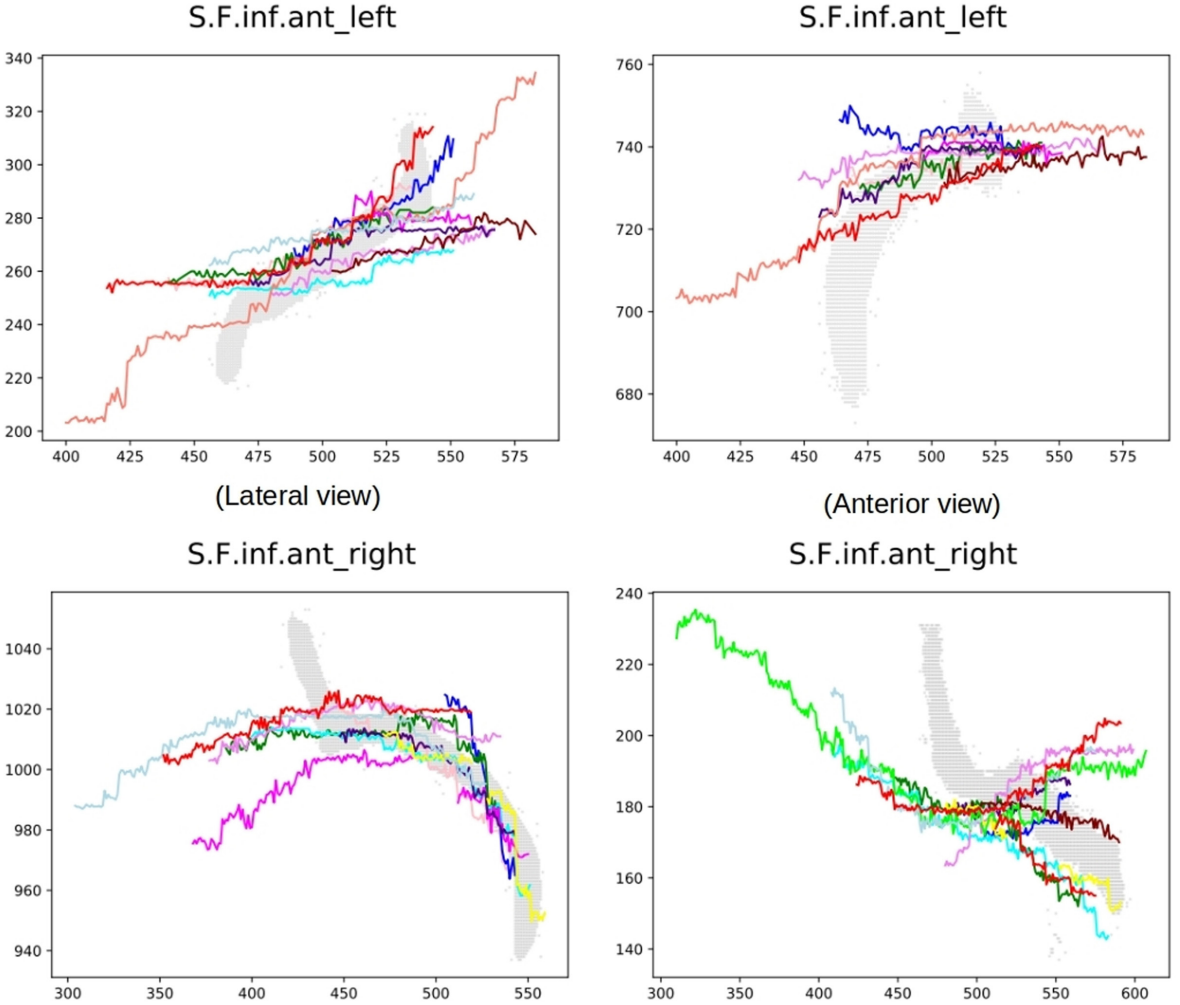
Anterior inferior frontal sulcus (S.F.inf.ant). Comparison between the ground truth and the drawn sulci by researchers in different views.

At the extreme opposite, the inferior anterior frontal sulcus (S.F.ins.ant) is the sulcus with the highest SISS score before and after label correction (over 0.52 on average in both hemispheres). This sulcus was also identified by more than 60% of the researchers in the two views where it is clearly visible. Figure 14 shows the comparison between the ground truth and the drawings made by the researchers after label correction. It can be noticed that most of the drawings overlap to a large extent with the real sulcus in the different views. In some cases, such as in the lateral right view, they clearly follow the shape of the real sulcus. This is one of the sulci that can be easily identified by looking at the marks found on the endocast.

The inferior frontal sulcus (S.F.inf) and the intermediate frontal sulcus (S.F.inter) are close to the previous one and they were commonly mislabelled. Both sulci also present an intermediate SISS score (around 0.5 for both hemispheres) and less researchers identified them (below 50%). Something similar occurs with the marginal frontal sulcus (S.F.marginal), which has a high SISS average score (above 0.55 for both hemispheres), but was identified by less than 40% of researchers.

Another sulcus with a very high SISS (over 0.54 on average for both hemispheres) is the ascending ramus of the lateral fissure (F.C.L.r.asc). This sulcus was also recognized by approximately 50% of the researchers in at least one view. The anterior branch close to it, the ascending ramus of the lateral fissure (F.C.L.r.asc), also shows a relatively good SISS score (around 0.5 on average for both hemispheres), but it was identified by fewer researchers. The third branch, the diagonal ramus of the lateral fissure (F.C.L.r.diag) has a SISS score of 0.5 in one hemisphere, but it was identified only by two researchers in two different views.

In the temporal region, the polar temporal sulcus (S.T.pol) and posterior inferior temporal sulcus (S.T.i.post) present a SISS score around 0.5 for both hemispheres, with approximately 40% of researchers identifying them. The anterior inferior temporal sulcus (S.T.i.ant) has a similar SISS score only for the left hemisphere. In the posterior region of the brain, the occipital sulcus and the posterior occipito‐temporal lateral sulcus (S.O.T.lat.post) have a similar average SISS score, around 0.5. The number of researchers identifying both is higher for the occipital sulcus (around 40% in the best case). The parieto‐occipital fissure (F.P.O) has also a high SISS value in one of the hemispheres, but it was only identified by one researcher.

**FIGURE 15:**
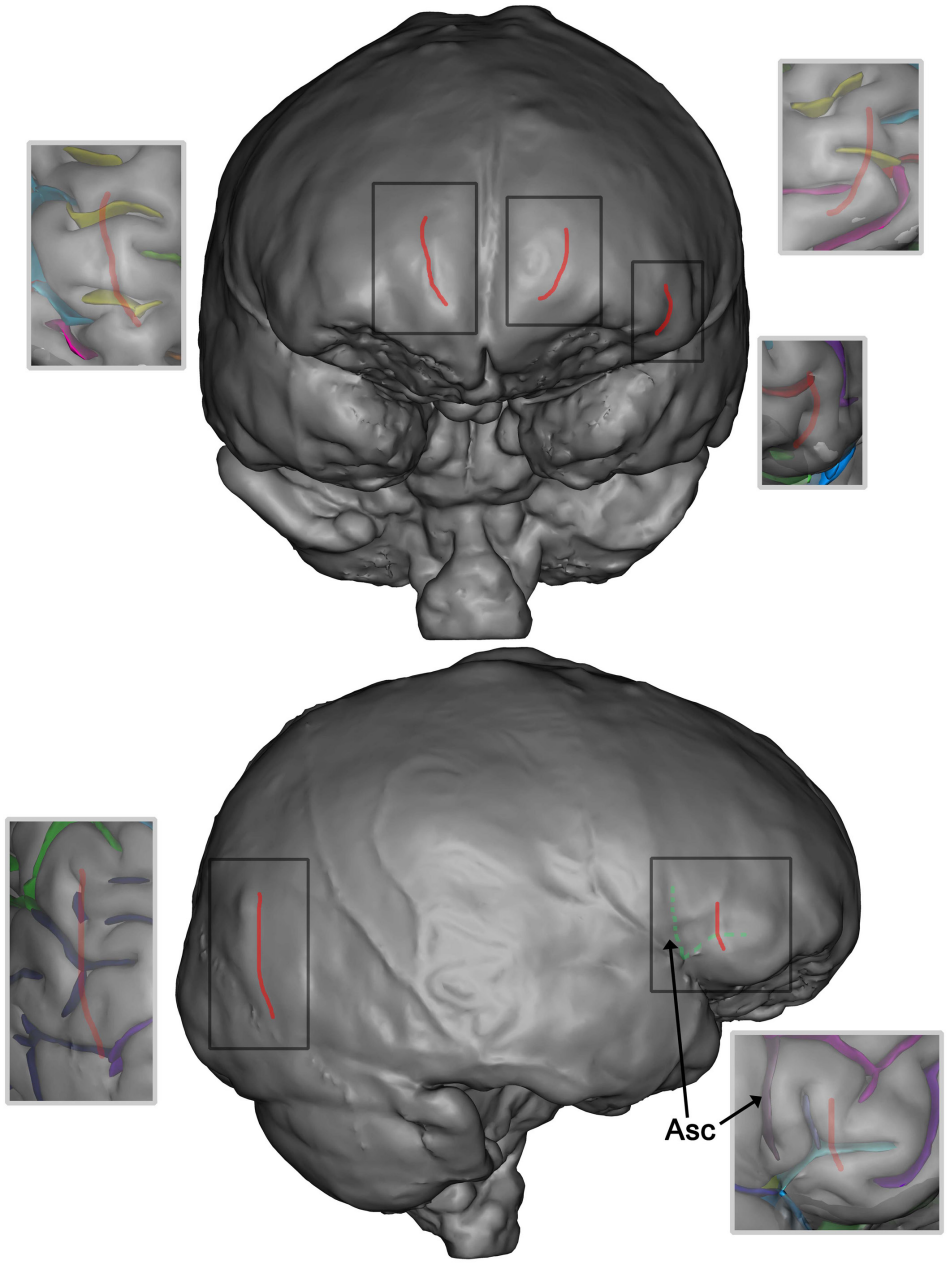
Examples of some traces in the endocast that were labelled as imprints of sulci (in red) by the observers but that are not as revealed by the morphology of the brain. The green dotted lines on the endocast in lateral view show the position show the true position of the ascending and horizontal rami of the lateral sulcus (the small light purple depression on the brain visible posteriorly the red line on the endocast might be the triangularis sulcus). On the occipital lobe, this vertical imprint has been frequently labelled by the experts but it does not correspond to any brain sulci as observed from the anatomy of the brain.

Another interesting observation was the finding of marks on the endocast that clearly seemed to be sulcal imprints. However, by contrast with the underlying sulcal anatomy, they show no correspondence with actual sulci, and the origin of those marks is unclear. Some examples of such cases can be found in Figure 15, where the manual identification made by researchers is compared with the actual configuration. In all these cases, multiple researchers participating in the study misclassified these marks as sulci. This made us wonder whether this phenomenon is related to the imaging used to create the endocast (MRI image instead of CT) or whether this is real anatomical variation found in other individuals and/or brain regions.

**FIGURE 16:**
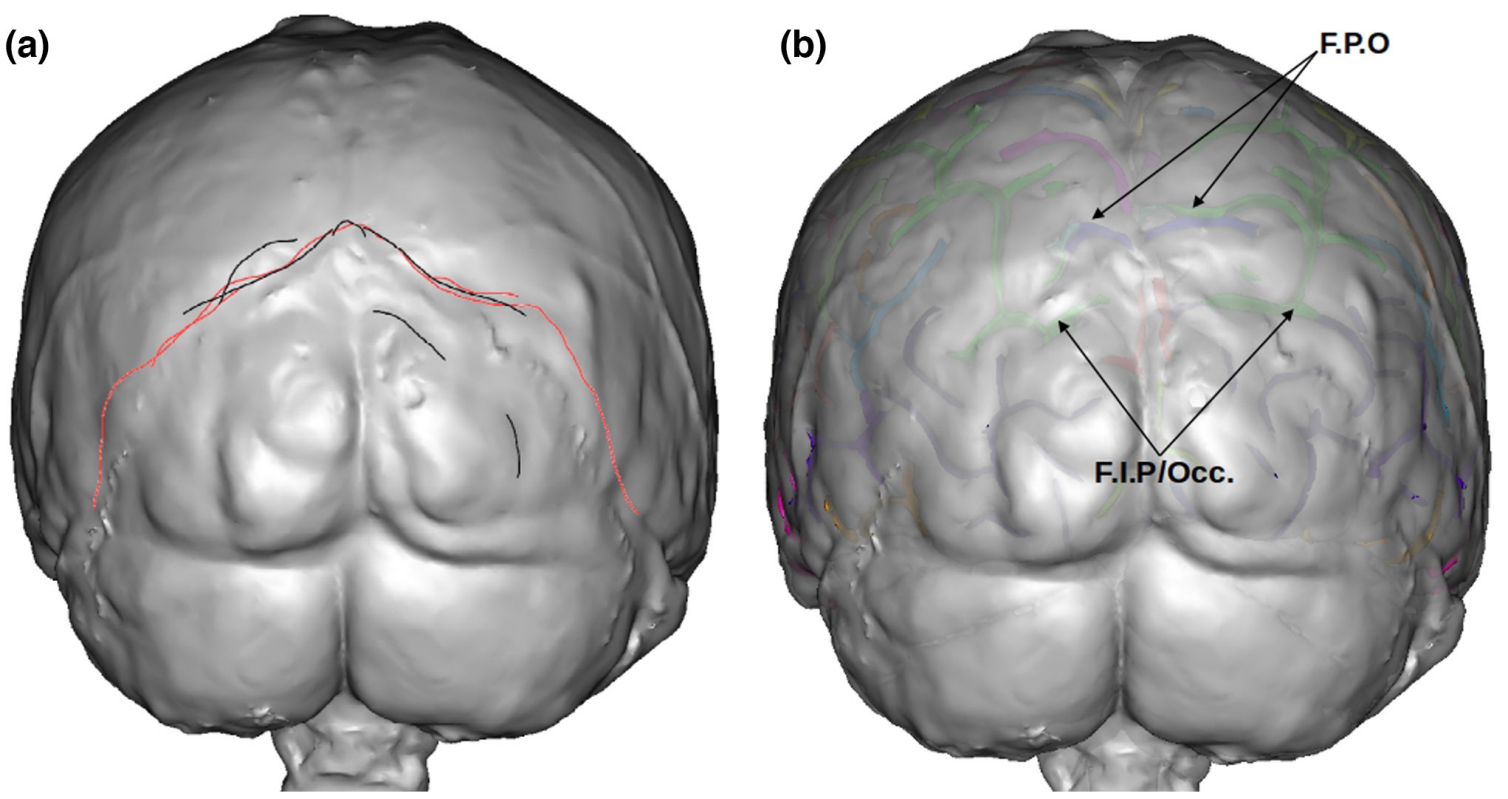
The parieto‐occipital fissure (F.P.O in blue on b) was misidentified by most researchers. While some placed its origin relatively close to the real position, the drawn shapes representing it (a, in black and in red) tend to follow the path of the occipital sulcus located below, as can be observed in b where the actual sulci are displayed. Some of the drawings also follow the depressions above the lambdoid suture that are not directly related to the course of brain sulci.

In most cases, the origin of these marks is unknown. However, we have noticed that, along all the extensions of the Lambdoid suture, there are grooves that seem to be sulcal imprints, but they are not (see Figure 16).

One of the sulci in this endocranial region that was frequently misidentified is the parieto‐occipital fissure (F.P.O). Figure 16a shows all the drawings corresponding to the F.P.O made by all the researchers who identified this sulcus, for the left and right hemispheres. While very few placed its origin relatively close to the real position, the drawings tend to follow other sulcal paths located below [the occipital or also a part of the intraparietal sulcus (F.I.P)], or some of the grooves around the Lambdoid suture that do not correspond to sulcal imprints. As mentioned earlier, we suggest caution when working with the parieto‐occipital fissure (F.P.O). While five researchers attempted to identify it in at least one of the views, only one of them obtained a SISS score above 0.5 and only for the left hemisphere.

## 4 DISCUSSION

The aim of this study was to quantify how accurately and consistently researchers identify gyri (or convolutions) and sulci of the human brain on a corresponding endocast.

Despite the relevance of the results presented in this study to the field of palaeoneurology, a number of limitations need to be discussed. First, the observations made in the context of this research were based on the visual assessment of pictures depicting the five anatomical views of an endocast, while in many cases researchers would work directly from a physical cast of the endocranial imprints of the brain. This issue is, nevertheless, balanced by the fact that the 3D model from which the views were captured was also made available to the researchers to let them fully and freely evaluate the features. The use of 3D models to collect morphological data has been fast developing in palaeoanthropology and palaeoneurology and may be seen as the development of a new standard in morphological studies.

Another issue concerns the fact that the results and observations were obtained from a single adult individual, leaving aside morphological variation that exists between specimens from the same species. Although our results are preliminary and we hope they will eventually be validated on a larger sample, our experimental design accurately reproduces the conditions and limitations of classic palaeoneurological research. Palaeoneurologists are usually presented with a single endocast that they need to assess based on comparisons with other hominin endocasts, using actual brain anatomy in humans and the great apes (usually chimpanzees and/or bonobos), in the context of their general neuroanatomical knowledge (e.g. Cofran et al., 2023), while the patterns of endocranial variation that are found within the same population or even within the same species are generally overlooked. Moreover, it is documented that smaller brained primate species and younger individuals tend to show more details than larger brain species and adults respectively (e.g. Falk, 2014). We have used here an adult volunteer. It is worth noting that the global endocranial cast shape reflects the one of the brain at the time of normal brain growth completion. The endocast is not expected to vary to a great extent during adulthood. In this context, it is logical to analyse a young adult to be able to compare the brain with the endocast as the brain undergoes more marked changes in adulthood and during later life. Therefore, although not entirely reflective of the potential variation in accuracy associated with endocranial assessment, our results do show how (in)accurate these identifications can be. This perspective is particularly important and the results and observations of this study provide information that will help researchers make more reliable inferences in future palaeoneurological studies.

For instance, many studies compare the external surface of the endocast as a whole (e.g. Beaudet et al., 2021; Neubauer et al., 2018; Zollikofer et al., 2022), even though the shape of the Neandertal brain and details of gyrification remain unknown. Attempts at reconstructing *Homo neanderthalensis* brain shape have been made from the retrodeformation of a *H. sapiens* brain (Kochiyama et al., 2018), but the variation in brain structure that is documented between *H. sapiens* and *H. neanderthalensis* was not considered (Balzeau et al., 2012). To ameliorate this kind of approach, it is important to improve our ability to describe the finest details of the brain from studying endocasts, especially when it comes to defining features that delimit different cerebral areas. This is crucial because recent research in neuroscience illustrates that comparative study of the brain sulci between primate species is challenging but has a high potential (Friedrich et al., 2021; Hopkins et al., 2022). Another recent study postulates that ‘tertiary sulci can likely inform the relationship between cortical folding and the evolution of human cognition in two main ways: emergence and quantification’ (Miller & Weiner, 2022, p. 737), which is undoubtedly true, but we first need to assess the accuracy and consistency of sulci identification on endocasts.

This is exactly what this study brings to palaeoneurology. Our most fundamental finding regarding accuracy and consistency of the observations is that there are clear differences in the results when comparing the non‐corrected labels with the corrected labels (Figures 9, 10, 11). This result, from several sulci, raises questions concerning the way researchers make their observations. Were the sulci really drawn on the different views considering and following the marks that can be observed on the surface of the endocast in the corresponding area? Or was there some tendency to draw the almost rectilinear representation of a given sulcus because of preconceptions based on the literature? In some cases, there seems to be a tendency to try to ‘find’ a sulcus, whose relative position is well‐known, following the shape it ‘usually’ has in the literature or where it is supposed to be according to the traits observed on a mean brain configuration. This is an expected bias. Moreover, we had given to the observers a nomenclature obtained for a mean brain with rectilinear sulci. The variability of brain sulci not only in humans (e.g. de Vareille et al., 2022; Eichert et al., 2021; Juch et al., 2005; Mangin et al., 2019) but also in great apes (e.g. Falk et al., 2018) is substantial. The same has been described for endocasts (de Jager et al., 2019, 2022). Therefore, trying to reconstruct a sulcus following the very general known shape/position in the literature or from a mean specimen may induce a bias when looking at an endocast and trying to follow the marks observed there.

This becomes even more important in analyses of sulci and gyri of extinct species for which no living reference is available. Atlas or literature representations of sulcal shape are in general limited. It is likely that models that reflect mean shape bias the identification of the complex course of the real sulci of one individual when looking at a specific hominin endocast. Moreover, this reflects more globally the subjective nature of sulcal labelling and the complexity of this task, even on real brains where all the sulci can be perfectly seen (e.g. Mellerio et al., 2016; Nowinski, 2022). This is in part not only due to the existence of multiple different nomenclature systems, but also because, in some cases, there are genuine disagreements on the identity of some sulci. Arguably, even the sulcal labelling produced by the software used here, Morphologist, could be debated. We name this labelling the ‘ground truth’. This referential was obtained from the automatic determination of the software and was validated by an expert (NLB). This complexity justifies why we have decided to simplify the nomenclature used by grouping several sulci, or parts of sulci, under the same name. For brain anatomy, using broader sulcal labelling (e.g. pre‐central sulcus as one single sulcus instead of pre‐central superior, pre‐central inferior) helps to minimize this difficulty. Indeed, a simplified nomenclature has to be used to describe endocasts in this study because the level of details visible on this brain proxy is lower than for the brain. The discrepancy between the results when comparing the non‐corrected labels with the corrected labels also highlights the necessity to cross‐check the observed information on endocasts.

Our results also bring important observations about the relative position of the sulci on the brain and the corresponding imprints on the endocast. We observe for the central sulcus (S.C) and the different branches of the pre‐central sulcus (S.Pe.C) a consistent anterior bias in their identification. It is possible that the acquisition method contributes to this bias in the position of the sulci/imprints between the brain and the endocranium. Indeed, MRIs are obtained in the supine position, the brain is thus held towards the back of the skull by gravity, in any case in a different position than when we stand erect (Fournier et al., 2011). The endocranial surface is not affected. The brain sulcus which moves from the bottom upwards can perhaps be found in a slightly more posterior position in connection with the position of acquisition of the images. Moreover, the localization of central sulcus in both hemispheres is often asymmetrical and the nature of this asymmetry is not standard for all individuals (Davatzikos & Bryan, 2002; Mangin et al., 2004). Here, there is indeed a discrepancy in the way the observers have located those two sulci on the left and right sides. The distance between the sulci on the endocast and the real position of the sulci on the brain is larger antero‐posteriorly on the left side compared to the contralateral side. This antero‐posterior shift could be explained by various factors and this aspect will deserve specific interest in the future and will probably have implications in palaeoanthropology.

Despite these questions and limitations, the description presented here about the relative projection of important brain sulci on the corresponding endocast is important for palaeoneurology. Additionally, when comparing the endocast with the real folding configuration for both hemispheres in the posterior region (Figure 16c,d), it can be noted that the grooves around the lambdoid suture might have a correspondence with sections of the occipital sulci and the intraparietal sulcus (F.I.P) in only two particular places. The rest of the observable marks seem to have no relation with the foldings, so special caution is advised when trying to identify sulcal paths around this suture, as this is an endocranial region with a long history of mislabelling and confusion (Falk, 2014). It is likely that the long‐standing debate about the position of the lunatus sulcus (e.g. Falk, 1980; Gunz et al., 2020; Holloway et al., 2004) will not be resolved until a similar approach to the one used here is considered to ascertain the position of the imprint seen on the endocast that is related to the sulcus on the brain on samples of great apes and humans. Finally, we observe that the transition between the parietal lobes and the occipital lobes, here expressed as the parieto‐occipital sulcus visible in the medial part of the brain, seems to be located on the analysed specimen just anteriorly to the lambda on the endocast (see also Bruner et al., 2015). Again, we will have to confirm this observation on a larger sample to better evaluate the possible correspondence of the position of the parieto‐occipital sulcus on an endocast.

**FIGURE 17:**
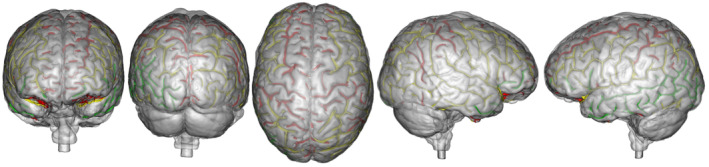
Representation of the areas/sulci that have a high, medium and low SISS score and rate of identification on the analysed specimen. The areas represented in green correspond to a high SISS score (over 0.5 on average) with over 20% of researchers identifying them in at least one view. The sulci/areas in yellow have an intermediate SISS score (between 0.4 and 0.5 on average) with over 20% of researchers identifying them in at least one view. The sulci/areas in red have a SISS score below 0.4 on average or less than 20% of the researchers identifying them in both hemispheres and in at least one view.

We also observe a general trend in our results, namely that the identification of sulci appears to be better in the lower part of the endocast compared to the upper part. Indeed, this seems to be because sulcal marks are more distinct in the lower than upper part of the endocast (perhaps due, at least partly, to the effects of gravity pulling the brain downward within the braincase). Figure 17 summarizes our results and shows the areas/sulci where sulcal identification is more or less reliable according to the SISS score and the number of researchers that identified the sulci after correction of the labels.

Finally, the results of this study may have specific implications for palaeoanthropology. For instance, a discrimination based on sulcal imprints has recently been proposed (Ponce de León et al., 2021) between early hominins, including *Australopithecus* and early *Homo erectus*, and all subsequent groups of *H. erectus* and more recent hominin species. The first group is characterized by a pre‐central sulcus in an anterior position in the region of the coronal suture, while in the second group the course of the sulcus extends posteriorly to the suture. In association with those two patterns, one predicts that the orbital cap would show a fronto‐orbital sulcus (S.F. orbitaire) in the primitive condition, while the pattern in this region in the more advanced *H. erectus* human‐like condition would include an ascending ramus and a horizontal ramus of the lateral sulcus. Our study brings some interesting information in this context. Many observers have located the pre‐central sulcus anterior to the coronal suture or crossing it on the analysed specimen (Figure 13) which could correspond to the ‘primitive’ condition described by Ponce de León and collaborators (Ponce de León et al., 2021). Moreover, the position of the ascending ramus and of the horizontal ramus on the endocast of this study (the dotted green lines on Figure 16) appears to be in the posterior area of the swelling corresponding to Broca's cap. It is likely that those sulci are in a more posterior position compared to the usual description on endocranial casts (e.g. Grimaud‐Hervé, 1997; Holloway et al., 2004). Moreover, the correspondence between the true position of those sulci and the imprints on the endocast is not very clear on this specimen. Finally, the relationship between the anterior or posterior position of the pre‐central sulcus and the configuration of the orbital cap was based on the differences seen between extant chimpanzees and humans. Future studies are needed to clarify these issues and to describe the fossil record of hominin endocasts with added caution, using high‐resolution material and thorough multiple observations by different palaeoneurologists. This aspect is generally acknowledged due to the complexity of the analysed material (e.g. Bruner & Beaudet, 2023; Ponce de León et al., 2021).

## 5 CONCLUSIONS

This study provides new information that can help palaeoneurologists better understand the relationship between sulci on brains and imprints on endocasts of *H. sapiens* and, by extrapolation, on endocasts from fossil hominins. We will continue to work with this perspective in mind. In the meantime, we suggest that palaeoanthropologists provide as much detailed information as possible in their morphological and taxonomical descriptions of endocasts. They should also be mindful of our finding that researchers often misinterpret a feature on an endocast as a linear propagation of a sulcus when it is not, perhaps because of expectations that stem from the literature.

It seems obvious that identifying sulci on endocasts based on their average appearances on brains in atlases has potential difficulties. The details of the potential relationship of the sulci/imprints between the brain and the endocast are not yet fully understood and need to be studied in more detail. The diversity of the cerebral folds on the brain is also not well‐known. Thus, one should probably only use the typical endocranial model to attempt to locate sulci on a particular endocranium, at least until an average endocranium model is established that overcomes the two noted problems (taking into account the inconsistency of reproduction of sulci on endocasts and a better appreciation of the brain–endocranium relationship). As we have shown, the a posteriori correction of the initial sulcal labellings of the imprints left by the brain on endocasts varies with their location within the cranium, i.e. the markings on the lower parts are more easily visible and better identified, in accordance with previous research (Connolly, 1950; de Jager et al., 2019; Dumoncel et al., 2021).

Endocranial description of fossil specimens should in the future consider the variation in position and shape of sulci in addition to using models of mean brain shape. Moreover, it is clear from this study that researchers can perceive sulcal imprints with reasonably high accuracy, but their correct identification and labelling remains a challenge, particularly when dealing with extinct species for which we lack direct knowledge of the brain. In this context, and in the interest of true engagement with Open Science, we recommend that publications include detailed views of endocasts in all orientations, with clear labelling of the observed traits, as well as public access to 3D models to allow for independent verification. In addition, sulcal labellings should be carried out independently by several researchers and then jointly discussed to resolve differences in observations of their positions, shapes (extents) and names. We hope to continue this work by studying a larger sample in order to find ways to improve the quality of identifying anatomical traits on brain endocasts.

## AUTHOR CONTRIBUTIONS

*Designed research*: Nicole Labra, Aurélien Mounier, and Antoine Balzeau. *Contributed analytical tools*: Mélanie Didier, Nicole Labra, Yann Leprince, Denis Rivière, and Jean François Mangin. *Collected samples*: Mélanie Didier, Emiliano Bruner, and Mathieu D. Santin. *Collected data*: Nicole Labra, Lou Albessard‐Ball, Amélie Beaudet, Douglas Broadfield, Emiliano Bruner, Kristian J. Carlson, Zachary Cofran, Dean Falk, Emmanuel Gilissen, Aida Gómez‐Robles, Simon Neubauer, Alannah Pearson, Carolin Röding, Yameng Zhang, and Antoine Balzeau. *Analysed/interpreted data*: Nicole Labra and Antoine Balzeau. *Wrote/revised the article*: Nicole Labra, Aurélien Mounier, Yann Leprince, Denis Rivière, Mélanie Didier, Emiliano Bruner, Mathieu D. Santin, Jean François Mangin, Andréa Filippo, Lou Albessard‐Ball, Amélie Beaudet, Douglas Broadfield, Emiliano Bruner, Kristian J. Carlson, Zachary Cofran, Dean Falk, Emmanuel Gilissen, Aida Gómez‐Robles, Simon Neubauer, Alannah Pearson, Carolin Röding, Yameng Zhang, and Antoine Balzeau.

## CONFLICT OF INTEREST STATEMENT

The authors declare that we they have no conflict of interest to report.

## Supporting information


Table S1.

## Data Availability

The 3D models of the endocast and of the brain are available on simple request to the corresponding author.

## Footnotes

^1^
Website: https://sketch.io/sketchpad/, last accessed on 12 October 2022 at 16:13 pm.

^2^

https://paleobrain.jimdofree.com last accessed on 12 October 2022 at 20:50 pm.

## References

[joa13966-bib-0001] Balzeau, A. , Grimaud‐Hervé, D. & Jacob, T. (2005) Internal cranial features of the Mojokerto child fossil (East Java, Indonesia). Journal of Human Evolution, 48, 535–553. 15927659 10.1016/j.jhevol.2005.01.002

[joa13966-bib-0002] Balzeau, A. , Holloway, R.L. & Grimaud‐Hervé, D. (2012) Variations and asymmetries in regional brain surface in the genus *Homo* . Journal of Human Evolution, 62(6), 696–706. Available from: 10.1016/j.jhevol.2012.03.007 22542169

[joa13966-bib-0003] Balzeau, A. & Mangin, J.F. (2021) Which synergies between paleoanthropology and brain imaging? Symmetry, 13, sym13101974.

[joa13966-bib-0004] Beaudet, A. , Holloway, R.L. & Benazzi, S. (2021) A comparative study of the endocasts of OH 5 and SK 1585: implications for the paleoneurology of eastern and southern African *Paranthropus* . Journal of Human Evolution, 156, 103010. Available from: 10.1016/j.jhevol.2021.103010 34020294

[joa13966-bib-0005] Bruner, E. (2019) Human paleoneurology: shaping cortical evolution in fossil hominids. The Journal of Comparative Neurology, 527, 1753–1765. 30520032 10.1002/cne.24591

[joa13966-bib-0006] Bruner, E. , Amano, H. , de la Cuétara, J.M. & Ogihara, N. (2015) The brain and the braincase: a spatial analysis on the midsagittal profile in adult humans. Journal of Anatomy, 227, 268–276. 26200138 10.1111/joa.12355 PMC4560561

[joa13966-bib-0007] Bruner, E. & Beaudet, A. (2023) The brain of Homo habilis: three decades of paleoneurology. Journal of Human Evolution, 174, 103281. 36455402 10.1016/j.jhevol.2022.103281

[joa13966-bib-0008] Cofran, Z. , Hurst, S. , Beaudet, A. & Zipfel, B. (2023) An overlooked Australopithecus brain endocast from Makapansgat, South Africa. Journal of Human Evolution, 178, 103346. 36958187 10.1016/j.jhevol.2023.103346

[joa13966-bib-0009] Cointepas, Y. , Mangin, J.‐F. , Garnero, L. , Poline, J.‐B. & Benali, H. (2001) BrainVISA: software platform for visualization and analysis of multi‐modality brain data. NeuroImage, 13, 98.

[joa13966-bib-0010] Connolly, J.C. (1950) External morphology of the primate brain. Springfield, IL: C. C. Thomas.

[joa13966-bib-0011] Corouge, I. , Gouttard, S. & Gerig, G. (2004) Towards a shape model of white matter fiber bundles using diffusion tensor MRI, in: 2004 2nd IEEE International Symposium on Biomedical Imaging: Macro to Nano (IEEE Cat No. 04EX821). In: Presented at the 2004 2nd IEEE International Symposium on Biomedical Imaging: Macro to Nano, IEEE, Arlington, VA, USA, pp. 344–347. Arlington, VA: Institute of Electrical and Electronics Engineers. Available from: 10.1109/ISBI.2004.1398545

[joa13966-bib-0012] Davatzikos, C. & Bryan, R.N. (2002) Morphometric analysis of cortical sulci using parametric ribbons: a study of the central sulcus. Journal of Computer Assisted Tomography, 26, 298–307. Available from: 10.1097/00004728-200203000-00024 11884791

[joa13966-bib-0013] de Jager, E.J. , Risser, L. , Mescam, M. , Fonta, C. & Beaudet, A. (2022) Sulci 3D mapping from human cranial endocasts: a powerful tool to study hominin brain evolution. Human Brain Mapping, 43, 4433–4443. Available from: 10.1002/hbm.25964 35661328 PMC9435008

[joa13966-bib-0014] de Jager, E.J. , van Schoor, A.N. , Hoffman, J.W. , Oettlé, A.C. , Fonta, C. , Mescam, M. et al. (2019) Sulcal pattern variation in extant human endocasts. Journal of Anatomy, 235(4), 803–810. Available from: 10.1111/joa.13030 31206664 PMC6742888

[joa13966-bib-0015] de Vareille, H. , Rivière, D. , Sun, Z.‐Y. , Fischer, C. , Leroy, F. , Neumane, S. et al. (2022) Shape variability of the central sulcus in the developing brain: a longitudinal descriptive and predictive study in preterm infants. NeuroImage, 251, 118837. 34965455 10.1016/j.neuroimage.2021.118837

[joa13966-bib-0016] Dumoncel, J. , Subsol, G. , Durrleman, S. , Bertrand, A. , de Jager, E. , Oettlé, A.C. et al. (2021) Are endocasts reliable proxies for brains? A 3D quantitative comparison of the extant human brain and endocast. Journal of Anatomy, 238, 480–488. Available from: 10.1111/joa.13318 32996582 PMC7812123

[joa13966-bib-0017] Eichert, N. , Watkins, K.E. , Mars, R.B. & Petrides, M. (2021) Morphological and functional variability in central and subcentral motor cortex of the human brain. Brain Structure & Function, 226, 263–279. Available from: 10.1007/s00429-020-02180-w 33355695 PMC7817568

[joa13966-bib-0018] Falk, D. (1980) Hominid brain evolution: the approach from paleoneurology. Yearbook of Physical Anthropology, 23, 93–107.

[joa13966-bib-0019] Falk, D. (2014) Interpreting sulci on hominin endocasts: old hypotheses and new findings. Frontiers in Human Neuroscience, 8, 134. 24822043 10.3389/fnhum.2014.00134 PMC4013485

[joa13966-bib-0020] Falk, D. , Zollikofer, C.P.E. , Ponce de León, M. , Semendeferi, K. , Alatorre Warren, J.L. & Hopkins, W.D. (2018) Identification of in vivo sulci on the external surface of eight adult chimpanzee brains: implications for interpreting early hominin endocasts. Brain, Behavior and Evolution, 91(1), 45–58. 29533941 10.1159/000487248

[joa13966-bib-0021] Fischer, C. , Operto, G. , Laguitton, S. , Perrot, M. , Denghien, I. & Rivière, D. (2012) Morphologist 2012: the new morphological pipeline of BrainVISA. In: Presented at the Proceedings of the 18th HBM Scientific Meeting, Neuroimage, Beijing, China. Minnessota: Organization for Human Brain Mapping.

[joa13966-bib-0022] Fournier, M. , Combès, B. , Roberts, N. , Braga, J. & Prima, S. (2011) Mapping the distance between the brain and the inner surface of the skull and their global asymmetries. In: Medical imaging 2011: image process, Vol. 7962, 79620Y. Bellingham, Washington: SPIE ‐ International Society for Optics and Photonics.

[joa13966-bib-0023] Friedrich, P. , Forkel, S.J. , Amiez, C. , Balsters, J.H. , Coulon, O. , Fan, L. et al. (2021) Imaging evolution of the primate brain: the next frontier? NeuroImage, 228, 117685. Available from: 10.1016/j.neuroimage.2020.117685 33359344 PMC7116589

[joa13966-bib-0024] Grimaud‐Hervé, D. (1997) L'évolution de l'encéphale chez Homo erectus et Homo sapiens: exemples de l'Asie et de l'Europe. Paris: CNRS éditions.

[joa13966-bib-0025] Guevara, P. , Poupon, C. , Rivière, D. , Cointepas, Y. , Descoteaux, M. , Thirion, B. et al. (2011) Robust clustering of massive tractography datasets. NeuroImage, 54, 1975–1993. Available from: 10.1016/j.neuroimage.2010.10.028 20965259

[joa13966-bib-0026] Gunz, P. , Neubauer, S. , Falk, D. , Tafforeau, P. , le Cabec, A. , Smith, T.M. et al. (2020) Australopithecus afarensis endocasts suggest ape‐like brain organization and prolonged brain growth. Science Advances, 6(14), eaaz4729gunz. Available from: 10.1126/sciadv.aaz4729 32270044 PMC7112758

[joa13966-bib-0027] Holloway, R.L. , Broadfield, D.C. & Yuan, M.S. (2004) Brain endocasts the paleoneurological evidence, Vol. 3. Hoboken, NJ: John Wiley and Sons, Inc.

[joa13966-bib-0028] Hopkins, W.D. , Sprung‐Much, T. , Amiez, C. , Procyk, E. , Petrides, M. , Schapiro, S.J. et al. (2022) A comprehensive analysis of variability in the sulci that define the inferior frontal gyrus in the chimpanzee (*Pan troglodytes*) brain. American Journal of Biological Anthropology, 179, 31–47.

[joa13966-bib-0029] Jerison, H.J. (1973) Evolution of the brain and intelligence. New York: Academic Press.

[joa13966-bib-0030] Juch, H. , Zimine, I. , Seghier, M.L. , Lazeyras, F. & Fase, J.H.D. (2005) Anatomical variability of the lateral frontal lobe surface: implication for intersubject variability in language neuroimaging. NeuroImage, 24, 504–514. Available from: 10.1016/j.neuroimage.2004.08.037 15627592

[joa13966-bib-0031] Kochiyama, T. , Ogihara, N. , Tanabe, H.C. , Kondo, O. , Amano, H. , Hasegawa, K. et al. (2018) Reconstructing the Neanderthal brain using computational anatomy. Scientific Reports, 8, 6296. 29700382 10.1038/s41598-018-24331-0 PMC5919901

[joa13966-bib-0032] Le Gros Clark, W.E. , Cooper, D.M. & Zuckerman, S. (1936) The endocranial cast of the chimpanzee. Journal of the Royal Anthropological Institute, 66, 249–268.

[joa13966-bib-0033] Maddah, M. , Grimson, W.E.L. , Warfield, S.K. & Wells, W.M. (2008) A unified framework for clustering and quantitative analysis of white matter fiber tracts. Medical Image Analysis, 12, 191–202. Available from: 10.1016/j.media.2007.10.003 18180197 PMC2615202

[joa13966-bib-0034] Mai, S.T. , Goebl, S. & Plant, C. (2012) A similarity model and segmentation algorithm for White matter fiber tracts. In: 2012 IEEE 12th International Conference on Data Mining. Presented at the 2012 IEEE 12th International Conference on Data Mining (ICDM). Brussels, Belgium: IEEE, pp. 1014–1019. Available from: 10.1109/ICDM.2012.95

[joa13966-bib-0035] Mangin, J.‐F. , Le Guen, Y. , Labra, N. , Grigis, A. , Frouin, V. , Guevara, M. et al. (2019) “Plis de passage” deserve a role in models of the cortical folding process. Brain Topography, 32, 1035–1048. Available from: 10.1007/s10548-019-00734-8 31583493 PMC6882753

[joa13966-bib-0036] Mangin, J.‐F. , Riviere, D. , Cachia, A. , Duchesnay, E. , Cointepas, Y. , Papadopoulos‐Orfanos, D. et al. (2004) Object‐based morphometry of the cerebral cortex. IEEE Transactions on Medical Imaging, 23, 968–982. Available from: 10.1109/TMI.2004.831204 15338731

[joa13966-bib-0037] Mellerio, C. , Lapointe, M.N. , Roca, P. , Charron, S. , Legrand, L. , Meder, J.F. et al. (2016) Identification of reliable Sulcal patterns of the human Rolandic region. Frontiers in Human Neuroscience, 17, 10–410. 10.3389/fnhum.2016.00410 PMC4987365 27582700

[joa13966-bib-0038] Miller, J.A. & Weiner, K.S. (2022) Unfolding the evolution of human cognition. Trends in Cognitive Sciences, 26(9), 735–737. 35909020 10.1016/j.tics.2022.06.008

[joa13966-bib-0039] Neubauer, S. , Hublin, J.J. & Gunz, P. (2018) The evolution of modern human brain shape. Science Advances, 4(1), eaao5961. Available from: 10.1126/sciadv.aao5961 29376123 PMC5783678

[joa13966-bib-0040] Nowinski, W.L. (2022) On the definition, construction, and presentation of the human cerebral sulci: a morphology‐based approach. Journal of Anatomy, 241(3), 789–808. 35638263 10.1111/joa.13695 PMC9358745

[joa13966-bib-0041] Ono, M. , Kubik, S. & Abernathey, C.A. (1990) Atlas of cerebral sulci. Verlag, Stuttgart: Thieme‐Stratton Corp.

[joa13966-bib-0042] Pedregosa, F. , Varoquaux, G. , Gramfort, A. , Michel, V. , Thirion, B. , Grisel, O. et al. (2012) Scikit‐learn: machine learning in Python. 10.48550/ARXIV.1201.0490

[joa13966-bib-0043] Perrot, M. , Rivière, D. & Mangin, J.‐F. (2011) Cortical sulci recognition and spatial normalization. Medical Image Analysis, 15, 529–550. Available from: 10.1016/j.media.2011.02.008 21441062

[joa13966-bib-0044] Ponce de León, M.S. , Bienvenu, T. , Marom, A. , Engel, S. , Tafforeau, P. , Alatorre Warren, J.L. et al. (2021) The primitive brain of early *Homo* . Science, 372(6538), 165–171. Available from: 10.1126/science.aaz0032 33833119

[joa13966-bib-0045] Robson, M.D. , Gatehouse, P.D. , Bydder, M. & Bydder, G.M. (2003) Magnetic resonance: an introduction to ultrashort TE (UTE) imaging. Journal of Computer Assisted Tomography, 27, 825–846. Available from: 10.1097/00004728-200311000-00001 14600447

[joa13966-bib-0046] Sørensen, T. (1948) A method of establishing groups of equal amplitude in plant sociology based on similarity of species content and ist application to analyses of the vegetation on Danish commons. Copenhagen: Det Kongelige Danske Videnskabernes Selskab. Munksgaard.

[joa13966-bib-0047] Zollikofer, P.E. , Bienvenu, T. , Suwa, G. , Asfaw, B. , White, T.D. & Ponce de León, M.S. (2022) Endocranial ontogeny and evolution on early *Homo sapiens*: the evidence from Herto, Ethiopia. Proceedings of the National Academy of Sciences, 119(32), e2123553119. 10.1073/pnas.2123553119 PMC9371682 35914174

