# Supplementary material for: What do brain endocasts tell us? A comparative analysis of the accuracy of sulcal identification by experts and perspectives in palaeoanthropology
Source: J Anat. 2023 Nov 7;244(2):274–96. doi: 10.1111/joa.13966 (PMC10780157; doi:10.1111/joa.13966)
Supplement: Supplementary file 1 — Table S1. [file JOA-244-274-s001.docx]

**Supplementary information**

Table S1. SISS index score for each sulci by each researcher in the anterior view, left hemisphere.

| **Sulci** | **R1** | **R2** | **R3** | **R4** | **R5** | **R6** | **R7** | **R8** | **R9** | **R10** | **R11** | **R12** | **R13** | **R14** | **Average** |
| --- | --- | --- | --- | --- | --- | --- | --- | --- | --- | --- | --- | --- | --- | --- | --- |
| **S.C** | - | 0.23 | - | - | - | 0.51 | 0.52 | - | 0.46 | - | 0.65 | - | - | - | 0.47 |
| **F.C.L** | - | 0.41 | 0.42 | - | - | 0.46 | 0.39 | 0.32 | - | - | - | - | - | - | 0.40 |
| **F.C.L.r.ant.** | - | - | 0.5 | - | - | - | - | - | - | - | - | - | - | - | 0.49 |
| **F.C.L.r.asc** | - | - | 0.54 | - | - | - | 0.00 | - | - | - | - | - | - | 0.55 | 0.54 |
| **F.C.L.r.diag** | - | - | - | - | - | - | - | - | - | - | - | - | - | - | - |
| **S.Pe.C** | - | - | 0.21 | - | - | 0.23 | 0.00 | - | 0.46 | - | - | - | - | 0.22 | 0.22 |
| **S.F.sup** | - | - | 0.00 | - | - | 0.15 | - | - | - | 0.11 | - | 0.00 | - | - | 0.05 |
| **S.F.inter** | 0.49 | 0.54 | 0.5 | - | - | 0.51 | 0.51 | - | - | - | 0.53 | - | - | - | 0.51 |
| **S.F.inf** | 0.35 | 0.52 | 0.44 | 0.32 | - | 0.46 | 0.63 | - | - | 0.44 | 0.29 | - | - | - | 0.42 |
| **S.F.inf.ant** | - | 0.56 | 0.29 | - | 0.48 | 0.51 | - | 0.48 | - | - | - |  | - | 0.66 | 0.52 |
| **S.F.marginal** | - | 0.33 | 0.53 | - | - | 0.13 | - | - | - | - | 0.56 | - | 0.58 | 0.59 | 0.45 |
| **S.F.orbitaire** | - | 0.41 | - | - | 0.44 | 0.33 | - | 0.14 | - | - | - | - | 0.25 | 0.41 | 0.33 |
| **S.Or** | 0.41 | 0.52 | - | - | - | - | - | - | - | - | - | - | - | - | 0.46 |
| **S.T.s** | - | 0.43 | 0.52 | - | - | 0.50 | - | - | - | - | - | - | - | 0.54 | 0.50 |
| **S.T.pol** | - | - | 0.53 | - | - | 0.54 | - | - | - | - | 0.53 | - | - | - | 0.56 |
| **S.T.i.ant** | - | - | 0.36 | - | - | - | 0.37 | 0.17 | - | - | - | - | - | 0.22 | 0.28 |

Table S2. SISS index score for each sulci by each researcher in the anterior view, right hemisphere.

| **Sulci** | **R1** | **R2** | **R3** | **R4** | **R5** | **R6** | **R7** | **R8** | **R9** | **R10** | **R11** | **R12** | **R13** | **R14** | **Average** |
| --- | --- | --- | --- | --- | --- | --- | --- | --- | --- | --- | --- | --- | --- | --- | --- |
| **S.C** | - | 0.34 | 0.16 | - | - | 0.54 | - | - | 0.55 | - | 0.62 | - | - | - | 0.44 |
| **F.C.L** | - | 0.45 | 0.51 | - | - | 0.51 | 0.44 | - | - | - | - | - | - | - | 0.48 |
| **F.C.L.r.ant.** | - | - | - | - | - | - | - | - | - | - | - | - | - | 0.35 | 0.40 |
| **F.C.L.r.asc** | - | - | - | - | - | - | - | - | - | - | - | - | - | - | - |
| **F.C.L.r.diag** | - | - | - | - | - | - | - | - | - | - | - | - | - | - | - |
| **S.Pe.C** | - | - | 0.49 | - | - | 0.47 | - | 0.52 | 0.52 | - | - | - | - | 0.51 | 0.50 |
| **S.F.sup** | - | - | 0.00 | - | - | 0.11 | 0.00 | - | 0.33 | 0.03 | - | 0.00 | - | - | 0.08 |
| **S.F.inter** | 0.52 | 0.43 | 0.52 | 0.30 | - | 0.43 | - | 0.41 | 0.58 | 0.49 | - | - | 0.49 | - | 0.47 |
| **S.F.inf** | 0.29 | 0.52 | 0.42 | 0.39 | - | 0.49 | 0.52 | 0.44 | 0.49 | - | 0.40 | - | 0.49 | - | 0.45 |
| **S.F.inf.ant** | - | 0.48 | - | - | - | 0.39 | 0.56 | 0.63 | 0.56 | - | - | - | 0.48 | 0.50 | 0.51 |
| **S.F.marginal** | - | 0.25 | - | - | 0.43 | 0.30 | - | - | - | - | 0.46 | - | - | 0.22 | 0.33 |
| **S.F.orbitaire** | - | 0.37 | - | - | 0.36 | 0.35 | - | 0.11 | - | - | - | - | - | 0.35 | 0.31 |
| **S.Or** | - | 0.46 | - | - | - | - | - | - | - | - | - | - | - | - | 0.46 |
| **S.T.s** | - | 0.44 | 0.44 | - | - | 0.49 | - | 0.36 | - | - | - | - | - | 0.37 | 0.42 |
| **S.T.pol** | - |  | 0.53 | - | - | 0.53 | - | 0.24 | - | - | 0.56 | - | - | - | 0.46 |
| **S.T.i.ant** | - | - | - | - | - | - | - | 0.30 | - | - | - | - | - | 0.67 | 0.49 |

Table S3. SISS index score for each sulci by each researcher in the superior view, left hemisphere.

| **Sulci** | **R1** | **R2** | **R3** | **R4** | **R5** | **R6** | **R7** | **R8** | **R9** | **R10** | **R11** | **R12** | **R13** | **R14** | **Average** |
| --- | --- | --- | --- | --- | --- | --- | --- | --- | --- | --- | --- | --- | --- | --- | --- |
| **S.C** | - | 0.36 | 0.29 | - | 0.39 | 0.39 | 0.50 | - | 0.25 | 0.37 | 0.25 | - | - | 0.42 | 0.36 |
| **F.C.L** | - | 0.37 | - | - | - | 0.41 | - | - | - | - | 0.43 | - | - | - | 0.40 |
| **F.C.L.r.ant** | - | - | - | - | - | - | - | - | - | - | - | - | - | - | - |
| **F.C.L.r.asc** | - | - | - | - | - | - | - | - | - | - | - | - | - | - | - |
| **S.Pe.C** | - | 0.36 | 0.35 | - | 0.37 | 0.34 | 0.39 | - | 0.42 | 0.58 | 0.03 | - | - | - | 0.35 |
| **S.F.sup** | - | 0.45 | 0.37 | - | - | 0.45 | - | - | - | - | - | - | - | 0.40 | 0.42 |
| **S.F.inter** | - | - | - | - | - | 0.49 | - | - | - | - | 0.20 | - | - | - | 0.34 |
| **S.F.inf** | - | - | - | - | - | 0.45 | - | - | - | - | - | - | - | - | 0.45 |
| **S.F.inf.ant** | - | 0.58 | 0.57 | - | 0.55 | - | - | - | - | - | - | - | - | - | 0.57 |
| **S.F.orbitaire** | - | - | - | - | 0.37 | - | - | - | - | - | - | - | - | - | 0.37 |
| **S.F.sup** | - | - | - | - | - | - | - | - | - | - | - | - | - | - | - |
| **S.Po.C.sup** | - | - | 0.00 | - | - | 0.13 | - | - | - | - | 0.48 | - | - | - | 0.20 |
| **F.I.P** | - | - | - | - | - | 0.46 | 0.50 | - | 0.47 | 0.51 | 0.46 | - | - | - | 0.48 |
| **F.I.P.Po.C.inf** | - | - | - | - | - | 0.45 | - | - | - | 0.50 | - | - | - | - | 0.47 |
| **F.I.P.r.int.1** | - | - | - | - | - | - | - | - | - | - | - | - | - | - | - |
| **F.I.P.r.int.2** | - | - | - | - | - | - | - | - | - | - | - | - | - | - | - |
| **S.T.s** | - | - | - | - | - | - | - | - | - | - | - | - | - | - | - |
| **S.T.s.ter.asc.ant** | - | - | - | - | - | - | - | - | - | - | - | - | - | - | - |
| **S.T.s.ter.asc.post** | - | - | - | - | - | - | - | - | - | - | - | - | - | - | - |
| **S.Pa.int** | - | - | - | - | - | - | - | - | - | - | - | - | - | - | - |
| **S.Pa.sup** | - | - | - | - | - | - | - | - | - | - | - | - | - | - | - |
| **F.P.O** | - | - | 0.39 | - | - | 0.32 | - | - | - | - | - | - | - | - | 0.35 |
| **Occipital** | - | - | - | 0.46 | - | - | - | - | - | - | - | - | - | - | 0.46 |

Table S4. SISS index score for each sulci by each researcher in the superior view, right hemisphere.

| **Sulci** | **R1** | **R2** | **R3** | **R4** | **R5** | **R6** | **R7** | **R8** | **R9** | **R10** | **R11** | **R12** | **R13** | **R14** | **Average** |
| --- | --- | --- | --- | --- | --- | --- | --- | --- | --- | --- | --- | --- | --- | --- | --- |
| **S.C** | - | 0.31 | 0.29 | - | 0.36 | 0.33 | 0.43 | - | 0.38 | 0.39 | 0.14 | - | - | 0.40 | 0.34 |
| **F.C.L** | - | 0.52 | - | - | - | 0.53 | - | - | - | - | 0.56 | - | - | - | 0.54 |
| **F.C.L.r.ant** | - | - | - | - | - | - | - | - | - | - | - | - | - | - | - |
| **F.C.L.r.asc** | - | - | - | - | - | - | - | - | - | - | - | - | - | - | - |
| **S.Pe.C** | - | 0.40 | 0.43 | - | 0.37 | 0.29 | 0.41 | 0.51 | 0.38 | 0.45 | 0.47 | - | 0.49 | - | 0.42 |
| **S.F.sup** | - | 0.47 | 0.41 | - | - | 0.42 | - | - | 0.49 | - | - | - | - | 0.46 | 0.45 |
| **S.F.inter** | - | 0.51 | - | 0.32 | 0.50 | 0.40 | - | - | 0.52 | - | 0.13 | - | - | - | 0.40 |
| **S.F.inf** | - | 0.48 | - | - | - | 0.39 | - | - | 0.56 | - | - | - | - | - | 0.48 |
| **S.F.inf.ant** | - | 0.17 | 0.20 | - | 0.48 | - | - | - | - | - | - | - | - | - | 0.28 |
| **S.F.orbitaire** | - | - | - | - | - | - | - | - | - | - | - | - | - | - | - |
| **S.F.sup** | - | - | - | - | - | - | - | - | - | - | - | - | - | - | - |
| **S.Po.C.sup** | - | - | - | - | - | 0.33 | - | 0.00 | - | 0.25 | 0.35 | - | - | - | 0.23 |
| **F.I.P** | - | - | - | - | - | 0.42 | 0.50 | 0.54 | 0.44 | 0.52 | 0.52 | - | - | - | 0.49 |
| **F.I.P.Po.C.inf** | - | - | - | - | - | 0.34 | - | - | - | - | - | - | - | - | 0.34 |
| **F.I.P.r.int.1** | - | - | - | - | - | - | - | - | - | - | - | - | - | - | - |
| **F.I.P.r.int.2** | - | - | - | - | - | - | - | - | - | - | - | - | - | - | - |
| **S.T.s** | - | - | - | - | - | - | - | - | - | - | - | - | - | - | - |
| **S.T.s.ter.asc.ant** | - | - | - | - | - | - | - | - | - | - | - | - | - | - | - |
| **S.T.s.ter.asc.post** | - | - | - | - | - | - | - | - | - | - | - | - | - | - | - |
| **S.Pa.int** | - | - | - | - | - | - | - | - | - | - | - | - | - | - | - |
| **S.Pa.sup** | - | - | - | - | - | - | - | - | - | - | - | - | - | - | - |
| **F.P.O** | - | - | 0.31 | - | - | 0.31 | - | - | - | - | - | - | - | - | 0.31 |
| **Occipital** | - | - | - | 0.41 | - | - | - | - | - | - | - | - | - | - | 0.41 |

Table S5. SISS index score for each sulci by each researcher in the posterior view, left hemisphere.

| **Sulci** | **R1** | **R2** | **R3** | **R4** | **R5** | **R6** | **R7** | **R8** | **R9** | **R10** | **R11** | **R12** | **R13** | **R14** | **Average** |
| --- | --- | --- | --- | --- | --- | --- | --- | --- | --- | --- | --- | --- | --- | --- | --- |
| **S.C** | - | - | - | - | - | - | - | - | - | - | - | - | - | - | - |
| **F.C.L** | - | - | - | - | - | - | - | 0.00 | - | - | - | - | - | - | 0.00 |
| **S.Po.C.sup** | - | - | - | - | - | - | - | - | - | - | - | - | - | - | - |
| **F.I.P** | - | 0.48 | 0.00 | - | - | 0.46 | 0.52 | 0.00 | 0.53 | 0.48 | 0.49 | - | - | - | 0.37 |
| **F.I.P.Po.C.inf** | - | - | - | - | - | - | - | - | - | - | 0.23 | - | - | - | 0.23 |
| **F.I.P.r.int.1** | - | - | - | - | - | - | - | - | - | - | - | - | - | - | 0.00 |
| **F.I.P.r.int.2** | - | - | - | - | - | - | - | - | - | - | - | - | - | - | - |
| **S.T.s** | - | 0.07 | - | - | - | - | - | - | - | - | - | - | - | - | 0.07 |
| **S.T.s.ter.asc.ant** | - | - | - | - | - | 0.34 | - | - | - | - | 0.00 | - | - | - | 0.17 |
| **S.T.s.ter.asc.post** | - | 0.45 | 0.08 | - | - | - | - | - | - | - | 0.00 | - | - | 0.41 | 0.23 |
| **S.T.i.ant** | - | 0.52 | - | - | 0.35 | - | - | - | - | - | - | - | - | - | 0.43 |
| **S.T.i.post** | - | 0.48 | - | 0.53 | 0.53 | 0.53 | - | - | - | - | 0.25 | - | - | 0.50 | 0.47 |
| **S.Pa.int** | - | - | - | - | - | - | - | - | - | - | - | - | - | - | - |
| **S.Pa.sup** | - | - | - | - | - | - | - | - | - | - | - | - | - | - | - |
| **F.P.O** | - | - | 0.00 | - | - | 0.02 | 0.52 | - | - | 0.32 | - | - | - | 0.03 | 0.18 |
| **S.O.T.lat.post** | - | - | 0.50 | - | - | 0.44 | - | - | - | - | 0.10 | - | - | 0.58 | 0.40 |
| **Occipital** | - | 0.51 | 0.50 | 0.30 | - | - | 0.51 | - | - | - | - | - | 0.54 | - | 0.47 |

Table S6. SISS index score for each sulci by each researcher in the posterior view, right hemisphere.

| **Sulci** | **R1** | **R2** | **R3** | **R4** | **R5** | **R6** | **R7** | **R8** | **R9** | **R10** | **R11** | **R12** | **R13** | **R14** | **Average** |
| --- | --- | --- | --- | --- | --- | --- | --- | --- | --- | --- | --- | --- | --- | --- | --- |
| **S.C** | - | - | - | - | - | - | - | - | - | - | - | - | - | - | - |
| **F.C.L** | - | - | - | - | - | - | - | 0.00 | - | - | - | - | - | - | 0.00 |
| **S.Po.C.sup** | - | - | - | - | - | - | - | - | - | - | - | - | - | - | - |
| **F.I.P** | - | 0.42 | 0.10 | 0.47 | - | 0.49 | 0.50 | 0.00 | 0.50 | 0.53 | 0.47 | - | - | - | 0.40 |
| **F.I.P.Po.C.inf** | - | - | - | - | - | - | - | - | - | - | - | - | - | - | - |
| **F.I.P.r.int.1** | - | - | - | - | - | - | - | 0.00 | - | - | - | - | - | - | - |
| **F.I.P.r.int.2** | - | - | - | - | - | - | - | - | - | - | - | - | - | - | - |
| **S.T.s** | - | 0.04 | - | - | - | 0.44 | - | - | - | - | - | - | - | - | 0.24 |
| **S.T.s.ter.asc.ant** | - | - | - | - | - | 0.14 | - | - | - | - | 0.00 | - | - | - | 0.07 |
| **S.T.s.ter.asc.post** | - | 0.45 | 0.41 | - | - | - | - | - | - | - | 0.08 | - | - | - | 0.31 |
| **S.T.i.ant** | - | - | - | - | 0.15 | 0.44 | - | - | - | - | - | - | - | - | 0.30 |
| **S.T.i.post** | - | 0.54 | 0.52 | 0.53 | 0.41 | 0.51 | - | - | - | - | 0.22 | - | - | 0.51 | 0.46 |
| **S.Pa.int** | - | - | - | - | - | - | - | - | - | - | - | - | - | - | - |
| **S.Pa.sup** | - | - | - | - | - | - | - | - | - | - | - | - | - | - | - |
| **F.P.O** | - | - | 0.19 | - | - | 0.01 | 0.47 | - | - | 0.29 | - | - | - | 0.00 | 0.19 |
| **S.O.T.lat.post** | - | - | - | - | - | 0.48 | - | - | - | - | 0.17 | - | - | - | 0.32 |
| **Occipital** | - | 0.51 | 0.47 | 0.45 | - | - | 0.49 | - | - | - | - | 0.45 | 0.51 | - | 0.48 |

Table S7. SISS index score for each sulci by each researcher in the lateral view, left hemisphere.

| **Sulci** | **R1** | **R2** | **R3** | **R4** | **R5** | **R6** | **R7** | **R8** | **R9** | **R10** | **R11** | **R12** | **R13** | **R14** | **Average** |
| --- | --- | --- | --- | --- | --- | --- | --- | --- | --- | --- | --- | --- | --- | --- | --- |
| **S.C** | - | 0.27 | 0.27 | - | - | 0.24 | 0.48 | - | 0.24 | 0.25 | 0.34 | - | - | 0.44 | 0.32 |
| **F.C.L** | - | 0.42 | 0.50 | 0.43 | 0.50 | 0.51 | 0.53 | 0.34 | 0.50 | 0.31 | 0.40 | 0.36 | 0.53 | 0.53 | 0.45 |
| **F.C.L.r.ant.** | - | 0.43 | 0.51 | - | - | 0.49 | 0.41 | - | - | - | - | - | - | - | 0.46 |
| **F.C.L.r.asc** | 0.52 | - | 0.43 | 0.49 | - | - | 0.54 | - | 0.60 | - | - | 0.63 | - | 0.62 | 0.55 |
| **F.C.L.r.diag** | - | - | - | - | - | - | - | - | - | - | - | - | - | - | - |
| **S.Pe.C** | - | 0.23 | 0.27 | 0.35 | 0.46 | 0.26 | 0.44 | 0.08 | 0.37 | 0.34 | - | - | - | - | 0.31 |
| **S.F.sup** | - | - | - | - | - | 0.28 | - | - | - | - | - | - | - | 0.36 | 0.32 |
| **S.F.inter** | - | - | 0.48 | - | - | 0.43 | 0.50 | 0.48 | - | - | - | - | - | 0.47 | 0.47 |
| **S.F.inf** | - | - | 0.37 | 0.30 | - | 0.43 | 0.56 | - | - | - | 0.33 | - | - | - | 0.40 |
| **S.F.inf.ant** | 0.59 | 0.58 | - | - | 0.56 | 0.51 | 0.64 | 0.46 | - | - | 0.39 | - | 0.56 | 0.58 | 0.54 |
| **S.F.marginal** | - | - | - | - | - | 0.21 | - | - | - | - | - | - | - | 0.52 | 0.36 |
| **S.F.orbitaire** | - | 0.52 | - | - | 0.47 | 0.27 | - | 0.21 | - | - | - | - | 0.54 | 0.53 | 0.43 |
| **S.Or** | - | - | - | - | - | - | - | - | - | - | - | - | - | 0.46 | 0.46 |
| **S.Po.C.sup** | - | 0.00 | - | - | - | 0.19 | - | 0.00 | - | - | - | - | - | - | 0.07 |
| **F.I.P** | - | 0.43 | - | - | - | 0.36 | 0.48 | - | 0.47 | 0.44 | - | - | - | - | 0.44 |
| **F.I.P.Po.C.inf** | - | - | 0.08 | - | - | 0.07 | - | - | - | - | 0.47 | - | - | 0.48 | 0.21 |
| **F.I.P.r.int.1** | - | - | - | - | - | - | - | - | - | - | - | - | - | - | - |
| **F.I.P.r.int.2** | - | 0.23 | - | - | - | - | - | - | - | - | - | - | - | - | 0.23 |
| **S.T.s** | 0.24 | 0.41 | 0.49 | 0.24 | 0.51 | 0.41 | 0.45 | - | 0.34 | 0.28 | 0.31 | - | 0.56 | 0.51 | 0.40 |
| **S.T.s.ter.asc.ant** | - | 0.31 | - | - | - | 0.40 | - | - | 0.19 | - | - | - | - | - | 0.30 |
| **S.T.s.ter.asc.post** | - | 0.00 | - | - | - | 0.52 | - | - | - | - | - | - | - | - | 0.26 |
| **S.T.pol** | - | 0.47 | - | 0.58 | - | 0.52 | - | 0.41 | - | - | 0.56 | - | - | - | 0.51 |
| **S.T.i.ant** | - | 0.44 | 0.26 | 0.21 | 0.47 | 0.42 | 0.40 | 0.31 | 0.39 | 0.47 | 0.41 | - | 0.44 | 0.27 | 0.37 |
| **S.T.i.post** | - | 0.54 | 0.51 | 0.53 | - | 0.49 | - | 0.00 | 0.52 | - | 0.52 | - | - | 0.42 | 0.44 |
| **S.Pa.int** | - | - | - | - | - | - | - | - | - | - | - | - | - | - | - |
| **S.Pa.sup** | - | - | - | - | - | - | - | - | - | - | - | - | - | - | - |
| **F.P.O** | - | - | - | - | - | - | - | - | 0.34 | - | - | - | - | - | - |
| **S.O.T.lat.post** | - | - | - | - | - | 0.46 | - | - | - | - | 0.19 | - | - | - | 0.32 |
| **Occipital** | - | - | - | 0.42 | - | - | - | - | - | - | - | - | - | - | 0.42 |

Table S8. SISS index score for each sulci by each researcher in the lateral view, right hemisphere.

| **Sulci** | **R1** | **R2** | **R3** | **R4** | **R5** | **R6** | **R7** | **R8** | **R9** | **R10** | **R11** | **R12** | **R13** | **R14** | **Average** |
| --- | --- | --- | --- | --- | --- | --- | --- | --- | --- | --- | --- | --- | --- | --- | --- |
| **S.C** | 0.46 | 0.52 | 0.49 | - | - | 0.43 | 0.49 | - | 0.34 | 0.52 | - | - | - | 0.57 | 0.48 |
| **F.C.L** | 0.46 | 0.36 | - | 0.32 | 0.44 | 0.37 | 0.45 | 0.42 | 0.36 | 0.45 | 0.32 | - | - | 0.51 | 0.41 |
| **F.C.L.r.ant.** | - | - | - | 0.56 | 0.22 | 0.19 | 0.60 | - | 0.58 | - | 0.47 | - | - | 0.42 | 0.43 |
| **F.C.L.r.asc** | - | - | - | 0.47 | - | - | 0.61 | - | 0.70 | - | 0.69 | - | - | 0.39 | 0.57 |
| **F.C.L.r.diag** | - | 0.41 | 0.35 | - | - | - | - | - | - | - | - | - | - | - | 0.38 |
| **S.Pe.C** | - | 0.45 | 0.47 | 0.45 | 0.28 | 0.42 | 0.53 | 0.35 | 0.35 | 0.52 | - | - | - | 0.48 | 0.43 |
| **S.F.sup** | - | 0.37 | - | - | - | 0.26 | - | 0.40 | 0.39 | 0.43 | - | - | - | 0.40 | 0.38 |
| **S.F.inter** | - | 0.50 | 0.49 | 0.18 | - | 0.32 | 0.54 | 0.55 | 0.63 | - | - | - | 0.40 | 0.34 | 0.44 |
| **S.F.inf** | 0.20 | 0.34 | 0.30 | 0.15 | - | 0.35 | 0.44 | - | 0.38 | 0.49 | - | - | - | 0.48 | 0.35 |
| **S.F.inf.ant** | - | 0.51 | - | - | 0.18 | 0.54 | 0.69 | 0.30 | 0.64 | - | - | - | - | 0.34 | 0.46 |
| **S.F.marginal** | - | - | - | - | - | - | - | - | - | - | - | - | 0.00 | - | 0.00 |
| **S.F.orbitaire** | - | - | - | - | 0.23 | 0.46 | - | 0.21 | - | - | - | - | - | 0.14 | 0.26 |
| **S.Or** | - | - | - | - | - | - | - | - | - | - | - | - | - | 0.48 | 0.48 |
| **S.Po.C.sup** | - | - | - | - | - | 0.10 | 0.63 | - | - | - | - | - | - | - | 0.36 |
| **F.I.P** | - | 0.51 | 0.32 | - | - | 0.46 | 0.49 | 0.49 | 0.56 | 0.46 | - | - | - | 0.52 | 0.48 |
| **F.I.P.Po.C.inf** | - | - | - | - | - | - | - | - | - | - | - | - | - | - | - |
| **F.I.P.r.int.1** | - | - | - | - | - | - | - | - | - | - | - | - | - | - | - |
| **F.I.P.r.int.2** | 0.48 | - | - | - | - | - | - | - | - | - | - | - | - | - | 0.48 |
| **S.T.s** | - | 0.43 | 0.45 | 0.47 | - | 0.34 | 0.47 | - | 0.53 | 0.44 | - | - | 0.45 | 0.52 | 0.46 |
| **S.T.s.ter.asc.ant** | - | 0.40 | - | - | - | 0.16 | - | - | 0.54 | - | - | - | - | - | 0.37 |
| **S.T.s.ter.asc.post** | - | 0.33 | 0.25 | - | - | 0.31 | - | - | - | - | - | - | 0.00 | - | 0.22 |
| **S.T.pol** | - | 0.52 | - | - | - | 0.27 | - | - | 0.51 | - | - | - | - | - | 0.43 |
| **S.T.i.ant** | 0.24 | 0.52 | 0.38 | 0.26 | 0.47 | 0.33 | 0.45 | 0.34 | 0.42 | 0.37 | - | - | - | 0.46 | 0.39 |
| **S.T.i.post** | - | 0.51 | 0.41 | 0.51 | - | 0.52 | - | - | 0.47 | - | - | - | 0.48 | 0.46 | 0.48 |
| **S.Pa.int** | - | - | - | - | - | - | - | - | - | - | - | - | - | - | - |
| **S.Pa.sup** | - | - | - | - | - | - | - | - | - | - | - | - | - | - | - |
| **F.P.O** | - | - | 0.00 | - | - | 0.00 | 0.19 | - | - | 0.00 | - | - | - | 0.00 | 0.04 |
| **S.O.T.lat.post** | - | - | - | - | - | - | - | - | - | - | - | - | - | - | - |
| **Occipital** | - | - | - | 0.46 | - | - | - | - | - | - | - | - | 0.52 | - | 0.49 |

Table S9. SISS index score for each sulci by each researcher in the anterior view, left hemisphere.

| **Sulci** | **R1** | **R2** | **R3** | **R4** | **R5** | **R6** | **R7** | **R8** | **R9** | **R10** | **R11** | **R12** | **R13** | **R14** | **Average** |
| --- | --- | --- | --- | --- | --- | --- | --- | --- | --- | --- | --- | --- | --- | --- | --- |
| **S.C** | - | - | - | - | - | 0.51 | 0.52 | - | 0.46 | - | 0.65 | - | - | - | 0.54 |
| **F.C.L** | - | - | - | - | - | 0.51 | - | - | - | - | - | - | - | - | 0.51 |
| **F.C.L.r.ant.** | - | - | 0.49 | - | - | - | - | - | - | - | - | - | - | - | 0.49 |
| **F.C.L.r.asc** | - | - | 0.54 | 0.60 | - | 0.51 | 0.64 | - | - | - | 0.53 | - | - | 0.55 | 0.56 |
| **F.C.L.r.diag** | - | - | 0.51 | - | - | - | - | - | - | - | - | - | - | - | 0.51 |
| **S.Pe.C** | - | - | - | - | - | - | - | - | 0.46 | - | - | - | - | - | 0.46 |
| **S.F.sup** | - | - | - | - | - | - | - | - | - | - | - | - | - | - | - |
| **S.F.inter** | 0.49 | 0.54 | 0.49 | - | - | 0.51 | - | - | - | - | 0.54 | 0.50 | - | 0.48 | 0.51 |
| **S.F.inf** | - | 0.52 | 0.53 | - | - | 0.46 | 0.51 | - | - | 0.44 | - | - | - | - | 0.49 |
| **S.F.inf.ant** | 0.64 | 0.56 | 0.59 | - | 0.48 | 0.52 | 0.63 | 0.48 | - | - | 0.53 | - | - | 0.66 | 0.57 |
| **S.F.marginal** | - | - | 0.53 | - | - | - | 0.57 | - | - | - | 0.62 | - | 0.58 | 0.59 | 0.58 |
| **S.F.orbitaire** | - | - | - | - | - | - | - | - | - | - | - | - | - | - | - |
| **S.Or** | - | 0.50 | - | - | - | - | - | - | - | - | - | - | - |  | 0.50 |
| **S.T.s** | - | 0.52 | - | - | - | 0.50 | - | - | - | - | - | - | - | 0.54 | 0.52 |
| **S.T.pol** | - | 0.51 | 0.53 | - | - | 0.55 | - | - | - | - | 0.53 | - | - | 0.58 | 0.54 |
| **S.T.i.ant** | - | - | - | - | - | - | - | - | - | - | - | - | - | 0.67 | 0.67 |

Table S10. SISS index score for each sulci by each researcher in the anterior view, right hemisphere.

| **Sulci** | **R1** | **R2** | **R3** | **R4** | **R5** | **R6** | **R7** | **R8** | **R9** | **R10** | **R11** | **R12** | **R13** | **R14** | **Average** |
| --- | --- | --- | --- | --- | --- | --- | --- | --- | --- | --- | --- | --- | --- | --- | --- |
| **S.C** | - | - | - | - | - | 0.54 | - | - | 0.55 | - | 0.62 | - | - | - | 0.57 |
| **F.C.L** | - | 0.45 | 0.51 | - | - | 0.51 | 0.44 | - | - | - | - | - | - | - | 0.49 |
| **F.C.L.r.ant.** | - | - | - | - | - | - | - | - | - | - | - | - | - | - | - |
| **F.C.L.r.asc** | - | - | 0.53 | - | - | - | - | - | - | - | 0.53 | - | - | - | 0.53 |
| **F.C.L.r.diag** | - | - | - | - | - | - | - | - | - | - | - | - | - | - | - |
| **S.Pe.C** | - | 0.48 | 0.49 | 0.50 | - | 0.47 | - | 0.52 | 0.52 | - | - | - | - | 0.51 | 0.50 |
| **S.F.sup** | - | - | - | - | - | - | - | - | 0.49 | - | - | - | - | - | 0.49 |
| **S.F.inter** | 0.52 | 0.43 | 0.52 | - | 0.54 | 0.51 | 0.54 | 0.45 | 0.58 | - | - | - | 0.56 | 0.56 | 0.52 |
| **S.F.inf** | - | 0.52 | - | - | - | 0.49 | 0.52 | - | 0.50 | 0.49 | 0.55 | - | 0.58 | 0.56 | 0.53 |
| **S.F.inf.ant** | - | 0.55 | 0.48 | 0.44 | 0.52 | - | 0.56 | 0.63 | 0.56 | - | - | - | 0.48 | 0.50 | 0.53 |
| **S.F.marginal** | 0.71 | - | 0.51 | 0.58 | - | - | - | - | - | - | 0.75 | - | - | - | 0.64 |
| **S.F.orbitaire** | - | - | - | - | - | - | - | - | - | - | - | - | - | - | - |
| **S.Or** | - | - | 0.51 | - | - | - | - | - | - | - | - | - | - | - | 0.51 |
| **S.T.s** | - | - | 0.52 | - | - | 0.49 | - | - | - | - | - | - | - | - | 0.51 |
| **S.T.pol** | - | 0.53 | 0.53 | - | - | 0.53 | - | - | - | - | 0.56 | - | - | 0.60 | 0.55 |
| **S.T.i.ant** | - | - | - | - | - | - | - | - | - | - | - | - | - | - | - |

Table S11. SISS index score for each sulci by each researcher in the superior view, left hemisphere.

| **Sulci** | **R1** | **R2** | **R3** | **R4** | **R5** | **R6** | **R7** | **R8** | **R9** | **R10** | **R11** | **R12** | **R13** | **R14** | **Average** |
| --- | --- | --- | --- | --- | --- | --- | --- | --- | --- | --- | --- | --- | --- | --- | --- |
| **S.C** | - | - | 0.49 | - | - | 0.45 | 0.50 | - | - | - | - | - | - | - | 0.48 |
| **F.C.L** | - | - | - | - | - | 0.41 | - | - | - | - | 0.43 | - | - | - | 0.42 |
| **F.C.L.r.ant.** | - | - | - | - | - | - | - | - | - | - | - | - | - | - | - |
| **F.C.L.r.asc** | - | - | - | - | - | - | 0.64 | - | - | - | - | - | - | - | 0.64 |
| **S.Pe.C** | - | - | - | - | - | - | 0.50 | - | 0.51 | 0.58 | - | - | - | - | 0.53 |
| **S.F.sup** | - | 0.45 | - | - | - | 0.45 | - | - | - | - | - | - | - | - | 0.45 |
| **S.F.inter** | - | - | - | - | 0.50 | 0.49 | - | - | - | - | 0.48 | - | - | - | 0.49 |
| **S.F.inf** | - | - | - | - | - | 0.45 | - | - | - | - | - | - | - | - | 0.45 |
| **S.F.inf.ant** | - | 0.58 | 0.57 | - | 0.57 | - | - | - | - | - | - | - | - | - | 0.58 |
| **S.F.orbitaire** | - | - | - | - | - | - | - | - | - | - | - | - | - | - | - |
| **S.Po.C.sup** | - | - | - | - | - | - | - | - |  |  | 0.48 | - | - | - | 0.48 |
| **F.I.P** | - | - | 0.51 | - | - | 0.48 | 0.50 | - | 0.47 | 0.51 | 0.48 | - | - | - | 0.49 |
| **F.I.P.Po.C.inf** | - | - | - | - | - | 0.45 | - | - | - | 0.50 | - | - | - | - | 0.47 |
| **F.I.P.r.int.1** | - | - | - | - | - | - | - | - | - | - | - | - | - | - | - |
| **F.I.P.r.int.2** | - | - | - | - | - | - | - | - | - | - | - | - | - | - | - |
| **S.T.s** | - | - | - | - | - | - | - | - | - | - | - | - | - | - | - |
| **S.T.s.ter.asc.ant** | - | - | - | - | - | - | - | - | - | - | - | - | - | - | - |
| **S.T.s.ter.asc.post** | - | - | - | - | - | - | - | - | - | - | - | - | - | - | - |
| **S.Pa.int** | - | - | - | - | - | - | - | - | - | - | - | - | - | - | - |
| **S.Pa.sup** | - | - | - | - | - | - | - | - | - | - | - | - | - | - | - |
| **F.P.O** | - | - | - | - | - | - | - | - | - | - | - | - | - | - | - |
| **Occipital** | - | - | - | 0.53 | - | - | - | - | - | - | - | - | - | - | 0.53 |

Table S12. SISS index score for each sulci by each researcher in the superior view, right hemisphere.

| **Sulci** | **R1** | **R2** | **R3** | **R4** | **R5** | **R6** | **R7** | **R8** | **R9** | **R10** | **R11** | **R12** | **R13** | **R14** | **Average** |
| --- | --- | --- | --- | --- | --- | --- | --- | --- | --- | --- | --- | --- | --- | --- | --- |
| **S.C** | - | - | - | - | - | 0.44 | 0.43 | - | - | - | - | - | - | 0.40 | 0.42 |
| **F.C.L** | - | 0.52 | 0.57 | - | - | 0.53 | - | - | - | - | 0.56 | - | - | - | 0.55 |
| **F.C.L.r.ant.** | - | - | - | - | - | - | - | - | - | - | - | - | - | - | - |
| **F.C.L.r.asc** | - | - | - | - | - | - | 0.46 | - | - | - | - | - | - | - | 0.46 |
| **S.Pe.C** | - | 0.48 | 0.46 | 0.51 | - | - | - | 0.51 | 0.47 | 0.45 | 0.47 | - | 0.49 | - | 0.48 |
| **S.F.sup** | - | 0.47 | - | - | - | 0.42 | - | - | 0.48 | - | 0.49 | - | - | 0.46 | 0.46 |
| **S.F.inter** | - | 0.51 | - | - | 0.50 | 0.40 | - | - | 0.52 | - | 0.49 | - | - | - | 0.48 |
| **S.F.inf** | - | 0.51 | 0.49 | - | - | - | - | - | 0.56 | - | - | - | - | - | 0.52 |
| **S.F.inf.ant** | - | - | - | - | 0.46 | - | - | - | - | - | - | - | - | - | 0.46 |
| **S.F.orbitaire** | - | - | - | - | - | - | - | - | - | - | - | - | - | - | - |
| **S.Po.C.sup** | - | - | - | - | - | - | - | - | - | - | - | - | - | - | - |
| **F.I.P** | - | - | 0.47 | - | - | 0.43 | 0.50 | 0.54 | 0.44 | 0.52 | 0.52 | - | - | - | 0.49 |
| **F.I.P.Po.C.inf** | - | - | - | - | - | - | - | - | - | - | - | - | - | - | - |
| **F.I.P.r.int.1** | - | - | - | - | - | - | - | - | - | - | - | - | - | - | - |
| **F.I.P.r.int.2** | - | - | - | - | - | - | - | - | - | - | - | - | - | - | - |
| **S.T.s** | - | - | - | - | - | - | - | - | - | - | - | - | - | - | - |
| **S.T.s.ter.asc.ant** | - | - | - | - | - | - | - | - | - | - | - | - | - | - | - |
| **S.T.s.ter.asc.post** | - | - | - | - | - | - | - | - | - | - | - | - | - | - | - |
| **S.Pa.int** | - | - | - | - | - | - | - | - | - | - | - | - | - | - | - |
| **S.Pa.sup** | - | - | - | - | - | - | - | - | - | - | - | - | - | - | - |
| **F.P.O** | - | - | - | - | - | - | - | - | - | - | - | - | - | - | - |
| **Occipital** | - | - | - | 0.51 | - | - | - | - | - | - | - | - | - | - | 0.51 |

Table S13. SISS index score for each sulci by each researcher in the posterior view, left hemisphere.

| **Sulci** | **R1** | **R2** | **R3** | **R4** | **R5** | **R6** | **R7** | **R8** | **R9** | **R10** | **R11** | **R12** | **R13** | **R14** | **Average** |
| --- | --- | --- | --- | --- | --- | --- | --- | --- | --- | --- | --- | --- | --- | --- | --- |
| **S.C** | - | - | - | - | - | - | - | - | - | - | - | - | - | - | - |
| **F.C.L** | - | - | - | - | - | - | - | - | - | - | - | - | - | - | - |
| **S.Po.C.sup** | - | - | - | - | - | - | - | - | - | - | - | - | - | - | - |
| **F.I.P** | - | 0.48 | - | 0.49 | 0.5 | 0.46 | 0.52 | - | 0.53 | 0.48 | 0.49 | - | 0.47 | 0.47 | 0.49 |
| **F.I.P.Po.C.inf** | - | - | - | - | - | - | - | - | - | - | - | - | - | - | - |
| **F.I.P.r.int.1** | - | - | - | - | - | - | - | - | - | - | - | - | - | - | - |
| **F.I.P.r.int.2** | - | - | - | - | - | - | - | - | - | - | - | - | - | - | - |
| **S.T.s** | - | - | - | - | - | - | - | - | - | - | - | - | - | - | - |
| **S.T.s.ter.asc.ant** | - | - | - | - | - | - | - | - | - | - | - | - | - | - | - |
| **S.T.s.ter.asc.post** | - | 0.45 | 0.49 | - | 0.57 | 0.51 | - | 0.49 | - | - | - | - | - | 0.41 | 0.49 |
| **S.T.i.ant** | - | 0.52 | - | - | - | - | - | - | - | - | - | - | - | - | 0.52 |
| **S.T.i.post** | - | 0.51 | 0.52 | 0.53 | 0.49 | 0.53 | 0.48 | - | - | - | 0.53 | - | - | 0.51 | 0.51 |
| **S.Pa.int** | - | - | - | - | - | - | - | - | - | - | - | - | - | - | - |
| **S.Pa.sup** | - | - | - | - | - | - | - | - | - | - | - | - | - | - | - |
| **F.P.O** | - | - | - | - | - | - | 0.52 | - | - | - | - | - | - | - | 0.52 |
| **S.O.T.lat.post** | - | - | 0.50 | 0.49 | - | 0.44 | - | - | - | - | 0.51 | - | - | 0.58 | 0.50 |
| **Occipital** | - | 0.51 | 0.50 | 0.49 | - | - | 0.51 | - | - | - | - | 0.48 | 0.54 | 0.51 | 0.50 |

Table S14. SISS index score for each sulci by each researcher in the posterior view, right hemisphere.

| **Sulci** | **R1** | **R2** | **R3** | **R4** | **R5** | **R6** | **R7** | **R8** | **R9** | **R10** | **R11** | **R12** | **R13** | **R14** | **Average** |
| --- | --- | --- | --- | --- | --- | --- | --- | --- | --- | --- | --- | --- | --- | --- | --- |
| **S.C** | - | - | - | - | - | - | - | - | - | - | - | - | - | - | - |
| **F.C.L** | - | - | - | - | - | - | - | - | - | - | - | - | - | - | - |
| **S.Po.C.sup** | - | - | - | - | - | - | - | - | - | - | - | - | - | - | - |
| **F.I.P** | - | 0.42 | 0.47 | 0.47 | 0.46 | 0.49 | 0.50 | - | 0.50 | 0.53 | 0.47 | - | - | - | 0.48 |
| **F.I.P.Po.C.inf** | - | - | - | - | - | - | - | - | - | - | - | - | - | - | - |
| **F.I.P.r.int.1** | - | - | - | - | - | - | - | - | - | - | - | - | - | - | - |
| **F.I.P.r.int.2** | - | - | - | - | - | - | - | - | - | - | - | - | - | - | - |
| **S.T.s** | - | - | - | - | - | 0.44 | - | - | - | - | - | - | - | - | 0.44 |
| **S.T.s.ter.asc.ant** | - | - | - | - | - | - | - | - | - | - | - | - | - | - | - |
| **S.T.s.ter.asc.post** | - | 0.45 | - | - | 0.45 | - | - | - | - | - | 0.42 | - | - | - | 0.44 |
| **S.T.i.ant** | - | - | - | - | - | 0.44 | - | - | - | - | - | - | - | - | 0.44 |
| **S.T.i.post** | - | 0.54 | 0.52 | 0.53 | 0.53 | 0.51 | - | 0.47 | - | - | - | - | - | 0.51 | 0.50 |
| **S.Pa.int** |  | - | - | - | - | - | - | - | - | - | - | - | - | - | - |
| **S.Pa.sup** | - | - | - | - | - | - | - | - | - | - | - | - | - | - | - |
| **F.P.O** | - | - | - | - | - | - | 0.47 | - | - | - | - | - | - | - | 0.47 |
| **S.O.T.lat.post** | - | - | - | - | - | 0.48 | - | 0.48 | - | - | - | - | - | - | 0.48 |
| **Occipital** | - | 0.51 | 0.47 | 0.48 | - | - | 0.49 | - | - | - | - | 0.48 | 0.51 | 0.45 | 0.49 |

Table S15. SISS index score for each sulci by each researcher in the lateral view, left hemisphere.

| **Sulci** | **R1** | **R2** | **R3** | **R4** | **R5** | **R6** | **R7** | **R8** | **R9** | **R10** | **R11** | **R12** | **R13** | **R14** | **Average** |
| --- | --- | --- | --- | --- | --- | --- | --- | --- | --- | --- | --- | --- | --- | --- | --- |
| **S.C** | - | - | - | - | - | - | 0.48 | - | - | - | - | - | - | 0.44 | 0.46 |
| **F.C.L** | 0.52 | 0.47 | 0.51 | 0.48 | 0.50 | 0.50 | 0.53 | - | 0.50 | 0.48 | 0.50 | - | 0.53 | 0.53 | 0.50 |
| **F.C.L.r.ant.** | - | 0.43 | 0.52 | - | - | 0.49 | - | - | - | - | - | - | - | - | 0.48 |
| **F.C.L.r.asc** | 0.52 | 0.51 | 0.43 | 0.49 | - | 0.51 | 0.54 | 0.56 | 0.60 | - | - | 0.63 | - | 0.62 | 0.54 |
| **F.C.L.r.diag** | - | - | - | - | - | - | - | - | - | - | - | - | - | - | - |
| **S.Pe.C** | - | 0.52 | 0.48 | - | 0.46 | 0.47 | 0.52 | 0.50 | 0.49 | - | - | - | - | - | 0.49 |
| **S.F.sup** | - | - | - | - | - | - | - | - | - | - | - | - | - | 0.52 | 0.52 |
| **S.F.inter** | - | - | 0.48 | - | - | 0.43 | 0.50 | 0.48 | - | - | - | - | - | 0.47 | 0.47 |
| **S.F.inf** | - | - | - | - | - | 0.43 | 0.56 | - | - | - | - | - | - | - | 0.50 |
| **S.F.inf.ant** | 0.59 | 0.58 | 0.54 | 0.54 | 0.56 | 0.51 | 0.64 | 0.46 | - | - | 0.57 | - | 0.56 | 0.64 | 0.56 |
| **S.F.marginal** | - | - | - | - | - | - | - | - | - | - | - | - | - | 0.522 | 0.52 |
| **S.F.orbitaire** | - | 0.52 | - | - | 0.47 | 0.53 | - | - | - | - | - | - | 0.54 | 0.53 | 0.52 |
| **S.Or** | - | - | - | - | - | - | - | - | - | - | - | - | - | 0.46 | 0.46 |
| **S.Po.C.sup** | - | - | - | - | - | - | - | - | - | - | - | - | - | - | - |
| **F.I.P** | - | 0.43 | - | - | - | - | 0.48 | - | 0.47 | 0.44 | - | - | - | 0.48 | 0.46 |
| **F.I.P.Po.C.inf** | - | 0.43 | - | - | - | - | - | - | - | - | 0.47 | - | - | - | 0.45 |
| **F.I.P.r.int.1** | - | - | - | - | - | - | - | - | - | - | - | - | - | - | - |
| **F.I.P.r.int.2** | - | - | - | - | - | - | - | - | - | - | - | - | - | - | - |
| **S.T.s** | - | 0.45 | 0.49 | - | 0.51 | 0.47 | 0.53 | - | 0.46 | - | - | - | 0.56 | 0.51 | 0.50 |
| **S.T.s.ter.asc.ant** | - | - | - | - | - | 0.40 | - | - | - | - | - | - | - | - | 0.40 |
| **S.T.s.ter.asc.post** | - | 0.51 | - | - | - | 0.52 | - | - | - | - | - | - | - | 0.49 | 0.51 |
| **S.T.pol** | - | 0.47 | 0.52 | 0.58 | 0.49 | 0.52 | 0.53 | 0.41 | 0.55 | - | 0.56 | - | - | 0.54 | 0.52 |
| **S.T.i.ant** | - | 0.44 | - | - | 0.49 | 0.48 | 0.40 | - | 0.44 | 0.47 | 0.41 | - | 0.44 | - | 0.45 |
| **S.T.i.post** | - | 0.54 | 0.51 | 0.53 | - | 0.49 | - | - | 0.52 | - | 0.52 | - | - | 0.42 | 0.50 |
| **S.Pa.int** | - | - | - | - | - | - | - | - | - | - | - | - | - | - | - |
| **S.Pa.sup** | - | - | - | - | - | - | - | - | - | - | - | - | - | - | - |
| **F.P.O** | - | - | - | - | - | - | - | - | - | - | - | - | - | - | - |
| **S.O.T.lat.post** | - | - | - | - | - | 0.46 | - | - | - | - | - | - | - | - | 0.46 |
| **Occipital** | - | - | - | 0.51 | - | - | - | - | - | - | 0.56 | - | - | 0.49 | 0.52 |

Table S16. SISS index score for each sulci by each researcher in the lateral view, right hemisphere.

| **Sulci** | **R1** | **R2** | **R3** | **R4** | **R5** | **R6** | **R7** | **R8** | **R9** | **R10** | **R11** | **R12** | **R13** | **R14** | **Average** |
| --- | --- | --- | --- | --- | --- | --- | --- | --- | --- | --- | --- | --- | --- | --- | --- |
| **S.C** | 0.46 | 0.52 | 0.49 | 0.49 | 0.48 | 0.43 | 0.49 | - | - | 0.54 | - | - | - | 0.57 | 0.50 |
| **F.C.L** | 0.46 | - | 0.51 | - | 0.44 | - | 0.45 | 0.51 | 0.51 | 0.45 | - | - | - | 0.51 | 0.48 |
| **F.C.L.r.ant.** | - | - | - | 0.56 | - | - | 0.60 | - | 0.58 | - | - | 0.47 | - | 0.42 | 0.53 |
| **F.C.L.r.asc** | - | - | 0.61 | 0.47 | 0.66 | - | 0.61 | 0.70 | 0.70 | - | - | 0.69 | - | - | 0.63 |
| **F.C.L.r.diag** | - | 0.41 | - | - | - | - | - | - | - | - | - | - | - | - | 0.41 |
| **S.Pe.C** | - | 0.48 | 0.48 | 0.51 | - | 0.42 | 0.53 | 0.50 | - | 0.52 | - | - | - | 0.48 | 0.49 |
| **S.F.sup** | - | - | - | - | - | - | - | 0.40 | - | 0.43 | - | - | - | 0.40 | 0.41 |
| **S.F.inter** | - | 0.50 | 0.49 | - | - | - | 0.54 | 0.55 | 0.63 | 0.44 | - | - | 0.46 | 0.41 | 0.50 |
| **S.F.inf** | - | - | - | - | 0.46 | - | 0.44 | - | 0.40 | 0.49 | - | - | - | 0.48 | 0.45 |
| **S.F.inf.ant** | 0.63 | 0.51 | 0.57 | 0.72 | 0.47 | 0.54 | 0.69 | - | 0.64 | - | - | - | 0.41 | 0.45 | 0.56 |
| **S.F.marginal** | - | - | - | - | - | - | - | - | - | - | - | - | - | - | - |
| **S.F.orbitaire** | - | - | - | 0.66 | - | 0.46 | - | - | - | - | - | - | - | - | 0.56 |
| **S.Or** | - | - | - | - | - | - | - | - | - | - | - | - | - | 0.48 | 0.48 |
| **S.Po.C.sup** | - | - | - | - | - | - | 0.63 | - | - | - | - | - | - | - | 0.63 |
| **F.I.P** | - | 0.51 | 0.46 | 0.46 | - | 0.46 | 0.49 | 0.49 | 0.56 | 0.47 | - | - | - | 0.52 | 0.49 |
| **F.I.P.Po.C.inf** | - | - | - | - | - | - | - | - | - | - | - | - | - | - | - |
| **F.I.P.r.int.1** | - | - | - | - | - | - | - | - | - | - | - | - | - | - | - |
| **F.I.P.r.int.2** | - | - | - | - | - | - | - | - | - | - | - | - | - | - | - |
| **S.T.s** | 0.48 | 0.43 | 0.45 | 0.50 |  | 0.48 | 0.47 | - | 0.49 | 0.44 | - | 0.47 | 0.45 | 0.52 | 0.47 |
| **S.T.s.ter.asc.ant** | - | 0.40 | - | - | - | - | - | - | 0.54 | - | - | - | - | - | 0.47 |
| **S.T.s.ter.asc.post** | - | - | 0.52 | - | - | - | - | - | - | - | - | - | - | 0.40 | 0.46 |
| **S.T.pol** | - | 0.52 | - | 0.60 | - | - | - | - | 0.51 | - | - | - | - | - | 0.54 |
| **S.T.i.ant** | - | 0.52 | - | - | 0.47 | 0.47 | 0.45 | - | 0.42 | 0.46 | - | - | - | 0.46 | 0.46 |
| **S.T.i.post** | - | 0.51 | 0.49 | 0.51 | - | 0.50 | - | - | 0.49 | - | - | - | 0.48 | 0.46 | 0.49 |
| **S.Pa.int** | - | - | - | - | - | - | - | - | - | - | - | - | - | - | - |
| **S.Pa.sup** | - | - | - | - | - | - | - | - | - | - | - | - | - | - | - |
| **F.P.O** | - | - | - | - | - | - | - | - | - | - | - | - | - | - | - |
| **S.O.T.lat.post** | - | - | - | - | - | - | - | - | 0.54 | - | - | - | - | - | 0.54 |
| **Occipital** | - | - | 0.49 | 0.49 | - | - | - | - | - | - | - | - | 0.52 | 0.47 | 0.49 |


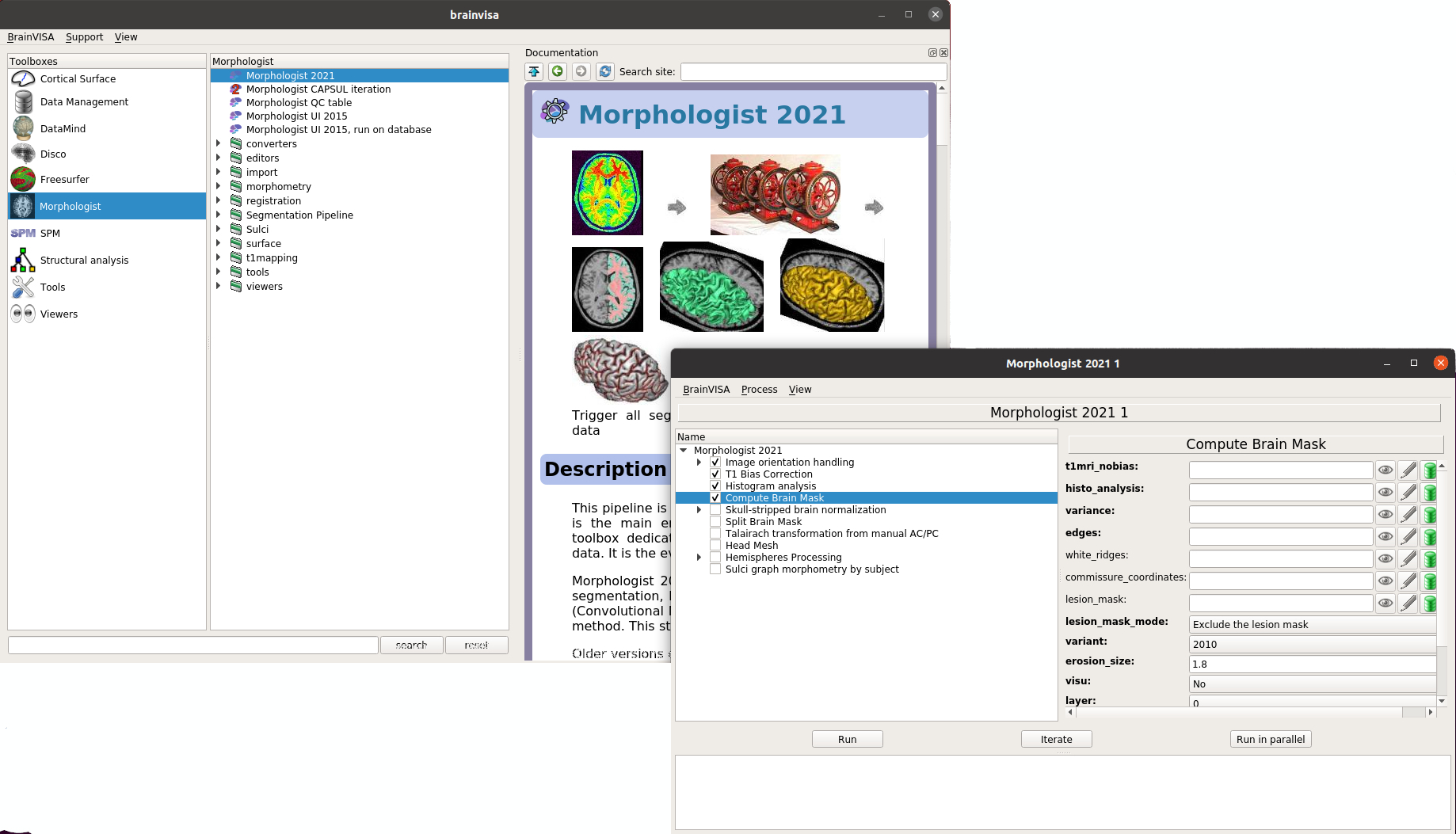


Figure S1. Morphologist toolbox of Brainvisa. The pipeline was used to segment the brain into different structures (pial surface mesh, white matter mesh and sulci) from a T1-MPRAGE image. The first 4 steps of the same pipeline with slightly adjusted parameters were used to obtain a brain mask fully covering all the structures under the dura mater as base for the creation of the proxy-endocast.


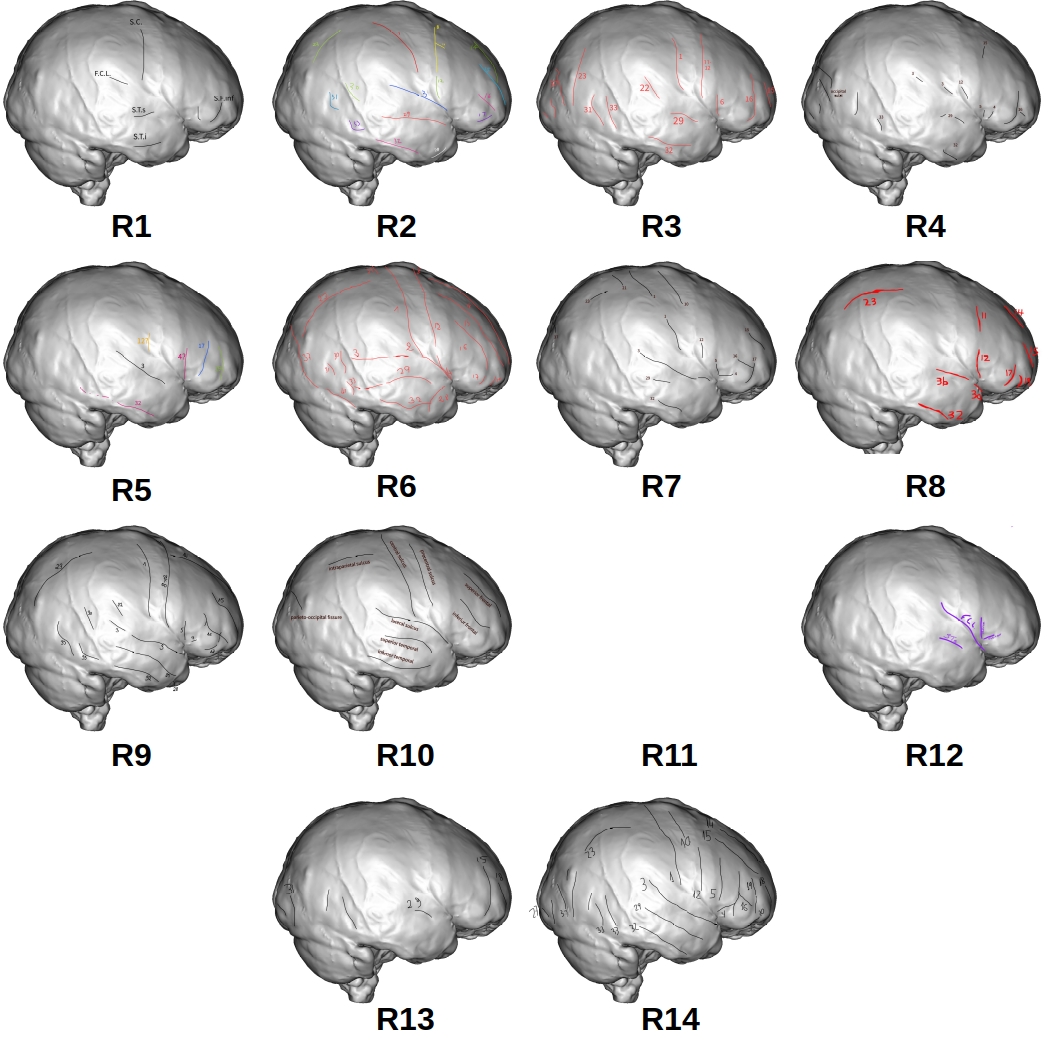


Figure S2. View of the right-hemisphere drawings produced by each researcher participating in this study. Note that R11 has not identified imprints in this view and has not sent the file explaining why this image is missing.


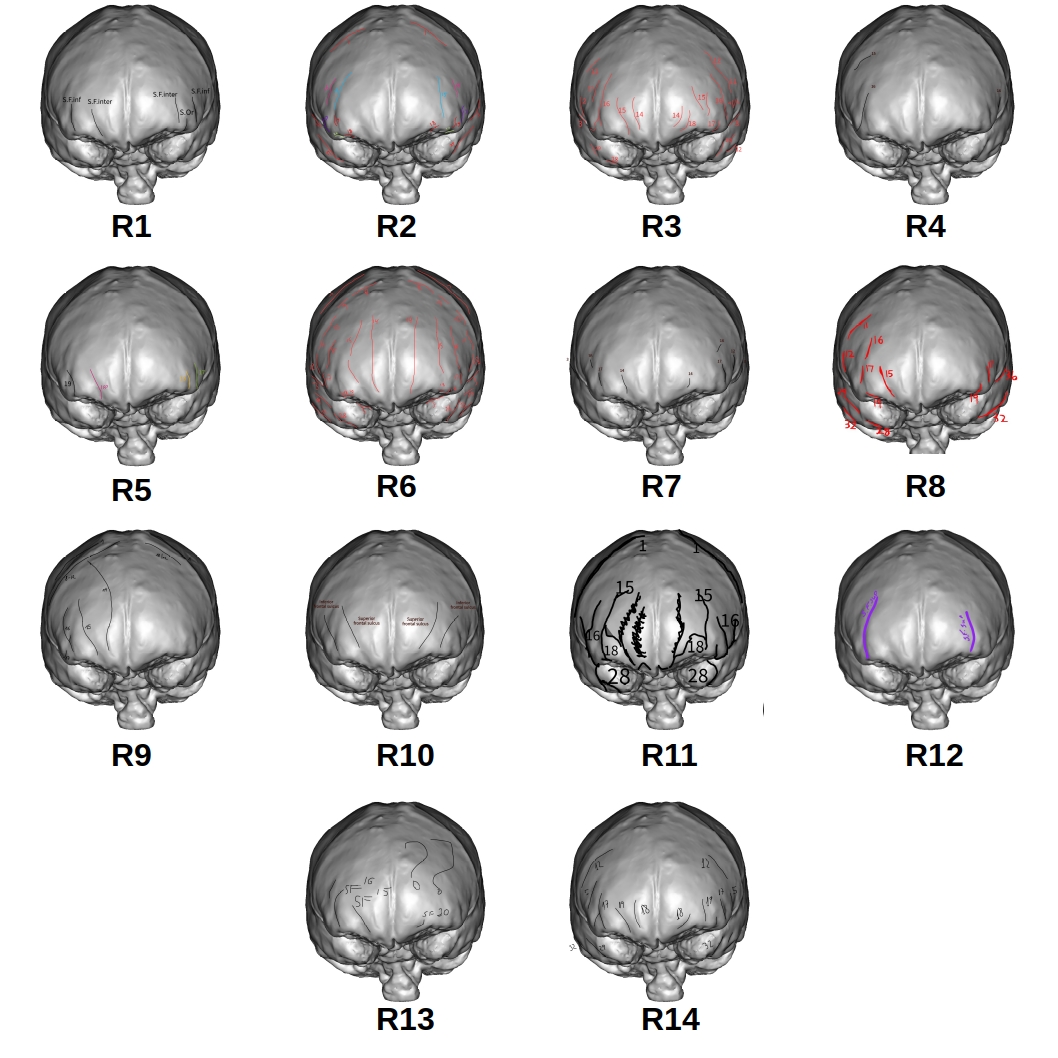


Figure S3. View of the drawings produced in anterior view by each researcher participating in this study.


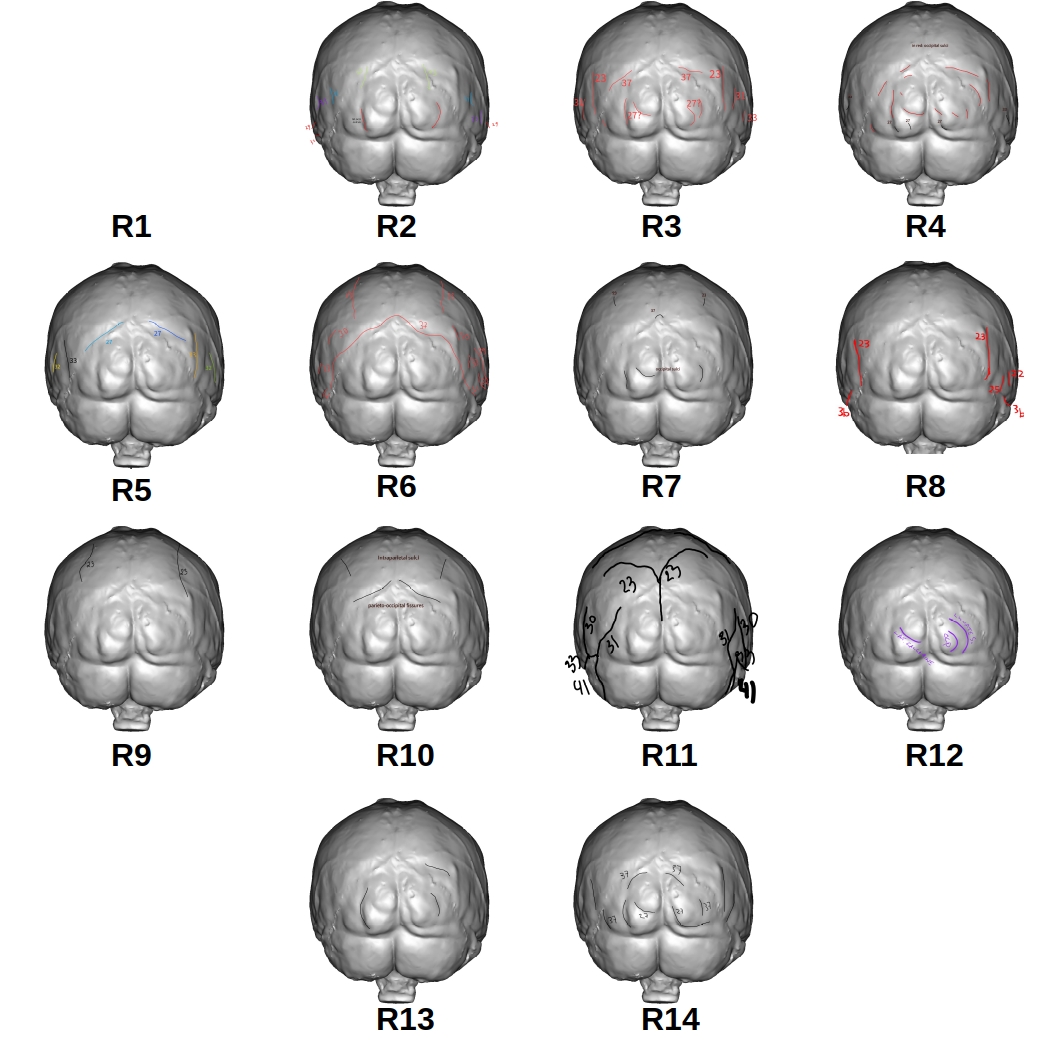


Figure S4. View of the drawings produced in posterior view by each researcher participating in this study. Note that R1 has not identified imprints in this view and has not sent the file explaining why this image is missing.


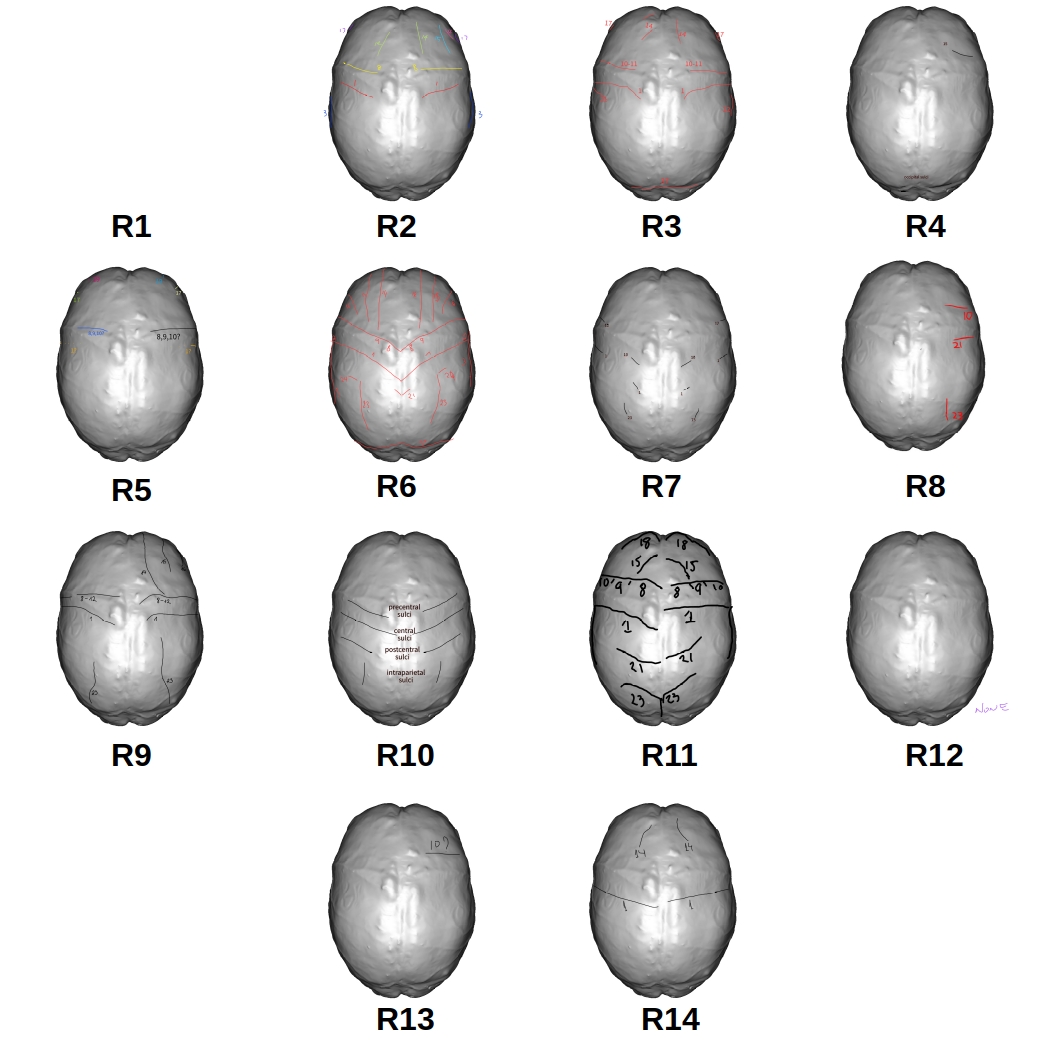


Figure S5. View of the drawings produced in superior view by each researcher participating in this study. Note that R1 has not identified imprints in this view and has not sent the file explaining why this image is missing.

***
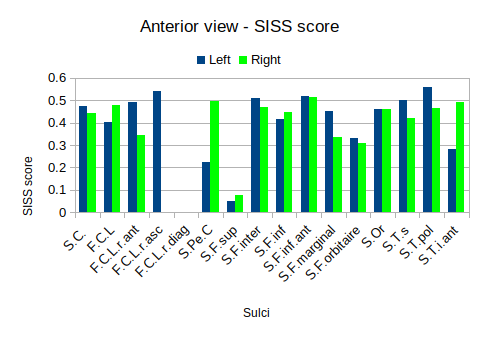

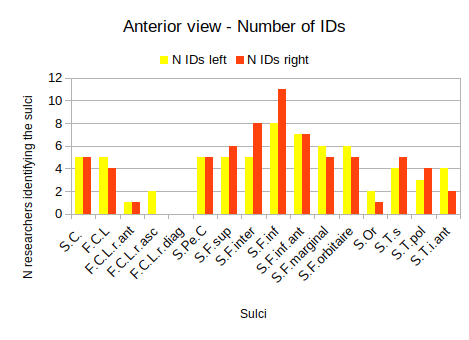
***

***
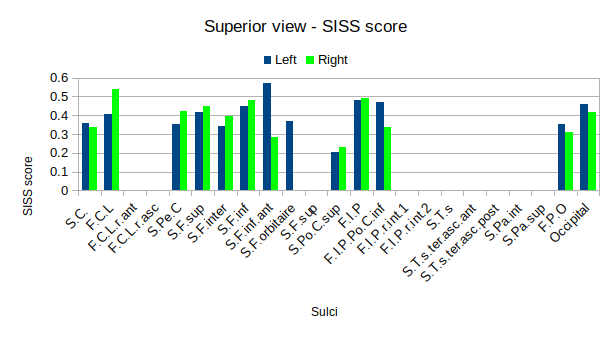

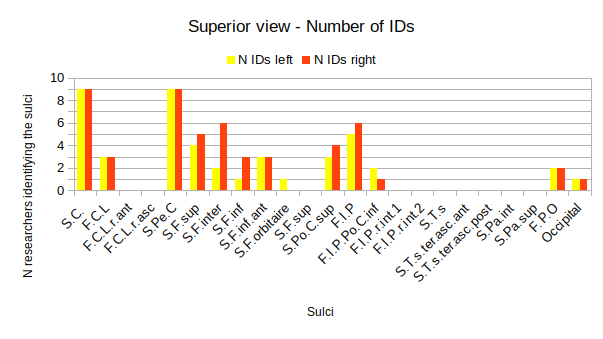
***

***
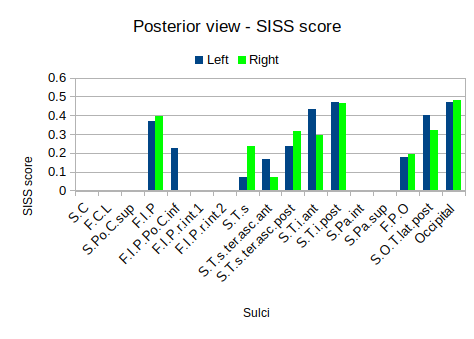

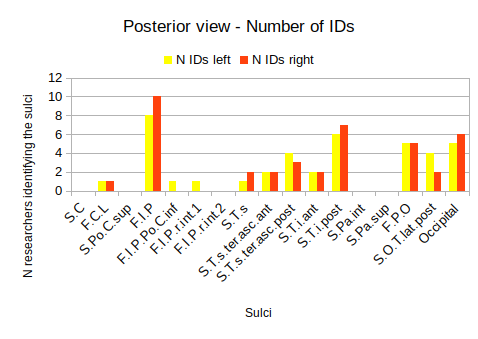
***

***
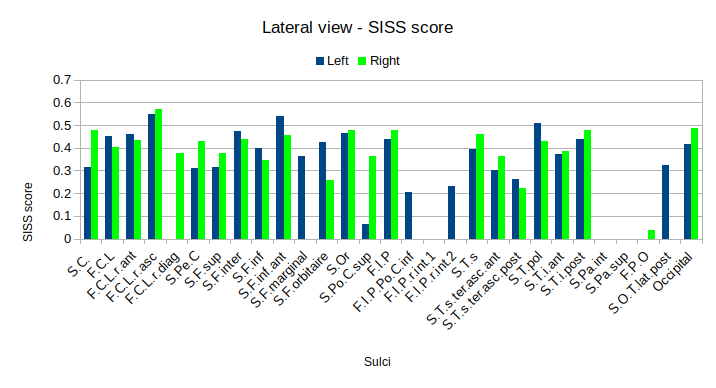

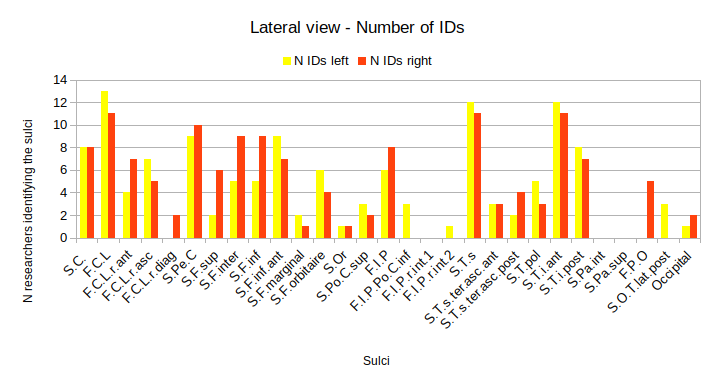
***

Figure S6. On the left, the average SISS score for every sulci in each view is shown. On the right side, the amount of researchers that identified the sulci in each view is presented. Data with the original labelling.


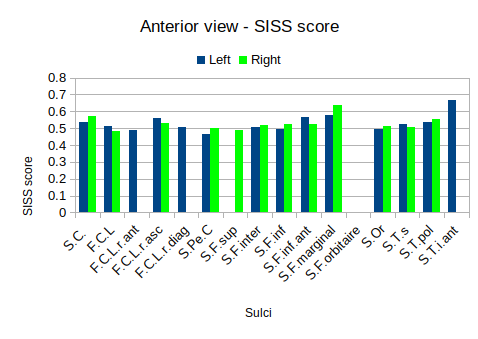

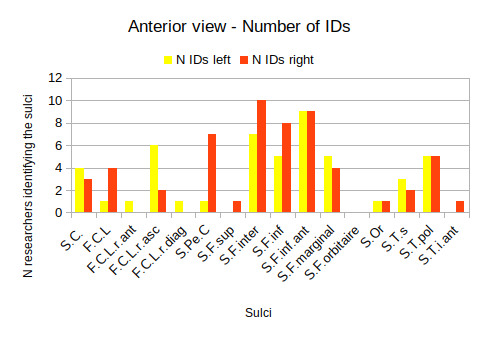

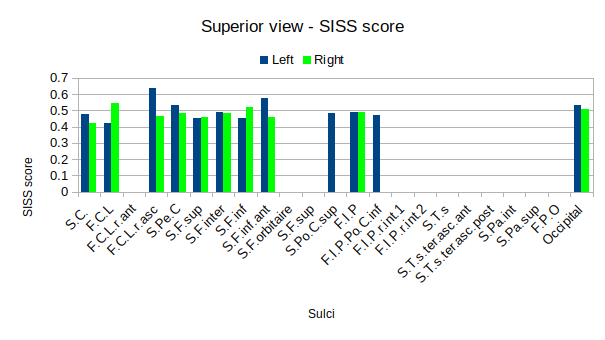

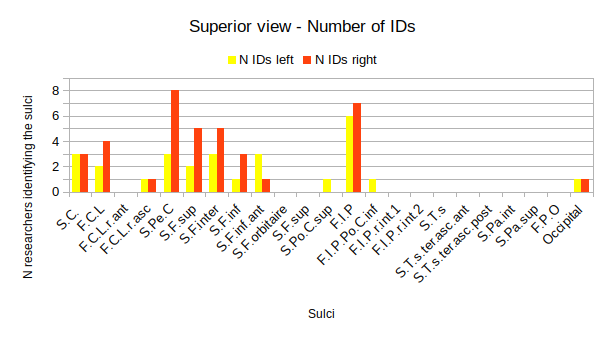


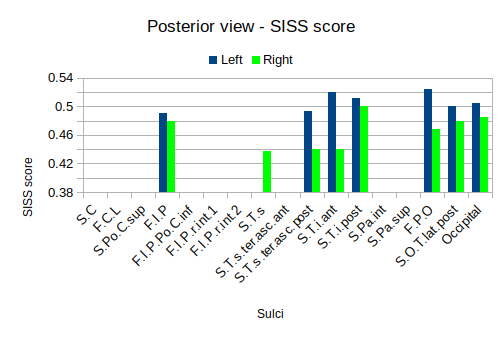

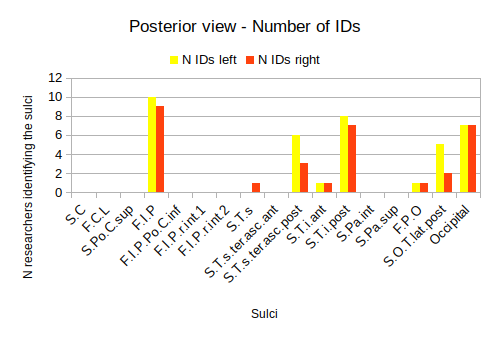

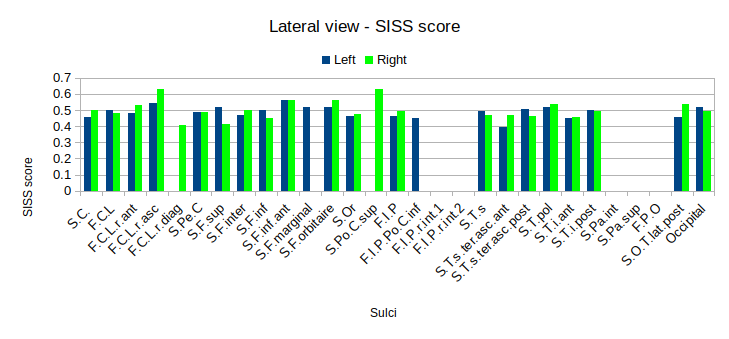

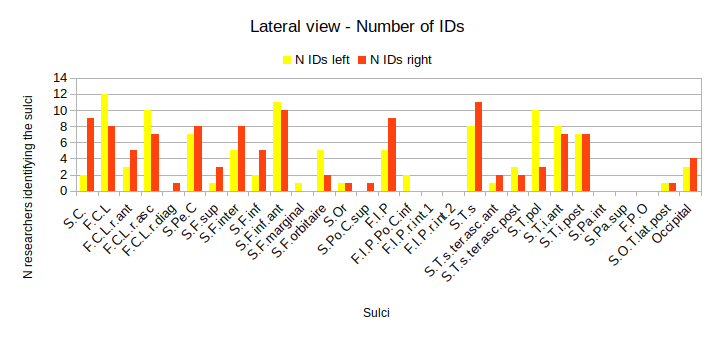


Figure S7. On the left, the average SISS score for every sulci in each view is shown. On the right side, the amount of researchers that identified the sulci in each view is presented. Data with the corrected labelling.
